# Mitotic assembly of respiratory supercomplexes ensures accurate cell division and fuels tumor growth

**DOI:** 10.1038/s44319-026-00937-8

**Published:** 2026-09-23

**Authors:** Ting Wang, Aoxing Cheng, Lingyu Kong, Weiyi You, Jihua Liu, Xing Liu, Xuebiao Yao, Yi Yang, Yuzheng Zhao, Weiwei Yang, Kaiguang Zhang, Zhenye Yang, Jing Guo

**Affiliations:** 1 Department of Digestive Disease, The First Affiliated Hospital of USTC, Division of Life Sciences and Medicine, University of Science and Technology of China, Hefei, Anhui 230001 China; 2 State Key Laboratory of Immune Response and Immunotherapy, Division of Life Sciences and Medicine, University of Science and Technology of China, Hefei, Anhui 230027 China; 3 Ministry of Education Key Laboratory for Membraneless Organelles and Cellular Dynamics, University of Science and Technology of China, Hefei, Anhui 230027 China; 4 Optogenetics & Synthetic Biology Interdisciplinary Research Center, State Key Laboratory of Bioreactor Engineering, Shanghai Frontiers Science Center of Optogenetic Techniques for Cell Metabolism, School of Pharmacy, East China University of Science and Technology, Shanghai, 200237 China; 5 Key Laboratory of Multi-cell Systems, Shanghai Key Laboratory of Molecular Andrology, CAS Center for Excellence in Molecular Cell Science, Shanghai Institute of Biochemistry and Cell Biology, University of Chinese Academy of Sciences, Chinese Academy of Sciences, Shanghai, 200031 China; 6 Institute of Cancer Research, Anhui Key Laboratory of Molecular Oncology, Division of Life Sciences and Medicine, University of Science and Technology of China, Hefei, Anhui 230027 China; 7 Center for Advanced Interdisciplinary Science and Biomedicine of IHM, Division of Life Sciences and Medicine, University of Science and Technology of China, Hefei, Anhui 230027 China

**Keywords:** Cell Cycle, Metabolism, Proteomics

## Abstract

As cells progress from interphase into mitosis, fluctuating metabolic demands coincide with mitochondrial fission. However, the mechanisms by which mitochondria coordinate morphological changes and metabolic adjustments during mitosis remain poorly understood. Using proteomic analysis of BN-PAGE fractions, we show that assembly of the mitochondrial respiratory supercomplexes, comprising electron transport chain complexes I, III, and IV, is markedly enhanced during mitosis in HeLa and MDA-MB-468 cancer cells. Mechanistically, the upregulation of specific CI subunits, including NDUFA3, drives the modular assembly of supercomplexes in mitosis. We further demonstrate that CDK1 promotes the translation of NDUFA3 to enable supercomplex formation. Disruption of this process impairs mitochondrial integrity, energy production, and redox homeostasis, leading to ROS accumulation that triggers mitotic cell death and chromosome segregation defects across multiple models. Importantly, inhibiting mitotic supercomplex assembly induces chromosome mis‑segregation and suppresses tumor growth in vivo. Our findings reveal supercomplex assembly as a key mechanism coordinating mitochondrial fission with metabolic adaptation to support cell division and tumor proliferation in these cancer cell contexts.

## Synopsis

CDK1 stimulates NDUFA3 translation in cancer cells to orchestrate mitochondrial respiratory supercomplex assembly during mitosis. This metabolic adaptation couples mitochondrial fission to redox and energy homeostasis, thereby ensuring faithful chromosome segregation and sustaining tumor growth.

- Mitochondrial respiratory supercomplex assembly is dynamically upregulated during mitosis.
- CDK1 upregulates NDUFA3 translation to promote mitotic respiratory supercomplex assembly.
- Mitotic respiratory supercomplexes maintain redox homeostasis and chromosome stability.
- Disruption of mitotic respiratory supercomplex assembly suppresses tumor growth in vivo.

## Abstract (web summary)

CDK1 stimulates NDUFA3 translation in cancer cells to orchestrate mitochondrial respiratory supercomplex assembly during mitosis. This metabolic adaptation couples mitochondrial fission to redox and energy homeostasis, thereby ensuring faithful chromosome segregation and sustaining tumor growth.

## Graphical abstract

**Figure:**
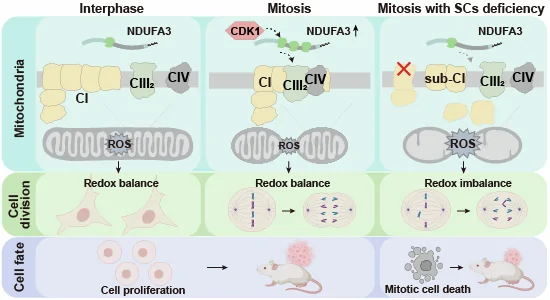

## Introduction

In each cell cycle phase, cells adjust metabolism to meet the different metabolic demands of biosynthesis and energy, which also requires oscillation of mitochondrial metabolism (Diehl et al, 2024). Mitochondrial morphology is also changed during the cell cycle (Giacomello et al, 2020). While mitochondrial fission renders redistribution equal amounts of the organelles into daughter cells in mitosis (Kashatus et al, 2011), mitochondrial fusion supports various metabolism in interphase (Mitra et al, 2009). Mitochondrial membrane dynamics is fundamental for energy generation. In general, the efficiency of ATP production is increased in the fused mitochondria, whereas the fragmented mitochondria generate less ATP (Giacomello et al, 2020), which seems not reconciled with high ATP demand in mitosis. Accumulating evidence indicates that mitochondrial respiration is coordinated with mitotic cell cycle progression (Xie et al, 2019). For instance, CDK1 phosphorylates Tom6 (Harbauer et al, 2014) or mitochondrial complex subunits by entering mitochondria to promote mitochondrial biogenetics during mitosis (Wang et al, 2014). However, the mechanism underlying mitochondrial metabolism cooperating with the mitotic progression remains to be elucidated.

The proper assembly and function of the electron transport chain (ETC) are essential to maintain mitochondrial respiration. The ETC consists of four protein complexes (complex I–IV; CI–IV) embedded in the inner mitochondrial membrane, which transfers electrons from substrates to oxygen, generating a proton gradient (Vercellino and Sazanov, 2022). Individual ETC complexes can also form higher-order assemblies into supercomplexes (SCs) of different stoichiometry and structure (Gu et al, 2016; Guo et al, 2017; Letts et al, 2016). The most common supercomplex is composed of CI, CIII and CIV, which is also called mitochondrial respirasome. Supercomplexes have been shown to enhance individual complexes stability (Krishna et al, 2021), transfer electrons more efficiently (Berndtsson et al, 2020), limit ROS production and protect vulnerable components of ETC complexes from oxidative damage (Liang et al, 2025; Lopez-Fabuel et al, 2016). Respiratory supercomplex formation is dynamically regulated in response to metabolic demands and is implicated in various diseases, including cancer (Chen et al, 2023; Hollinshead et al, 2020; Ikeda et al, 2019; Nagano et al, 2018; Yang et al, 2024). Therefore, targeting supercomplex formation could be a promising therapeutic strategy for cancer. However, it remains unclear how supercomplex formation adapts to the metabolic demands of the cell cycle.

In this study, we demonstrate that the assembly of mitochondrial respiratory supercomplexes allows HeLa and MDA-MB-468 cancer cells to meet the metabolic demands of mitosis. Upregulation of specific ETC subunits facilitates a stepwise assembly of supercomplexes during mitotic progression. Disrupting supercomplex assembly leads to mitochondrial dysfunction, increased glycolysis, compromised cristae morphology, ROS overproduction, which impaired chromosome segregation and caused mitotic cell death, and ultimately inhibited tumor cell growth. These findings uncover a mitotic metabolic vulnerability governed by CDK1-dependent regulation of ETC subunits translation and supercomplex assembly. Our work underscores the essential role of supercomplex assembly in mitosis within these cancer cell models and provides a mechanistic basis for targeting this process as a therapeutic strategy.

## Results

### Mitotic cancer cells exhibit robust upregulation of respiratory supercomplex assembly

**Figure 1:**
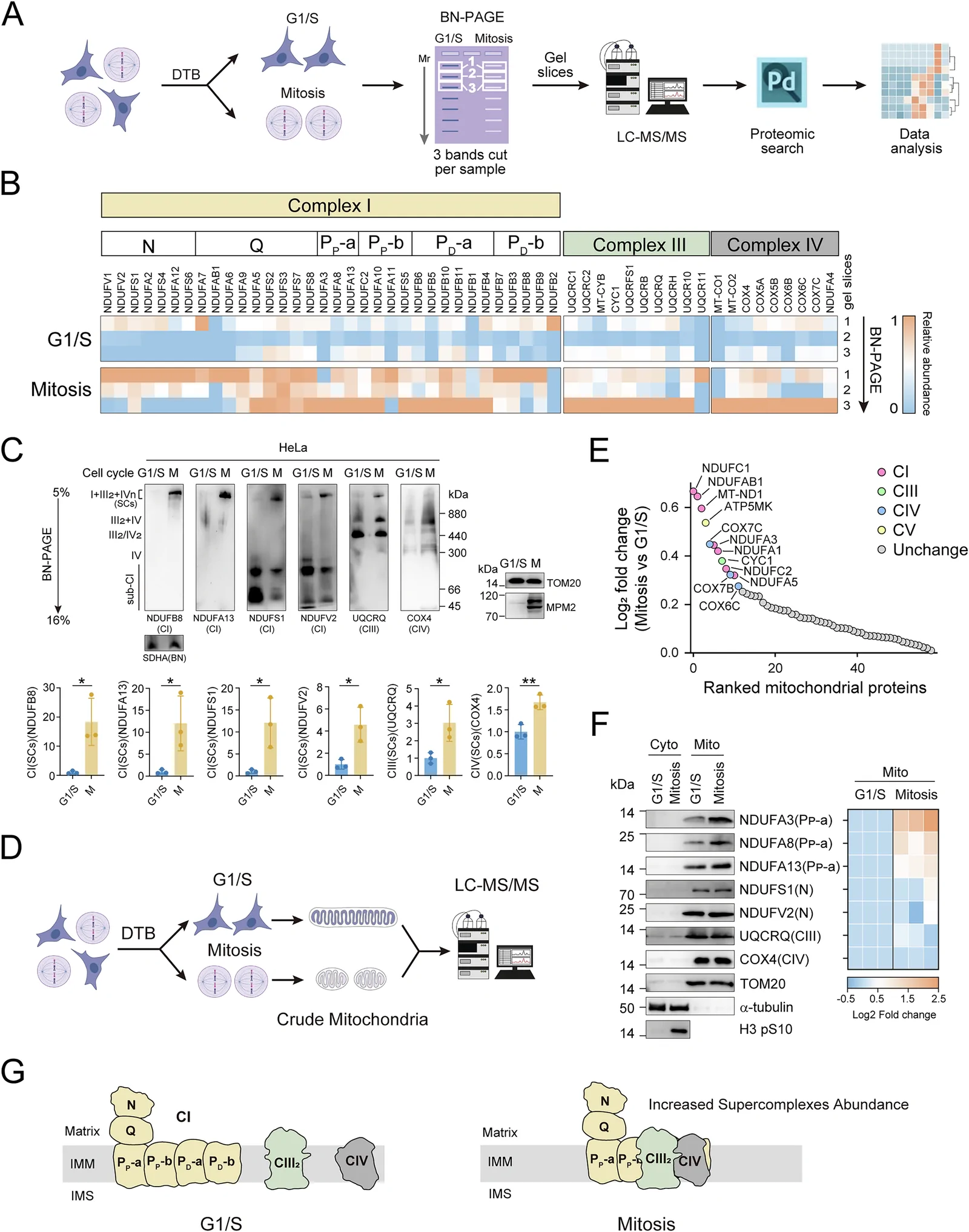
Mitotic cancer cells exhibit robust upregulation of respiratory supercomplex assembly. (**A**) Experimental workflow for the study of supercomplexes proteomics. Isolated mitochondria from G1/S and mitosis HeLa cells and run in a BN-PAGE gel. Individual bands representing supercomplexes were excised and digested by in-gel digestion prior to LC-MS/MS measurements and data analysis. (**B**) Heatmaps showing proteomic analysis of BN-PAGE fractions represent the average steady-state abundance of total CI/CIII/CIV and individual CI subunits plotted against their apparent molecular weights (MW) in the mitochondria isolated from G1/S and mitosis HeLa cells. Positions of supercomplexes are indicated. (**C**) BN-PAGE of the OXPHOS complexes in the isolated mitochondria from G1/S and mitosis HeLa cells (top). Mature supercomplex forms of CI (NDUFB8, NDUFA13, NDUFS1, NDUFV2), CIII (UQCRQ), and CIV (COX4) were quantified (*n* = 3 biologically independent experiments) (bottom). Data with error bars are means ± SD. *P  = 0.0206 (NDUFB8), *P  = 0.0377 (NDUFA13), *P  = 0.0281 (NDUFS1), *P  = 0.0193 (NDUFV2), *P  = 0.0338 (UQCRQ), **P  = 0.0076 (COX4). *P* values are determined using unpaired two-tailed Student’s *t* test. SDHA and TOM20 were used as loading controls. MPM2 was used as a mitosis marker. (**D**) Experimental workflow of quantification and comparison of mitoproteomes in the isolated mitochondria from G1/S and mitosis HeLa cells. (**E**) Scatter plot of the mass spectrometry results showing fold changes in all identified proteins in mitosis as compared to G1/S HeLa cells. Pink (CI), green (CIII), blue (CIV) and yellow (CV) points denote the upregulated proteins with fold change ≥1.2, and grey points denote the unchanged proteins. (**F**) Western blotting analysis of the protein markers of OXPHOS complexes in the isolated mitochondria from G1/S and mitosis HeLa cells (left). Heatmap illustrating log_2_ fold change in mitochondrial ETC protein abundance in mitosis as compared to G1/S HeLa cells (*n* = 3 biologically independent experiments) (right). (**G**) A schematic model for the different supercomplex abundance during G1/S and mitosis (Guerrero-Castillo et al, 2017). Source data are available online for this figure.

**Figure EV1:**
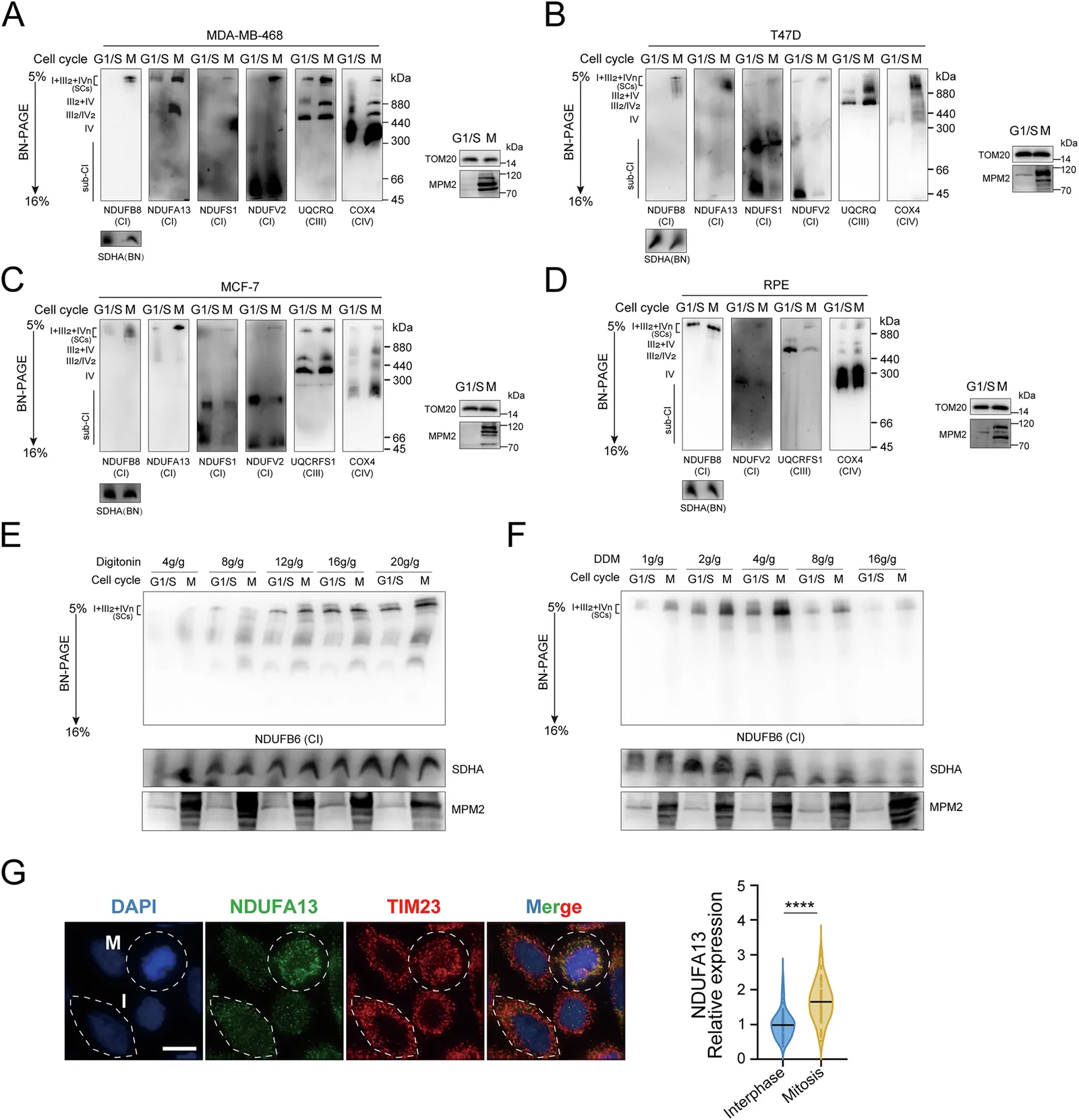
Mitotic cancer cells exhibit robust upregulation of respiratory supercomplex assembly. (**A**–**D**) BN-PAGE of the OXPHOS complexes in the isolated mitochondria from G1/S and mitosis MDA-MB-468 (**A**), T47D (**B**), MCF-7 (**C**), RPE (**D**) cells. Mature supercomplex forms of CI (NDUFB8, NDUFA13, NDUFS1, NDUFV2), CIII (UQCRQ or UQCRFS1), and CIV (COX4) were examined. SDHA and TOM20 were used as loading controls. MPM2 was used as a mitosis marker. (**E**) BN-PAGE of the OXPHOS complexes in the isolated mitochondria from G1/S and mitosis HeLa cells using indicated concentrations of digitonin. Mature supercomplex form of CI (NDUFB6) was examined. SDHA was used as a loading control. MPM2 was used as a mitosis marker. (**F**) BN-PAGE of the OXPHOS complexes in the isolated mitochondria from G1/S and mitosis HeLa cells using indicated concentrations of DDM. Mature supercomplex form of CI (NDUFB6) was examined. SDHA was used as a loading control. MPM2 was used as a mitosis marker. (**G**) Representative immunofluorescent images of HeLa cells stained for NDUFA13 (green), mitochondria (TIM23, red) and DNA (DAPI, blue) (left), and quantification of endogenous NDUFA13 and TIM23 in G1/S and mitosis HeLa cells (*n* = 63 biologically independent cells) (right). Data is shown as violin plots. ****P  = 1.78×10^−12^. *P* values are determined using unpaired two-tailed Student’s *t* test. Scale bar indicates 10 μm. The broken white lines highlight interphase cells (I) and mitotic cells (M).

To investigate the dynamics of the mitochondrial electron transport chain supercomplexes across different cell cycle phases, we isolated mitochondria from HeLa cells synchronized at interphase (G1/S) and mitosis, and resolved them by blue native polyacrylamide gel electrophoresis (BN-PAGE) (Fig. 1A). Proteomic analysis was performed on three high-molecular-weight BN-PAGE fractions (> 880 kDa), corresponding to the expected migration of the supercomplexes (Fig. 1A). Quantitative mass spectrometry revealed that the levels of specific ETC subunits from complexes CI, CIII, and CIV were significantly elevated in mature supercomplexes from mitotic cells compared to those in interphase cells (Fig. 1B), suggesting a marked upregulation of supercomplex assembly during mitosis. To further validate cell cycle‑dependent changes in supercomplex assembly, we performed BN-PAGE followed by immunoblotting using antibodies against multiple subunits of the three mitochondrial complexes (CI: NDUFB8, NDUFA13, NDUFS1, NDUFV2; CIII: UQCRQ; CIV: COX4). Consistent with the proteomic data, mitotic cells exhibited a pronounced increase in supercomplex levels containing subunits from CI, CIII, and CIV (Fig. 1C). Similar trends were observed in several breast cancer cell lines (MDA-MB-468, T47D, MCF-7), while the effect was less pronounced in non-transformed RPE cells (Fig. EV1A–D). Across all tested detergent conditions (4–20 g/g digitonin and 1–16 g/g DDM), the mitotic enrichment of supercomplexes was consistently observed, confirming that the patterns are not preparation artefacts (Fig. EV1E,F). Collectively, these results demonstrate that supercomplex assembly is enhanced during mitosis in cervical and breast cancer cells.

### Specific CI subunits are upregulated during mitosis

To distinguish whether the elevated supercomplex levels in mitosis result solely from enhanced supercomplex assembly or are also driven by an increase in the abundance of electron transport chain (ETC) subunits, we performed a comparative proteomic analysis to quantify ETC proteins in interphase and mitotic cells (Fig. 1D). We identified 66 of the 73 known subunits spanning CI–CV, achieving ~90% coverage. Among these, 12 ETC subunits were upregulated in mitosis (Fig. 1E), with subunits belonging to the P_P_-a module of CI showing the most pronounced increase. Immunoblot analysis of mitochondrial respiratory complexes from purified mitotic HeLa mitochondria further confirmed a marked upregulation of multiple CI proteins during mitosis (Fig. 1F). In particular, protein levels of several CI components, specifically NDUFA3, NDUFA8, and NDUFA13 from the P_P_‑a module, were strongly elevated in mitotic cells, whereas the expression of other CI, CIII, and CIV subunits remained largely unchanged (Fig. 1F). Immunofluorescence experiments also revealed a substantial increase in NDUFA13 levels in mitotic cells (Fig. EV1G). Together, these findings indicate that enhanced supercomplex assembly during mitosis is accompanied by elevated expression of specific ETC subunits, most notably those within the P_P_‑a module of CI (Fig. 1G).

### NDUFA3 is essential for supercomplex assembly during mitosis

**Figure 2:**
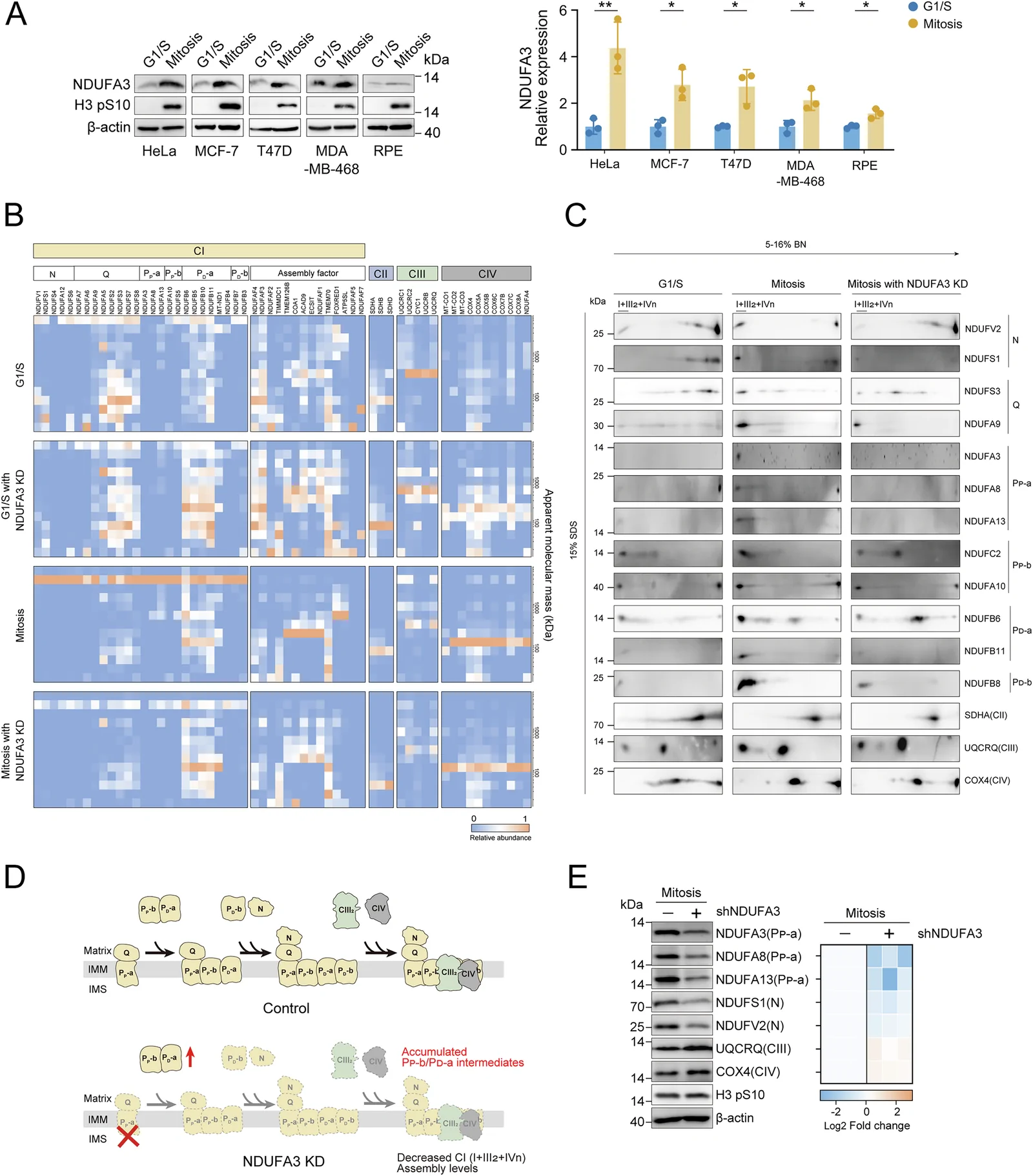
NDUFA3 is essential for supercomplex assembly during mitosis. (**A**) Western blotting analysis of the NDUFA3 protein in G1/S and mitosis HeLa, MCF-7, T47D, MDA-MB-468, RPE cells (left). Quantified data are presented (*n* = 3 biologically independent experiments) (right). Data with error bars are means ± SD. **P  = 0.0072 (HeLa), *P  = 0.0133 (MCF-7), *P  = 0.0152 (T47D), *P  = 0.0187 (MDA-MB-468), *P  = 0.0111 (RPE). *P* values are determined using unpaired two-tailed Student’s *t* test. (**B**) Heatmaps showing proteomic analysis of BN-PAGE fractions represent the average steady-state abundance of total CI/CIII/CIV against their apparent molecular weights (MW) in the mitochondria isolated from G1/S and mitosis HeLa cells with or without NDUFA3 knockdown. Positions of supercomplexes are indicated. (**C**) 2D-BN/SDS–PAGE, Western blot, and immunodetection analysis of G1/S, mitosis, and mitosis with NDUFA3 knockdown mitochondria from HeLa cells to follow the incorporation kinetics of the indicated CI nuclear-encoded subunits, belonging to different structural modules, CII (SDHA), CIII (UQCRQ) and CIV (COX4). The blots shown were either exposed for the same time in order to visualize the qualitative signals. (**D**) A schematic model for CI and supercomplex assembly proceeds through a stepwise incorporation of preassembled intermediates (Guerrero-Castillo et al, 2017) (top). A schematic model for loss of NDUFA3 in mitotic cells results in accumulated P_P_-b/P_D_-a assembly intermediates, decreased P_D_-b and N assembly intermediates, decreased CI and supercomplex assembly (bottom). (**E**) Western blotting analysis of the protein markers of ETC complexes of Control and NDUFA3 KD mitotic HeLa cells with the indicated antibodies (left). Heatmap illustrating log_2_ fold change in mitochondrial ETC protein abundance in mitosis HeLa cells (*n* = 3 biologically independent experiment) (right). Source data are available online for this figure.

**Figure EV2:**
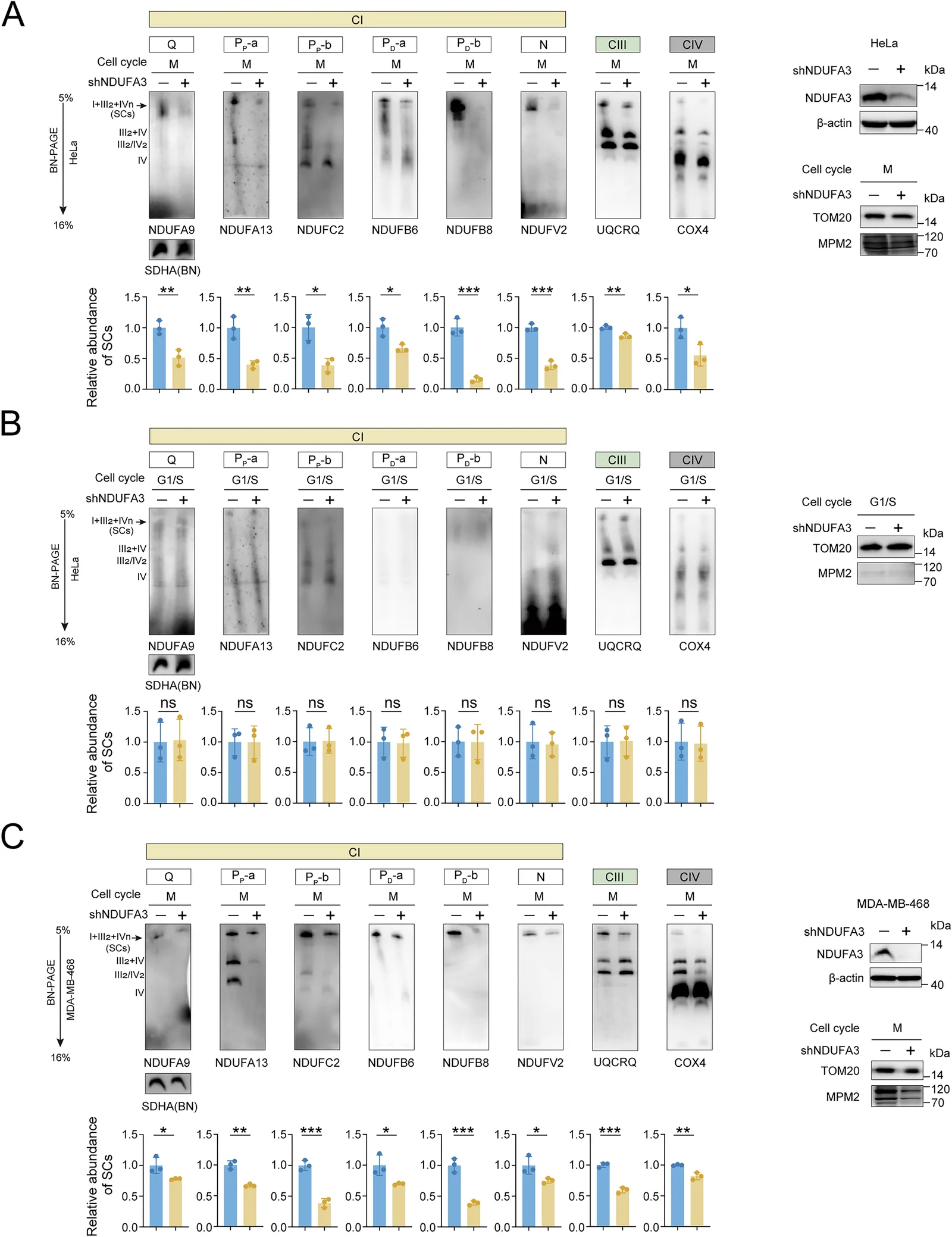
NDUFA3 is essential for supercomplex assembly during mitosis. (**A**) BN-PAGE immunoblots (left) for the ETC complexes and supercomplexes of Control and NDUFA3 KD mitotic HeLa cells, shown by levels of NDUFA9 (Q), NDUFA13 (P_P_-a), NDUFC2 (P_P_-b), NDUFB6 (P_D_-a), NDUFB8 (P_D_-b), NDUFV2 (N), and UQCRQ (CIII), COX4 (CIV). Western blot analysis of cell lysates from Control or NDUFA3 KD HeLa cells with the indicated antibodies (right). SDHA and TOM20 were used as loading controls. MPM2 was used as a mitosis marker. Quantified data are presented (*n* = 3 biologically independent experiments) (bottom). Data with error bars are means ± SD. **P  = 0.0079 (NDUFA9), **P  = 0.0057 (NDUFA13), *P  = 0.0112 (NDUFC2), *P  = 0.0189 (NDUFB6), ***P  = 0.0006 (NDUFB8), ***P  = 0.0003 (NDUFV2), **P  = 0.0070 (UQCRQ), *P  = 0.0330 (COX4). *P* values are determined using unpaired two-tailed Student’s *t* test. (**B**) BN-PAGE immunoblots (left) for the ETC complexes and supercomplexes of Control and NDUFA3 KD G1/S HeLa cells, shown by levels of NDUFA9 (Q), NDUFA13 (P_P_-a), NDUFC2 (P_P_-b), NDUFB6 (P_D_-a), NDUFB8 (P_D_-b), NDUFV2 (N), and UQCRQ (CIII), COX4 (CIV) (right). SDHA and TOM20 were used as loading controls. MPM2 was used as a mitosis marker. Quantified data are presented (*n* = 3 biologically independent experiments) (bottom). Data with error bars are means ± SD. (ns) *P*  = 0.9065 (NDUFA9), (ns) *P*  = 0.9911 (NDUFA13), (ns) *P*  = 0.9539 (NDUFC2), (ns) *P*  = 0.9339 (NDUFB6), (ns) *P*  = 0.9708 (NDUFB8), (ns) *P*  = 0.8434 (NDUFV2), (ns) *P*  = 0.9514 (UQCRQ), (ns) *P*  = 0.8959 (COX4). *P* values are determined using unpaired two-tailed Student’s *t* test. (**C**) BN-PAGE immunoblots (left) for the ETC complexes and supercomplexes of Control and NDUFA3 KD mitotic MDA-MB-468 cells, shown by levels of NDUFA9 (Q), NDUFA13 (P_P_-a), NDUFC2 (P_P_-b), NDUFB6 (P_D_-a), NDUFB8 (P_D_-b), NDUFV2 (N), and UQCRQ (CIII), COX4 (CIV). Western blot analysis of cell lysates from Control or NDUFA3 KD MDA-MB-468 cells with the indicated antibodies (right). SDHA and TOM20 were used as loading controls. MPM2 was used as a mitosis marker. Quantified data are presented (*n* = 3 biologically independent experiments) (bottom). Data with error bars are means ± SD. *P  = 0.0445 (NDUFA9), **P  = 0.0012 (NDUFA13), ***P  = 0.0006 (NDUFC2), *P  = 0.0343 (NDUFB6), ***P  = 0.0008 (NDUFB8), *P  = 0.0453 (NDUFV2), ***P  = 0.0003 (UQCRQ), **P  = 0.0061 (COX4). *P* values are determined using unpaired two-tailed Student’s *t* test.

We next asked whether the upregulation of specific CI subunits is required for enhanced supercomplex assembly in mitosis. Among the upregulated subunits, NDUFA3—a 9 kDa accessory subunit of CI—was consistently elevated during mitosis across three independent studies involving four distinct cell lines, including HeLa cells (Ly et al, 2017; Stumpf et al, 2013; Tanenbaum et al, 2015). We further confirmed increased NDUFA3 protein levels during mitosis in several additional cancer and non-transformed cell lines (Fig. 2A). Given its reported role in early-stage CI assembly (Rak and Rustin, 2014), we hypothesized that NDUFA3 is critical for supercomplex formation during the cell cycle. To test this, we performed proteomic analysis of BN-PAGE fractions and 2D BN/SDS–PAGE on mitochondria from G1/S and mitotic cells with or without NDUFA3 knockdown. Proteomic analysis of BN-PAGE fractions revealed that NDUFA3 depletion caused pronounced supercomplex disassembly specifically in mitosis, with no significant effect in interphase (Fig. 2B). 2D BN/SDS–PAGE immunoblotting targeting distinct CI modules further confirmed reduced supercomplexes containing CI, CIII, and CIV in mitosis, accompanied by accumulation of CIII₂ and CIV (Fig. 2C). BN-PAGE immunoblotting showed a pronounced reduction in supercomplexes (containing CI, CIII, and CIV subunits) in mitotic HeLa and MDA-MB-468 cells, whereas assembly of individual ETC complexes remained largely unaffected in G1/S-phase cells (Fig. EV2A–C).

CI assembly proceeds through the stepwise integration of preassembled modules: the Q module associates with the P_P_-a module, followed by sequential addition of the P_P_-b and P_D_-a modules. Subsequent incorporation of CIII and CIV leads to supercomplex formation, with the mature supercomplexes finalizing upon integration of the P_D_-b module and the catalytic N module (Fang et al, 2021; Guerrero-Castillo et al, 2017; Nah et al, 2025; Vercellino and Sazanov, 2022; Wang et al, 2021) (Fig. 2D). Within this hierarchical framework, NDUFA3 knockdown caused accumulation of P_P_‑b, P_D_‑a, and Q module-containing intermediates in mitosis, while subunits of the P_D_‑b and N modules failed to assemble into supercomplexes, with no stable intermediates detected in the 100–500 kDa range (Figs. 2B,C and EV2A,C). These data indicate that NDUFA3 is required for early steps of the hierarchical assembly cascade specifically during mitosis. Notably, NDUFA3 depletion during mitosis also significantly reduced protein levels of NDUFA8, NDUFA13, NDUFS1, and NDUFV2 (Fig. 2E), which may further compromise supercomplex assembly. Together, these results demonstrate that NDUFA3 is required for the early assembly of supercomplexes during mitosis but has minimal impact on its formation during interphase.

### CDK1 promotes NDUFA3 translation during mitosis and supports supercomplex assembly

**Figure EV3:**
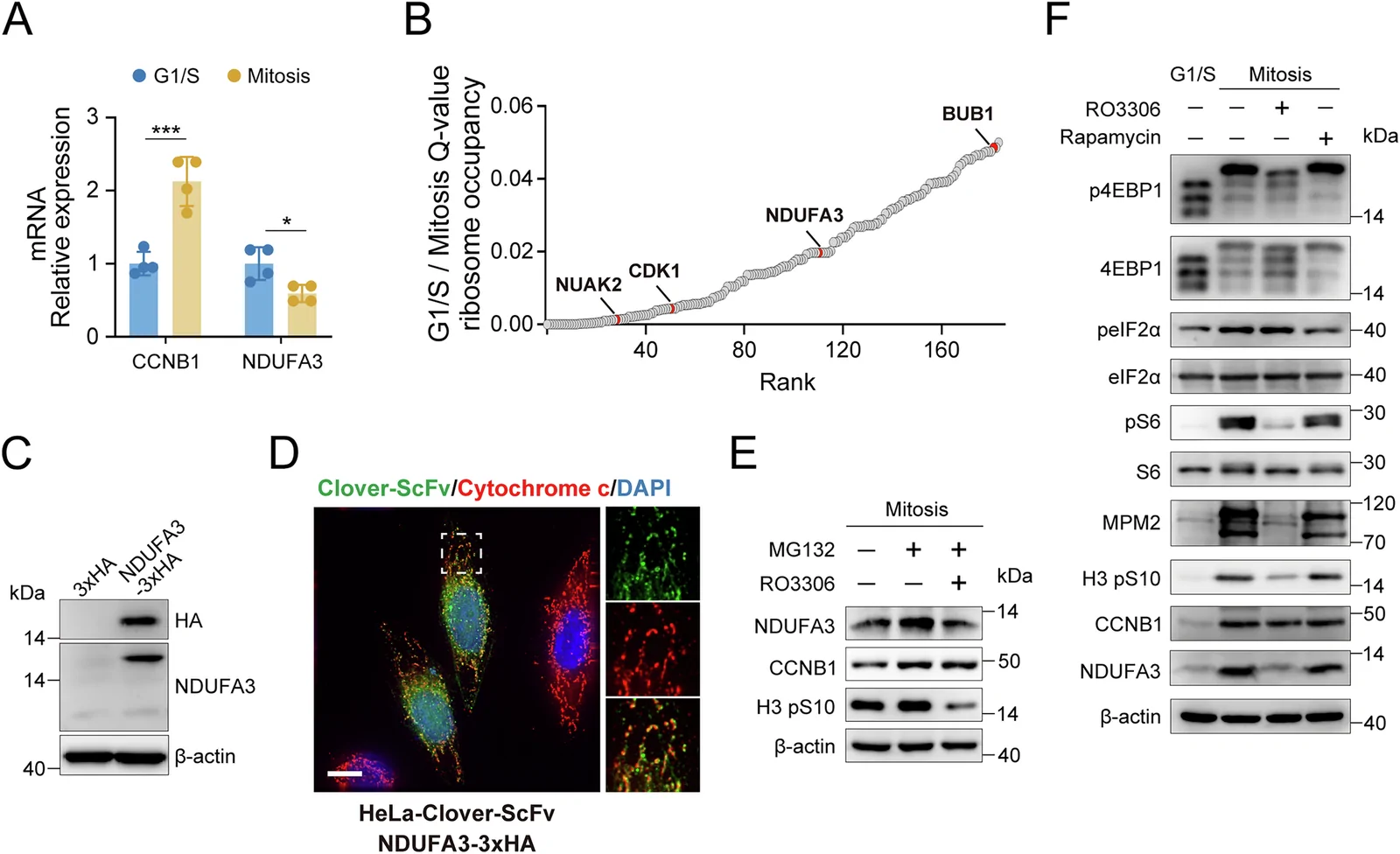
CDK1 promotes NDUFA3 translation during mitosis and supports supercomplex assembly. (**A**) The qPCR analysis of NDUFA3 mRNAs in the G1/S and mitotic HeLa cells (*n* = 4 biologically independent experiments). Data with error bars are means ± SD. ***P  = 0.0009 (CCNB1), *P  = 0.0180 (NDUFA3). *P* values are determined using unpaired two-tailed Student’s *t* test. CCNB1 mRNA was used as a positive control. (**B**) Scatter plot of the q values of ribosome occupancy in G1/S as compared to mitotic HeLa cells(Stumpf et al, 2013). Red points denote the known proteins and NDUFA3 during mitosis. (**C**) Western blot analysis of cell lysates from 3xHA or NDUFA3-3xHA HeLa-Clover-ScFv cells with the indicated antibodies. (**D**) Representative immunofluorescent images of HeLa-Clover-ScFv-NDUFA3-3xHA cells stained for mitochondria (Cytochrome c, red) and DNA (DAPI, blue). Scale bar indicates 10 μm. (**E**) Western blot analysis of cell lysates from mitosis HeLa cells treated with or without MG132 or RO3306 with the indicated antibodies. (**F**) Western blot analysis of cell lysates from G1/S, and mitosis HeLa cells treated with inhibitors against CDK1 and mTOR, and immunoblotting of indicated proteins.

**Figure 3:**
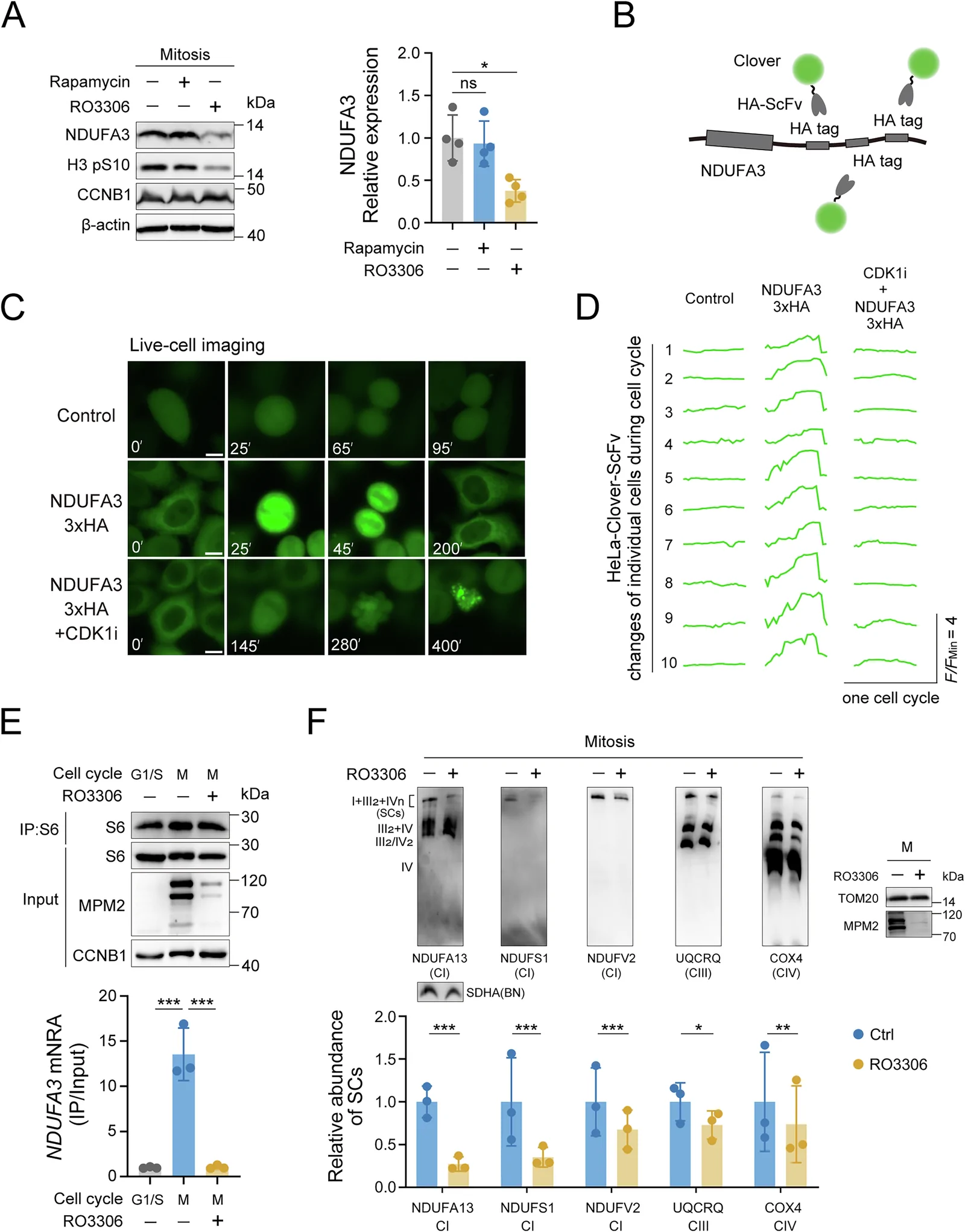
CDK1 promotes NDUFA3 translation during mitosis and supports supercomplex assembly. (**A**) Western blot analysis of cell lysates from mitotic HeLa cells treated with inhibitors against mTOR and CDK1, and immunoblotting of indicated proteins (left). mTORi, Rapamycin; CDK1i, RO3306. CCNB1 and H3 pS10 were used as mitosis markers. Quantified data are presented (*n* = 4 biologically independent experiments) (right). Data with error bars are means ± SD. (ns) *P*  = 0.9142, *P  = 0.0106, from left to right. *P* values are determined using one-way ANOVA. (**B**) Cartoon showing how to screen the anti-HA ScFv (Zhao et al, 2019) in living HeLa cells expressing NDUFA3-3xHA. (**C**) Live-cell imaging of Control, NDUFA3-3xHA or NDUFA3-3xHA with CDK1 inhibitor (RO3306) HeLa-Clover-ScFv cells. Green fluorescence indicates the expression of NDUFA3-3xHA during the cell cycle. Scale bar indicates 10 µm. (**D**) Ten representative expressions of NDUFA3-3xHA dynamics in Control, NDUFA3-3xHA or NDUFA3-3xHA with CDK1 inhibitor (RO3306) in HeLa-Clover-ScFv cells during the cell cycle. (**E**) G1/S or mitosis HeLa cells treated with or without RO3306 were collected for ribosomal protein S6-IP assay (top). The amount of NDUFA3 mRNA in ribosomal complex was determined by RT-qPCR (*n* = 3 biologically independent experiments) (bottom). Data with error bars are means ± SD. ***P  = 0.0002, ***P  = 0.0002, from left to right. *P* values are determined using one-way ANOVA. (**F**) BN-PAGE immunoblots for the ETC complexes and supercomplexes of mitotic HeLa cells treated with or without RO3306 (top). Mature supercomplex forms of CI (NDUFA13, NDUFS1, NDUFV2), CIII (UQCRQ), and CIV (COX4) were quantified (*n* = 3 biologically independent experiments) (bottom). Data with error bars are means ± SD. ***P  = 0.0001 (NDUFA13), ***P  = 0.0006 (NDUFS1), ***P  = 0.0007 (NDUFV2), *P  = 0.0144 (UQCRQ), **P  = 0.0023 (COX4). *P* values are determined using unpaired two-tailed Student’s *t* test. SDHA and TOM20 were used as loading controls. MPM2 was used as a mitosis marker. Source data are available online for this figure.

To elucidate the mechanism underlying NDUFA3 upregulation during mitosis, we first excluded transcriptional control, as NDUFA3 mRNA levels decreased in mitotic cells (Fig. EV3A). Ribosome profiling data from a prior study (Stumpf et al, 2013) (Fig. EV3B) indicated enhanced ribosome engagement of NDUFA3 mRNA in mitosis, prompting us to hypothesize that its expression is translationally regulated. Inhibition of two key mitotic kinases, mTOR and CDK1, which are involved in protein synthesis regulation (Imami et al, 2018; Kalous et al, 2020; Shuda et al, 2015) revealed that CDK1 inhibition, but not mTOR inhibition, markedly reduced NDUFA3 protein levels (Fig. 3A). Using live-cell imaging of NDUFA3-3xHA tagged with HA‑ScFv‑Clover (Figs. 3B and EV3C,D), we confirmed that NDUFA3 levels rise significantly in mitosis compared with interphase, and that this increase is suppressed upon CDK1 inhibition (Fig. 3C,D). Ribosome immunoprecipitation with an anti‑S6 antibody further showed increased association of NDUFA3 mRNA with ribosomes in mitosis, an effect abolished by CDK1 inhibition (Fig. 3E). To distinguish whether CDK1 regulates NDUFA3 translation or protein stability, we treated cells with the proteasome inhibitor MG132. Although MG132 elevated NDUFA3 protein levels, concurrent CDK1 inhibition completely suppressed this accumulation (Fig. EV3E), indicating that CDK1 promotes NDUFA3 synthesis at the translational level rather than stabilizing the protein. To anchor the mechanism, we examined eIF2α, 4EBP1, and S6 phosphorylation (Kalous et al, 2020). CDK1 activity correlated with 4EBP1 and S6 but not eIF2α, narrowing the regulatory pathways (Fig. EV3F). Accordingly, CDK1 inhibition impaired supercomplex assembly (Fig. 3F). Together, these results demonstrate that CDK1 is required for the translational upregulation of NDUFA3 during mitosis, which in turn is critical for respiratory complex assembly in the mitotic cancer cells examined here.

### NDUFA3-mediated supercomplex assembly sustains mitochondrial function and redox homeostasis during mitosis

**Figure 4:**
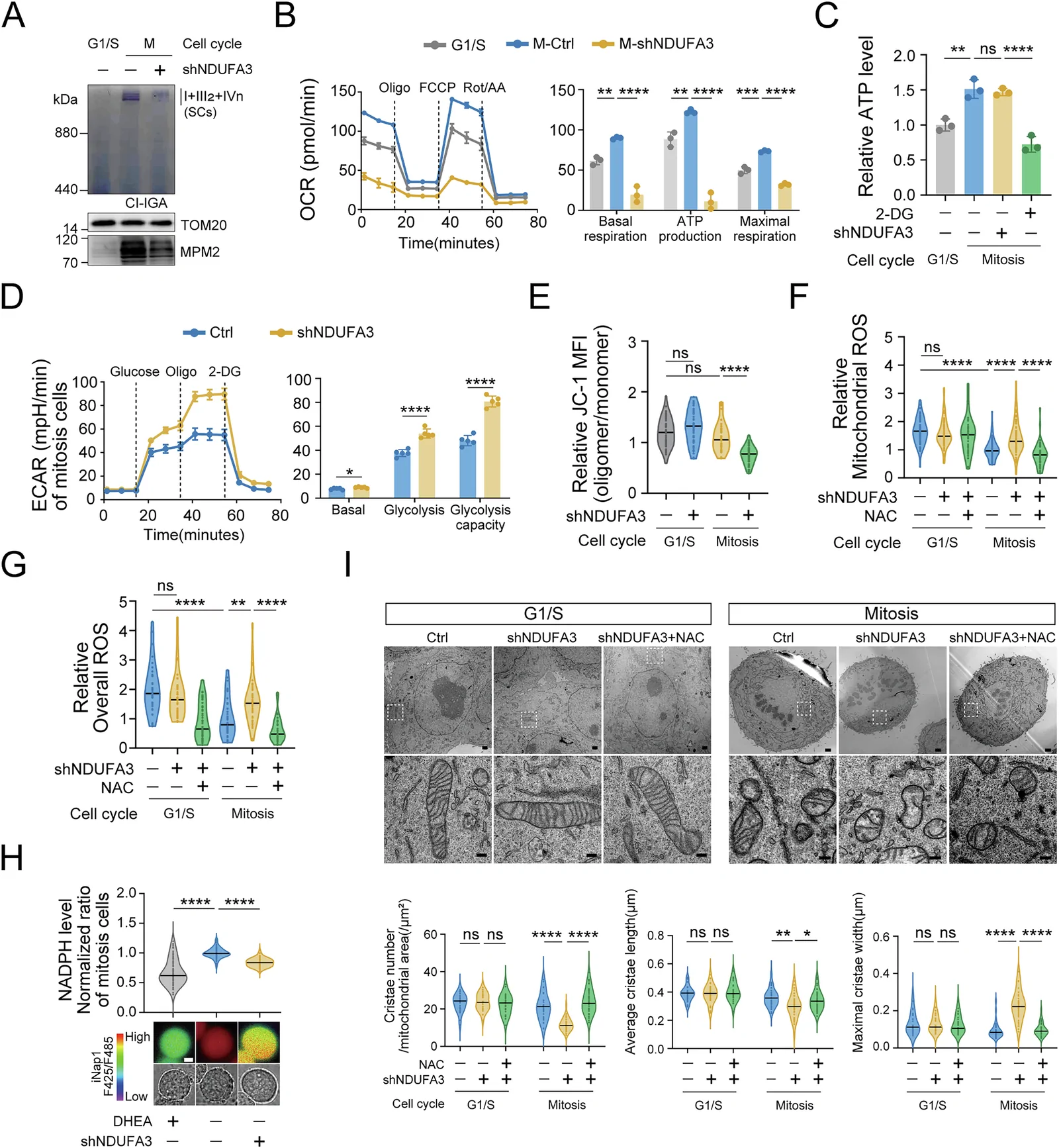
NDUFA3-mediated supercomplex assembly sustains mitochondrial function and redox homeostasis during mitosis. (**A**) In-gel activity of CI and supercomplexes of Control and NDUFA3 KD HeLa cells. TOM20 was used as a loading control. MPM2 was used as a mitosis marker. (**B**) Analysis of oxygen consumption rate (OCR) in Control or NDUFA3 KD HeLa cells. The dotted lines (left) indicate the time of adding 15 μM oligomycin (Oligo), 10 μM FCCP, 5 μM rotenone and antimycin A (Rot/AA). Statistical analysis of basal respiration, ATP production and maximal respiration from Control or NDUFA3 KD HeLa cells (*n* = 3 biologically independent experiments) (right). Data with error bars are means ± SD. **P  = 0.0043 (Basal respiration), ****P  = 2.73 × 10^−5^ (Basal respiration), **P  = 0.0049 (ATP production), ****P  = 6.36 × 10^−6^ (ATP production), ***P  = 0.0001 (Maximal respiration), ****P  = 3.72 × 10^−6^ (Maximal respiration), from left to right. *P* values are determined using one-way ANOVA. (**C**) Intracellular ATP concentrations of Control or NDUFA3 KD upon G1/S or mitotic HeLa cells (*n* = 3 biologically independent experiments). Data with error bars are means ± SD. **P  = 0.0011, (ns) *P*  = 0.9525, ****P  = 7.85 × 10^−5^, from left to right. *P* values are determined using one-way ANOVA. Treatment with 5 mM 2-DG was used as a positive control. (**D**) Analysis of extracellular acidification rate (ECAR) in Control or NDUFA3 KD mitotic HeLa cells. The dotted lines (left) indicate the time of adding 100 mM Glucose, 10 μM oligomycin (Oligo) and 500 mM 2-DG. Statistical analysis of Basal, glycolysis, and glycolysis capacity (right) from Control or NDUFA3 KD mitotic HeLa cells (*n* = 5 biologically independent experiments) (right). Data with error bars are means ± SD. *P  = 0.0224 (Basal), ****P  = 6.04 × 10^−5^ (Glycolysis), ****P  = 2.74×10^−6^ (Glycolysis capacity), from left to right. *P* values are determined using unpaired two-tailed Student’s *t* test. (**E**) Quantification of JC-1 (oligomer/monomer), a membrane potential sensitive binary dye to detect mitochondrial membrane potential (ΔΨm) from Control or NDUFA3 KD G1/S and mitotic HeLa cells, were analyzed based on the live-cell images (*n* = 52, 52, 42, 58 biologically independent cells, from left to right). Data is shown as violin plots. (ns) *P*  = 0.1936, (ns) *P*  = 0.3470, ****P  = 1.17 × 10^−9^, from left to right. *P* values are determined using one-way ANOVA. (**F**) Quantification of MitoSOX to detect mitochondrial ROS from Control or NDUFA3 KD G1/S and mitotic HeLa cells were analyzed based on the live-cell images treated with or without 5 mM NAC (*n* = 82, 70, 49, 82, 70, 51 biologically independent cells, from left to right). Data is shown as violin plots. (ns) *P*  = 0.8362, ****P  < 1 × 10^−15^, ****P  = 1.76 × 10^−6^, ****P  = 5.98 × 10^−10^, from left to right. *P* values are determined using one-way ANOVA. (**G**) Quantification of CM-H_2_DCFDA to detect overall ROS from Control or NDUFA3 KD G1/S and mitotic HeLa cells were analyzed based on the live-cell imaging, treated with or without 5 mM NAC (*n* = 34, 38, 45, 38, 43, 46 biologically independent cells, from left to right). Data is shown as violin plots. (ns) *P*  = 0.7131, ****P  = 3.47 × 10^−8^, **P  = 0.0028, ****P  = 7.29 × 10^−9^, from left to right. *P* values are determined using one-way ANOVA. (**H**) Live-cell imaging of Control or NDUFA3 KD mitotic HeLa cells with iNap1 showed NADPH dynamics. Images were pseudocoloured and indicated F425/F485 ratio (bottom). Scale bar indicates 10 μm. Normalized quantification of NADPH levels from Control or NDUFA3 KD mitotic HeLa cells were analyzed based on the images (*n* = 73, 104, 81 biologically independent cells, from left to right) (top). Data is shown as violin plots. ****P  = 0.79 × 10^−13^, ****P  = 1.23 × 10^−13^, from left to right. *P* values are determined using one-way ANOVA. Treatment with 200 μM DHEA was used as a positive control. (**I**) Representative TEM images of Control and NDUFA3 KD G1/S and mitotic HeLa cells treated with or without 5 mM NAC (top). Scale bars indicate 1 μm (top) and 200 nm (bottom). Quantification (bottom) of cristae number per mitochondria area (left, *n* = 28, 30, 29, 35, 34, 40 biologically independent mitochondrias, from left to right), average cristae length (middle, *n* = 28, 30, 29, 35, 34, 37 biologically independent mitochondrias, from left to right) and maximal cristae width (right, *n* = 27, 30, 30, 35, 33, 40 biologically independent mitochondrias, from left to right), which were counted per condition randomly from images collected in a user-blinded fashion. Data is shown as violin plots. (ns) *P*  = 0.9997 (cristae number), (ns) *P*  = 0.9419 (cristae number), ****P  = 0.16 × 10^−9^ (cristae number), ****P  = 0.99 × 10^−13^ (cristae number), (ns) *P*  > 0.9999 (average cristae length), (ns) *P*  = 0.9609 (average cristae length), **P  = 0.0034 (average cristae length), *P  = 0.0444 (average cristae length), (ns) *P*  > 0.9999 (maximal cristae width), (ns) *P*  = 0.9998 (maximal cristae width), ****P  = 0.44 × 10^−13^ (maximal cristae width), ****P  = 0.45 × 10^−13^ (maximal cristae width), from left to right. *P* values are determined using one-way ANOVA. Source data are available online for this figure.

**Figure EV4:**
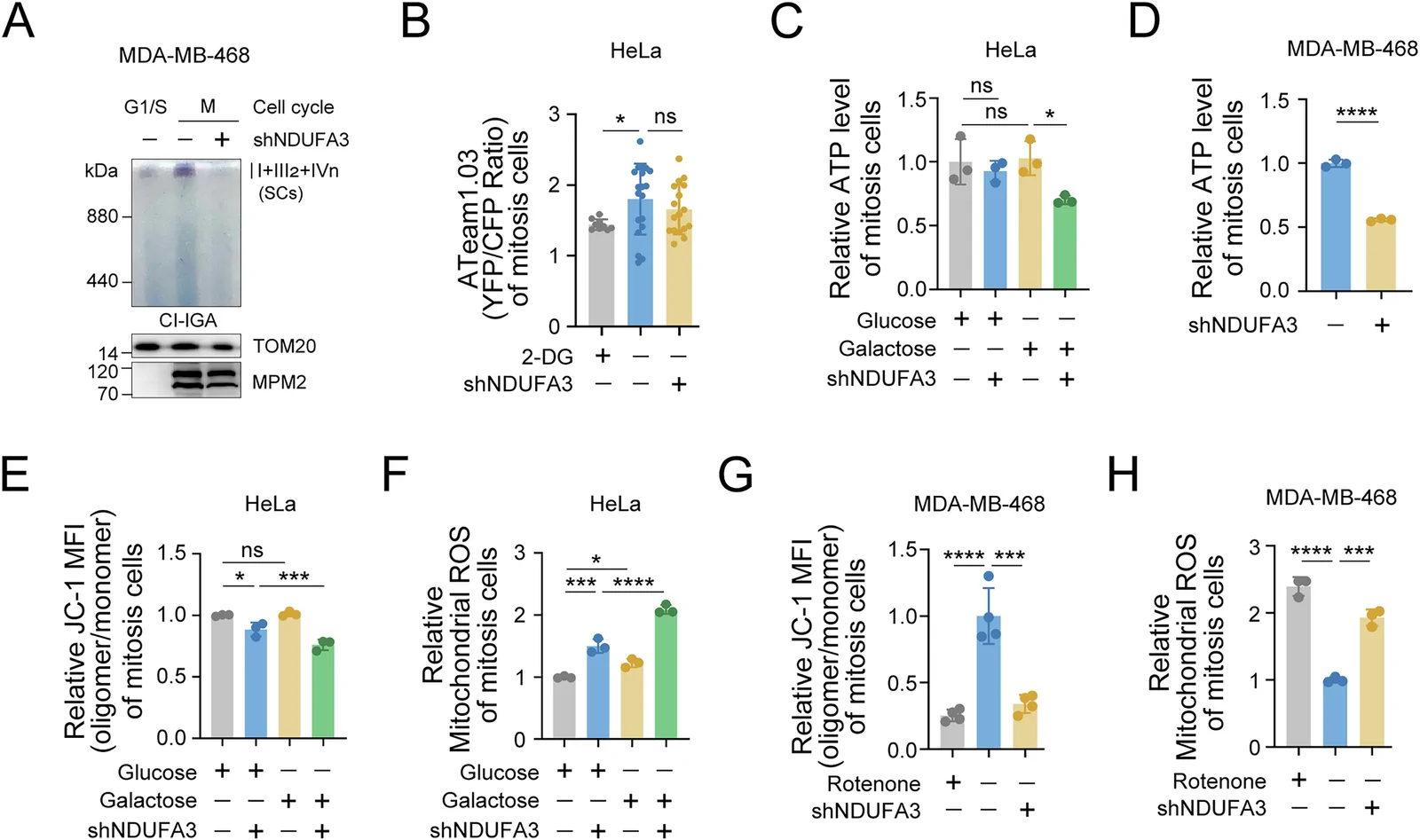
NDUFA3-mediated supercomplex assembly sustains mitochondrial function and redox homeostasis during mitosis. (**A**) In-gel activity of CI and supercomplexes of Control and NDUFA3 KD MDA-MB-468 cells. TOM20 was used as a loading control. MPM2 was used as a mitosis marker. (**B**) Quantification of ATP levels was analyzed based on the pseudocolor ratiometric images of the YFP/CFP ratio from Control or NDUFA3 KD mitotic HeLa cells expressing ATeam1.03 (*n* = 9, 18, 17 biologically independent cells, from left to right). Data with error bars are means ± SD. *P  = 0.0297, (ns) *P*  = 0.2971, from left to right. *P* values are determined using one-way ANOVA. Treatment with 5 mM 2-DG was used as a positive control. (**C**) Intracellular ATP concentrations of Control or NDUFA3 KD upon mitotic HeLa cells cultured under glucose or galactose conditions (*n* = 3 biologically independent experiments). Data with error bars are means ± SD. (ns) *P*  = 0.8741, (ns) *P*  = 0.9905, *P  = 0.0413, from left to right. *P* values are determined using one-way ANOVA. (**D**) Intracellular ATP concentrations of Control or NDUFA3 KD upon mitotic MDA-MB-468 cells (*n* = 3 biologically independent experiments). Data with error bars are means ± SD. ****P  = 1.65 × 10^−5^. *P* value was determined using unpaired two-tailed Student’s *t* test. (**E**) Flow cytometric analysis of JC-1 in Control or NDUFA3 KD mitotic HeLa cells cultured under glucose or galactose conditions (*n* = 3 biologically independent experiments). Data with error bars are means ± SD. *P  = 0.0224, (ns) *P*  = 0.9754, ***P  = 0.0002, from left to right. *P* values are determined using one-way ANOVA. (**F**) Flow cytometric analysis of MitoSOX in Control or NDUFA3 KD mitotic HeLa cells cultured under glucose or galactose conditions (*n* = 3 biologically independent experiments). Data with error bars are means ± SD. ***P  = 0.0002, *P  = 0.0255, ****P  = 5.74 × 10^−5^, from left to right. *P* values are determined using one-way ANOVA. (**G**) Flow cytometric analysis of JC-1 in Control or NDUFA3 KD mitotic MDA-MB-468 cells (*n* = 4 biologically independent experiments). Data with error bars are means ± SD. ****P  = 5.20 × 10^−5^, ***P  = 0.0001, from left to right. *P* values are determined using one-way ANOVA. Treatment with 100 μM Rotenone was used as a positive control. (**H**) Flow cytometric analysis of MitoSOX in Control or NDUFA3 KD mitotic MDA-MB-468 cells (*n* = 3 biologically independent experiments). Data with error bars are means ± SD. ****P  = 1.06 × 10^−5^, ***P  = 0.0001, from left to right. *P* values are determined using one-way ANOVA. Treatment with 100 μM Rotenone was used as a positive control.

To assess the function of mitotic supercomplexes, we first examined whether NDUFA3 depletion compromises mitochondrial function. BN-PAGE combined with in-gel Complex I (CI) activity assays showed a substantial decrease in NADH dehydrogenase activity in NDUFA3-knockdown HeLa and MDA-MB-468 mitotic cells (Figs. 4A and EV4A). Seahorse metabolic analysis further revealed significant reductions in both basal and maximal oxygen consumption rates (OCR) in these cells (Fig. 4B). Mitochondrial ATP production was markedly impaired in NDUFA3-depleted mitotic cells (Fig. 4B). Despite this, total cellular ATP levels remained unchanged (Figs. 4C and EV4B), suggesting the activation of compensatory pathways to maintain ATP availability. Indeed, extracellular acidification rate (ECAR) measurements indicated enhanced glycolysis in NDUFA3-knockdown mitotic cells (Fig. 4D), reflecting a metabolic rewiring from oxidative phosphorylation (OXPHOS) toward glycolysis as an adaptive response. Indeed, galactose culture forced OXPHOS dependence and induced ATP depletion in NDUFA3-KD HeLa cells (Fig. EV4C). By contrast, OXPHOS-dependent MDA-MB-468 cells already showed significant ATP depletion upon NDUFA3 depletion even in glucose medium (Fig. EV4D). This confirms supercomplex assembly is critical when mitochondrial ATP is limiting.

Using JC-1 staining, we observed mitochondrial membrane depolarization (ΔΨm) specifically in NDUFA3-knockdown cells during mitosis, but not in interphase cells (Fig. 4E). Notably, disruption of CI and supercomplex assembly specifically increased ROS during mitosis (Fig. 4F,G). Mitochondrial dysfunction was further exacerbated in MDA-MB-468 cells and upon galactose culture (Fig. EV4E–H). These cells rely more on OXPHOS, consistent with the critical role of supercomplex assembly when mitochondrial ATP is limiting. Additionally, cytosolic NADPH levels were reduced following NDUFA3 knockdown, as assessed using a genetically encoded NADPH sensor (Tao et al, 2017; Zou et al, 2018) (Fig. 4H). These results highlight a critical role for supercomplexes in maintaining ROS homeostasis during mitotic progression.

Because mitochondrial cristae structure is sensitive to redox changes (Norton et al, 2014; Willems et al, 2015), we assessed cristae integrity using transmission electron microscopy (TEM). NDUFA3 depletion specifically impaired cristae morphology in mitotic cells, resulting in fewer and shorter cristae with increased maximal width (Fig. 4I). These defects were rescued by NAC treatment but were not observed in interphase cells (Fig. 4I). Together, these data demonstrate that NDUFA3‑mediated supercomplex assembly is essential for attenuating ROS, preserving redox homeostasis, mitochondrial function, and cristae structure during mitosis.

### NDUFA3-mediated supercomplex assembly governs mitotic cell fate and ensures chromosome segregation fidelity in cancer cells

**Figure 5:**
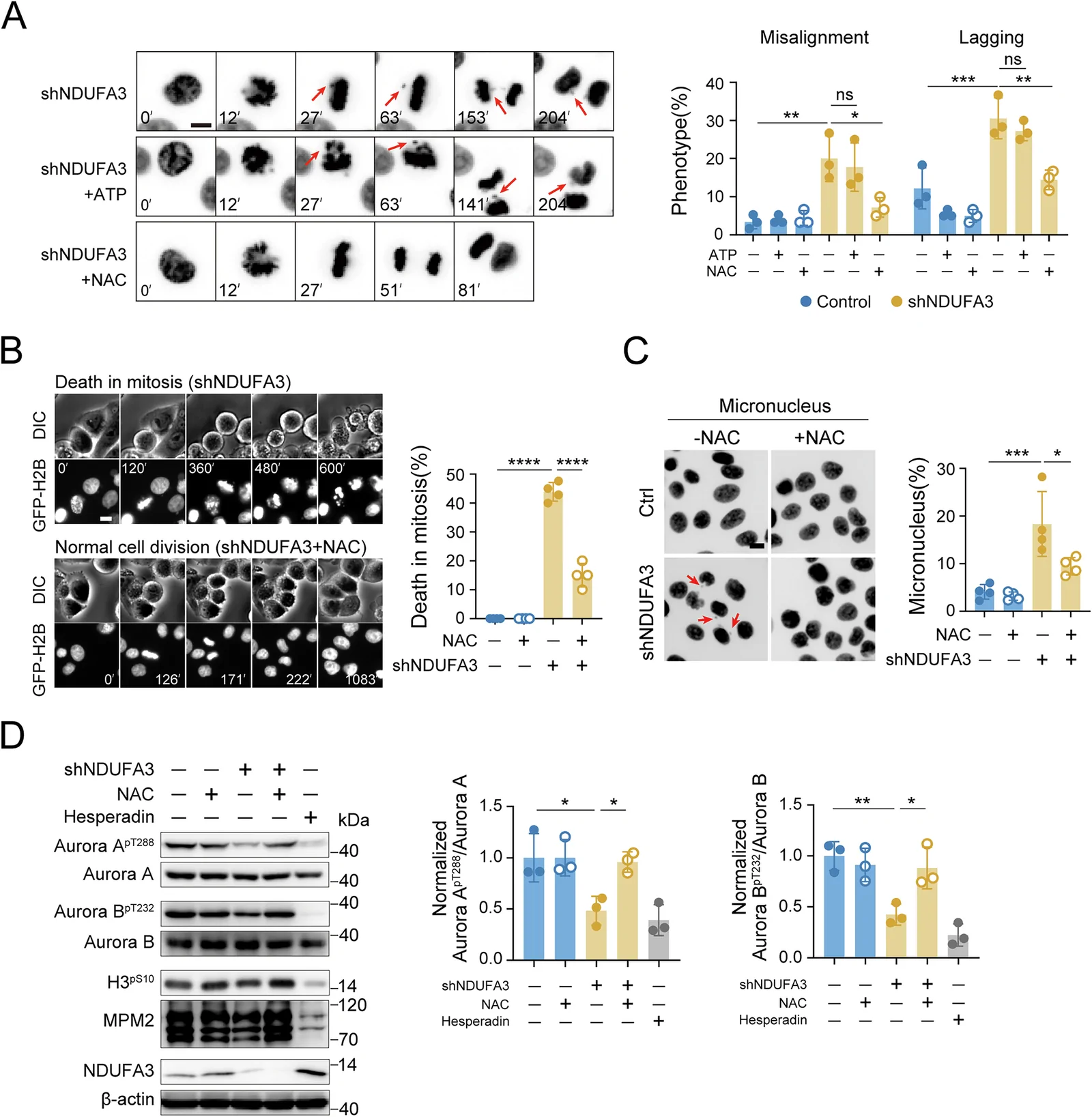
NDUFA3-mediated supercomplex assembly governs mitotic cell fate and ensures chromosome segregation fidelity in cancer cells. (**A**) Representative mitotic phenotypes of HeLa-GFP-H2B cells upon NDUFA3 KD treated with or without 1 mM ATP or 5 mM NAC were analyzed by time-lapse microscopy (left), in which the arrowheads indicate the misalignment and lagging chromosomes. Scale bar indicates 10 μm. Quantification of misalignment and lagging chromosomes in the mitotic progression was analyzed based on the images (*n* = 3 biologically independent experiments) (right). Data with error bars are means ± SD. **P  = 0.0023 (misalignment), (ns) *P*  = 0.9784 (misalignment), *P  = 0.0163 (misalignment), ***P  = 0.0004 (lagging), (ns) *P*  = 0.8461 (lagging), **P  = 0.0012 (lagging), from left to right. *P* values are determined using one-way ANOVA. (**B**) Representative mitotic cell death of HeLa-GFP-H2B cells upon Control or NDUFA3 KD following treatment with or without NAC were analyzed by time-lapse microscopy (left). Scale bars indicate 10 μm. Quantification of mitotic cell death was analyzed based on the images (*n* = 4 biologically independent experiments) (right). Data with error bars are means ± SD. ****P  = 7.75 × 10^−11^, ****P  = 1.23 × 10^−8^, from left to right. *P* values are determined using one-way ANOVA. (**C**) Representative micronucleus of HeLa-GFP-H2B cells upon Control or NDUFA3 KD following treatment with or without NAC were analyzed by time-lapse microscopy (left). Arrowheads point to micronucleus upon NDUFA3 KD. Scale bars indicate 10 μm. Quantification of micronucleus was analyzed based on the images (*n* = 4 biologically independent experiments) (right). Data with error bars are means ± SD. ***P  = 0.0006, *P  = 0.0215, from left to right. *P* values are determined using one-way ANOVA. (**D**) Western blot analysis of cell lysates from Control or NDUFA3 KD mitotic HeLa cells with the indicated antibodies (left). Quantified data are presented (*n* = 3 biologically independent experiments) (right). Data with error bars are means ± SD. *P  = 0.0231 (Aurora A^pT288^/Aurora A), *P  = 0.0365 (Aurora A^pT288^/Aurora A), **P  = 0.0057 (Aurora B^pT232^/Aurora B), *P  = 0.0241 (Aurora B^pT232^/Aurora B), from left to right. *P* values are determined using one-way ANOVA. Treatment with 100 nM Hesperadin was used as a positive control. Source data are available online for this figure.

**Figure EV5:**
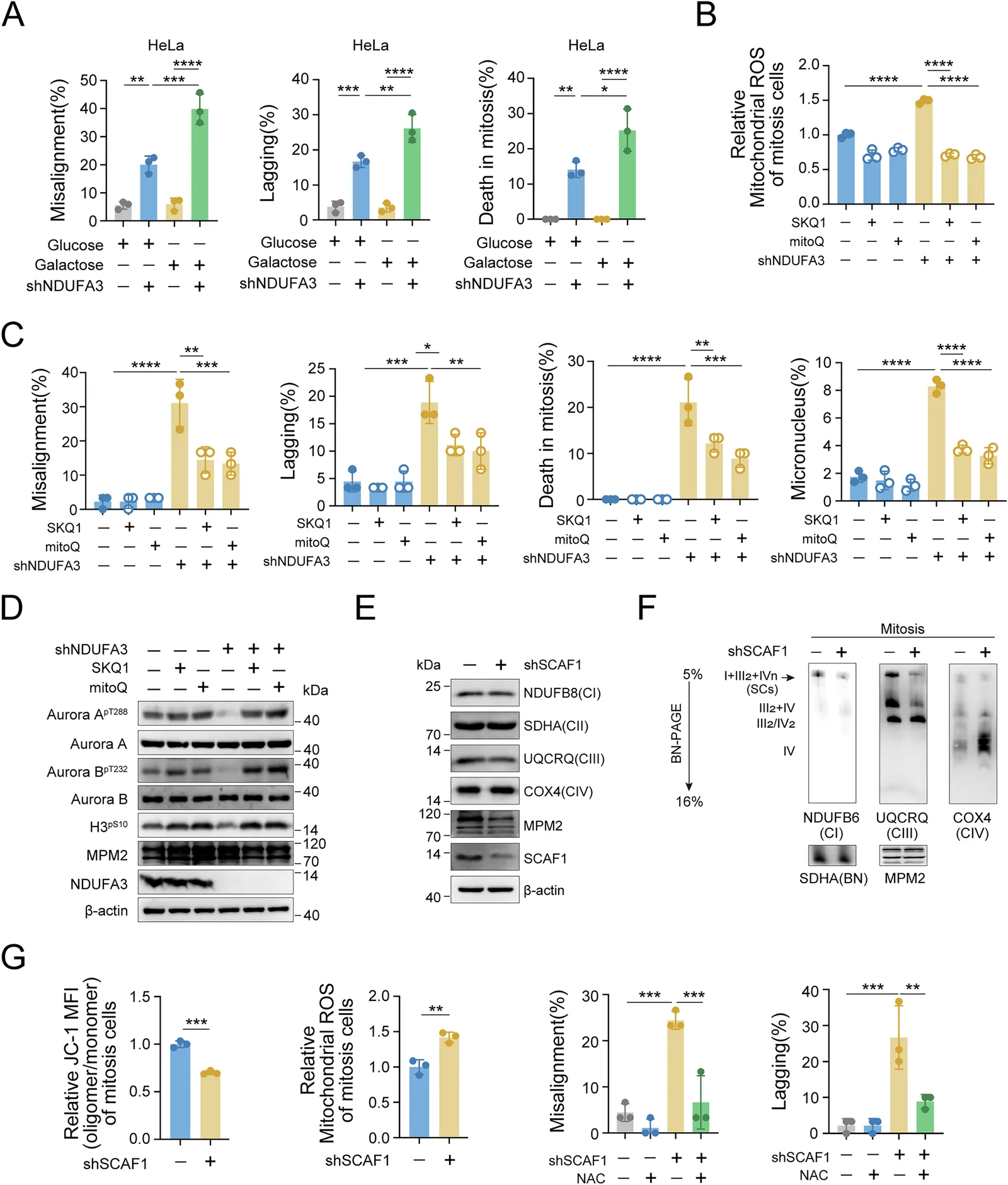
NDUFA3 depletion-induced mitotic defects are exacerbated by galactose, rescued by mitochondrial-targeted antioxidants, and recapitulated by SCAF1 knockdown. (**A**) Quantification of misalignment (left), lagging chromosomes (middle), and mitotic cell death (right) in Control or NDUFA3 KD HeLa cells, cultured under glucose or galactose conditions, were analyzed based on the live-cell images (*n* = 3 biologically independent experiments). Data with error bars are means ± SD. **P  = 0.0033 (misalignment), ***P  = 0.0004 (misalignment), ****P  = 8.15 × 10^−6^ (misalignment), ***P  = 0.0008 (lagging), **P  = 0.0056 (lagging), ****P  = 1.31 × 10^−5^ (lagging), **P  = 0.0029 (mitotic cell death), *P  = 0.0115 (mitotic cell death), ****P  = 5.05 × 10^−5^ (mitotic cell death), from left to right. *P* values are determined using one-way ANOVA. (**B**) Flow cytometric analysis of MitoSOX in Control or NDUFA3 KD mitotic HeLa cells, treated with or without SKQ1 or mitoQ (*n* = 3 biologically independent experiments). Data with error bars are means ± SD. ****P  = 9.38 × 10^−8^, ****P  = 4.59 × 10^−10^, ****P  = 2.57 × 10^−10^, from left to right. *P* values are determined using one-way ANOVA. (**C**) Quantification of misalignment (left), lagging chromosomes (middle-left), mitotic cell death (middle-right), and micronucleus (right) in Control or NDUFA3 KD HeLa cells, treated with or without SKQ1 or mitoQ, were analyzed based on the live-cell images (*n* = 3 biologically independent experiments). Data with error bars are means ± SD. ****P  = 6.51 × 10^−6^ (misalignment), **P  = 0.0014 (misalignment), ***P  = 0.0008 (misalignment), ***P  = 0.0001 (lagging), *P  = 0.0224 (lagging), **P  = 0.0089 (lagging), ****P  = 1.55 × 10^−6^ (mitotic cell death), **P  = 0.0060 (mitotic cell death), ***P  = 0.0004 (mitotic cell death), ****P  = 1.95 × 10^−8^ (micronucleus), ****P  = 1.20 × 10^−6^ (micronucleus), ****P  = 3.90 × 10^−7^ (micronucleus), from left to right. *P* values are determined using one-way ANOVA. (**D**) Western blot analysis of cell lysates from Control or NDUFA3 KD mitotic HeLa cells, treated with or without SKQ1 or mitoQ, with the indicated antibodies. (**E**) Western blot analysis of cell lysates from Control or SCAF1 KD mitotic HeLa cells with indicated proteins. (**F**) BN-PAGE immunoblots for the ETC complexes and supercomplexes of Control and SCAF1 KD mitotic HeLa cells, shown by levels of NDUFB6 (CI), UQCRQ (CIII) and COX4 (CIV). SDHA was used as a loading control. MPM2 was used as a mitosis marker. (**G**) Flow cytometric analysis of JC-1 (left) amd MitoSOX (middle-left) in Control or SCAF1 KD mitotic HeLa cells (*n* = 3 biologically independent experiments). Data with error bars are means ± SD. ***P  = 0.0002 (JC-1), **P  = 0.0045 (MitoSOX). *P* values are determined using unpaired two-tailed Student’s *t* test. Quantification of misalignment (middle-right) and lagging chromosomes (right) in Control or SCAF1 KD HeLa cells were analyzed based on the live-cell imaging (*n* = 3 biologically independent experiments). Data with error bars are means ± SD. ***P  = 0.0004 (misalignment), ***P  = 0.0008 (misalignment), ***P  = 0.0010 (lagging), **P  = 0.0074 (lagging), from left to right. *P* values are determined using one-way ANOVA.

To assess the functional importance of supercomplexes in mitotic progression, we disrupted its assembly via NDUFA3 knockdown and monitored chromosome dynamics by live‑cell imaging of GFP‑H2B (Fig. 5A). Depletion of NDUFA3 led to a significant increase in chromosome segregation defects, including misalignment and chromosome lagging (Fig. 5A). These mitotic defects were further exacerbated in galactose-cultured cells (Fig. EV5A). To test whether elevated mitotic ROS contributes to these defects, we treated cells with the antioxidant N‑acetylcysteine (NAC), which effectively scavenged intracellular ROS (Figs. 4F,G and 5A). Notably, NAC, but not exogenous ATP supplementation, rescued the mitotic defects induced by NDUFA3 knockdown (Fig. 5A). Live‑cell imaging further revealed that NDUFA3‑knockdown cells with severe alignment and segregation defects either underwent mitotic cell death (Fig. 5B) or exited mitosis into G1 with micronuclei—phenotypes that were rescued by NAC treatment (Fig. 5C). To further validate the causal role of ROS, we treated NDUFA3-KD cells with mitochondria-targeted scavengers SKQ1 and MitoQ. Both rescued ROS levels, and reduced mitotic defects (Fig. EV5B,C). Together, these data indicate that supercomplex assembly is required for accurate chromosome segregation in mitosis.

Because elevated ROS can oxidize cysteine residues near the catalytic sites of mitotic kinases such as Aurora A and Aurora B, thereby inhibiting their activity (Cheng et al, 2023; Cho et al, 2017; Lim et al, 2015), we examined the phosphorylation status of these kinases. Western blot analysis showed that NDUFA3 knockdown reduced the activation‑specific phosphorylation of both Aurora A and Aurora B. This reduction was restored by treatment with NAC, SKQ1, or mitoQ (Figs. 5D and EV5D). These findings suggest that ROS accumulation mechanistically links supercomplex disruption to impaired Aurora kinase activity, ultimately contributing to mitotic failure—a model further supported by earlier studies (Cheng et al, 2023; Cho et al, 2017; Lim et al, 2015).

**Figure EV6:**
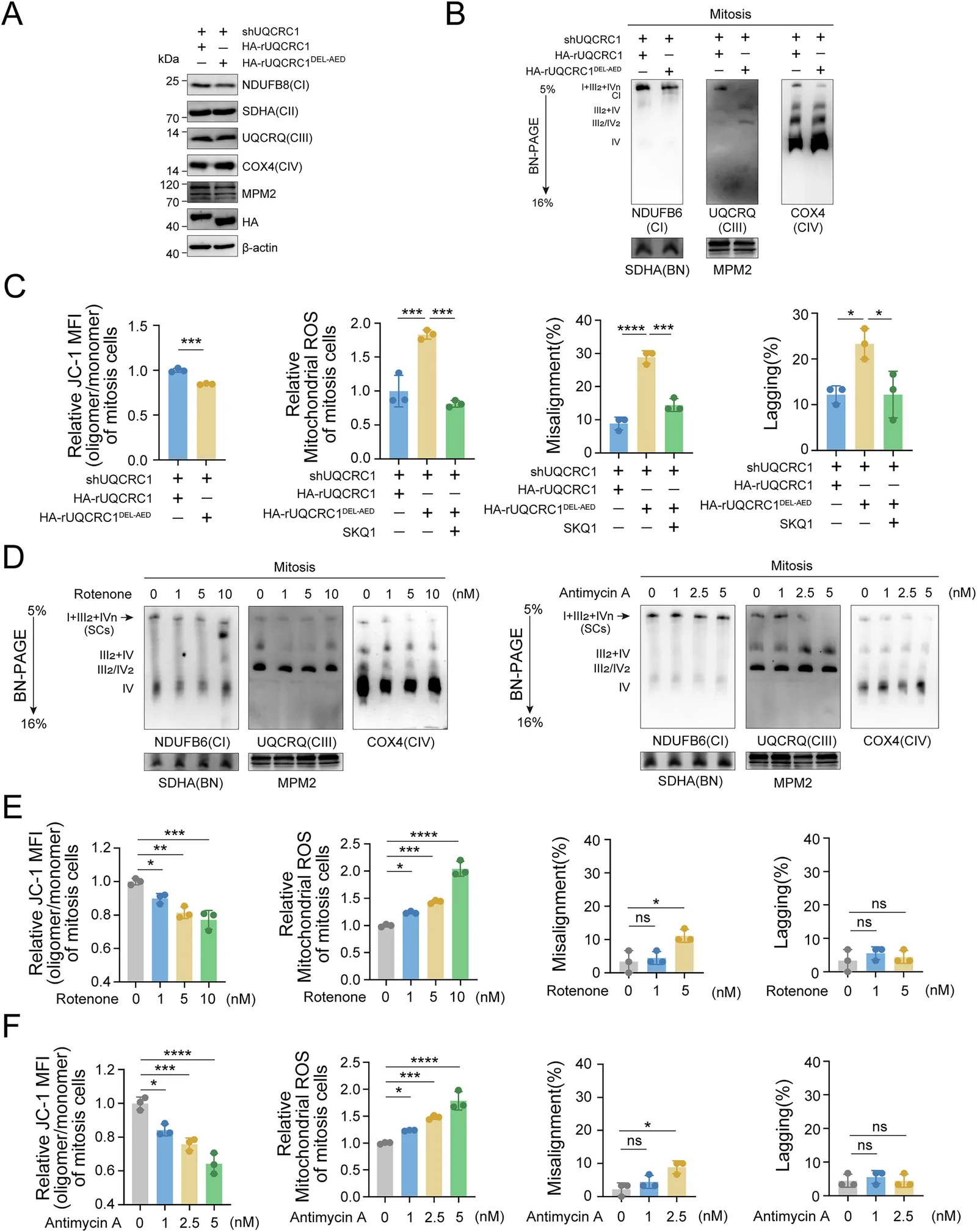
Disruption of CI-CIII₂ interaction via UQCRC1^DEL-AED^, but not catalytic inhibition of complex I or III, causes mitotic chromosome segregation defects. (**A**) Western blot analysis of the indicated proteins in lysates of UQCRC1 knockdown HeLa cells complemented with shRNA-resistant HA-UQCRC1^WT^ or HA-UQCRC1^DEL-AED^. (**B**) BN-PAGE immunoblots for the ETC complexes and supercomplexes of UQCRC1 knockdown HeLa cells complemented with shRNA-resistant HA-UQCRC1^WT^ or HA-UQCRC1^DEL-AED^, shown by levels of NDUFB6 (CI), UQCRQ (CIII) and COX4 (CIV). SDHA was used as a loading control. MPM2 was used as a mitosis marker. (**C**) Flow cytometric analysis of JC-1 (left) amd MitoSOX (middle-left) in UQCRC1 knockdown HeLa cells complemented with shRNA-resistant HA-UQCRC1^WT^ or HA-UQCRC1^DEL-AED^ (*n* = 3 biologically independent experiments). Data with error bars are means ± SD. ***P  = 0.0003 (JC-1). *P* values are determined using unpaired two-tailed Student’s *t* test (JC-1). ***P  = 0.0009 (MitoSOX), ***P  = 0.0003 (MitoSOX), from left to right. *P* values are determined using one-way ANOVA (MitoSOX). Quantification of misalignment (middle-right) and lagging chromosomes (right) in UQCRC1 knockdown HeLa cells complemented with shRNA-resistant HA-UQCRC1^WT^ or HA-UQCRC1^DEL-AED^ were analyzed based on the live-cell imaging (*n* = 3 biologically independent experiments). Data with error bars are means ± SD. ****P  = 3.55 × 10^−5^ (misalignment), ***P  = 0.0002 (misalignment), *P  = 0.0237 (lagging), *P  = 0.0237 (lagging), from left to right. *P* values are determined using one-way ANOVA. (**D**) BN-PAGE immunoblots for the ETC complexes and supercomplexes in mitotic HeLa cells treated with rotenone (left) or antimycin A (right), shown by levels of NDUFB6 (CI), UQCRQ (CIII) and COX4 (CIV). SDHA was used as a loading control. MPM2 was used as a mitosis marker. (**E**) Flow cytometric analysis of JC-1 (left) amd MitoSOX (middle-left) in mitotic HeLa cells treated with rotenone (*n* = 3 biologically independent experiments). Data with error bars are means ± SD. *P  = 0.0452 (JC-1), **P  = 0.0014 (JC-1), ***P  = 0.0003 (JC-1), *P  = 0.0166 (MitoSOX), ***P  = 0.0004 (MitoSOX), ****P  = 5.17 × 10^−7^ (MitoSOX), from left to right. *P* values are determined using one-way ANOVA. Quantification of misalignment (middle-right) and lagging chromosomes (right) in HeLa cells treated with rotenone were analyzed based on the live-cell images (*n* = 3 biologically independent experiments). Data with error bars are means ± SD. (ns) *P*  = 0.8514 (misalignment), *P  = 0.0202 (misalignment), (ns) *P*  = 0.5508 (lagging), (ns) *P*  = 0.8515 (lagging), from left to right. *P* values are determined using one-way ANOVA. (**F**) Flow cytometric analysis of JC-1 (left) amd MitoSOX (middle-left) in mitotic HeLa cells treated with antimycin A (*n* = 3 biologically independent experiments). Data with error bars are means ± SD. *P  = 0.0101 (JC-1), ***P  = 0.0007 (JC-1), ****P  = 4.19 × 10^−5^ (JC-1), *P  = 0.0457 (MitoSOX), ***P  = 0.0007 (MitoSOX), ****P  = 2.03 × 10^−5^ (MitoSOX), from left to right. *P* values are determined using one-way ANOVA. Quantification of misalignment (middle-right) and lagging chromosomes (right) in HeLa cells treated with antimycin A were analyzed based on the live-cell imaging (*n* = 3 biologically independent experiments). Data with error bars are means ± SD. (ns) *P*  = 0.3922 (misalignment), *P  = 0.0128 (misalignment), (ns) *P*  = 0.7684 (lagging), (ns) *P*  > 0.9999 (lagging), from left to right. *P* values are determined using one-way ANOVA.

To confirm that elevated ROS results specifically from supercomplex disassembly rather than general Complex I impairment, we disrupted supercomplex formation without directly targeting individual CI subunits by knocking down SCAF1 (Supercomplex Assembly Factor 1), which promotes the assembly of CIII₂ + CIV supercomplex and supercomplexes (Balsa et al, 2019; Fernandez-Vizarra et al, 2022; Ikeda et al, 2019; Lobo-Jarne et al, 2018; Vercellino and Sazanov, 2022; Yang et al, 2024). SCAF1 knockdown effectively impaired CIII₂ + CIV supercomplex and CI + CIII₂+CIVn supercomplex formation without affecting individual complex expression (Fig. EV5E, F). This increased ROS and caused membrane depolarization, with the resulting mitotic defects rescued by NAC (Fig. EV5G). To distinguish supercomplex-specific effects, we generated UQCRC1 knockdown cells complemented with shRNA-resistant HA-UQCRC1^DEL-AED^, a mutant that disrupts CI-CIII_2_ interaction (Liang et al, 2025; Milenkovic et al, 2023) while preserving individual complex expression (Fig. EV6A,B). These cells phenocopied the mitochondrial and mitotic defects of NDUFA3 knockdown, and the mitotic defects were rescued by SKQ1 treatment (Fig. EV6C). In contrast, rotenone or antimycin A treatment, which inhibit CI or CIII activity without disrupting supercomplex assembly, elevated ROS and reduced membrane potential but did not cause chromosome segregation defects (Fig. EV6D–F). These results confirm that supercomplex disassembly, rather than isolated CI dysfunction, elevates ROS and underlies the observed mitotic failures.

In summary, our results highlight a critical role for supercomplexes in safeguarding redox homeostasis during mitosis, thereby sustaining mitotic kinase activity, and ensuring accurate chromosome segregation.

### NDUFA3-mediated mitotic supercomplex assembly promotes tumor growth

**Figure 6:**
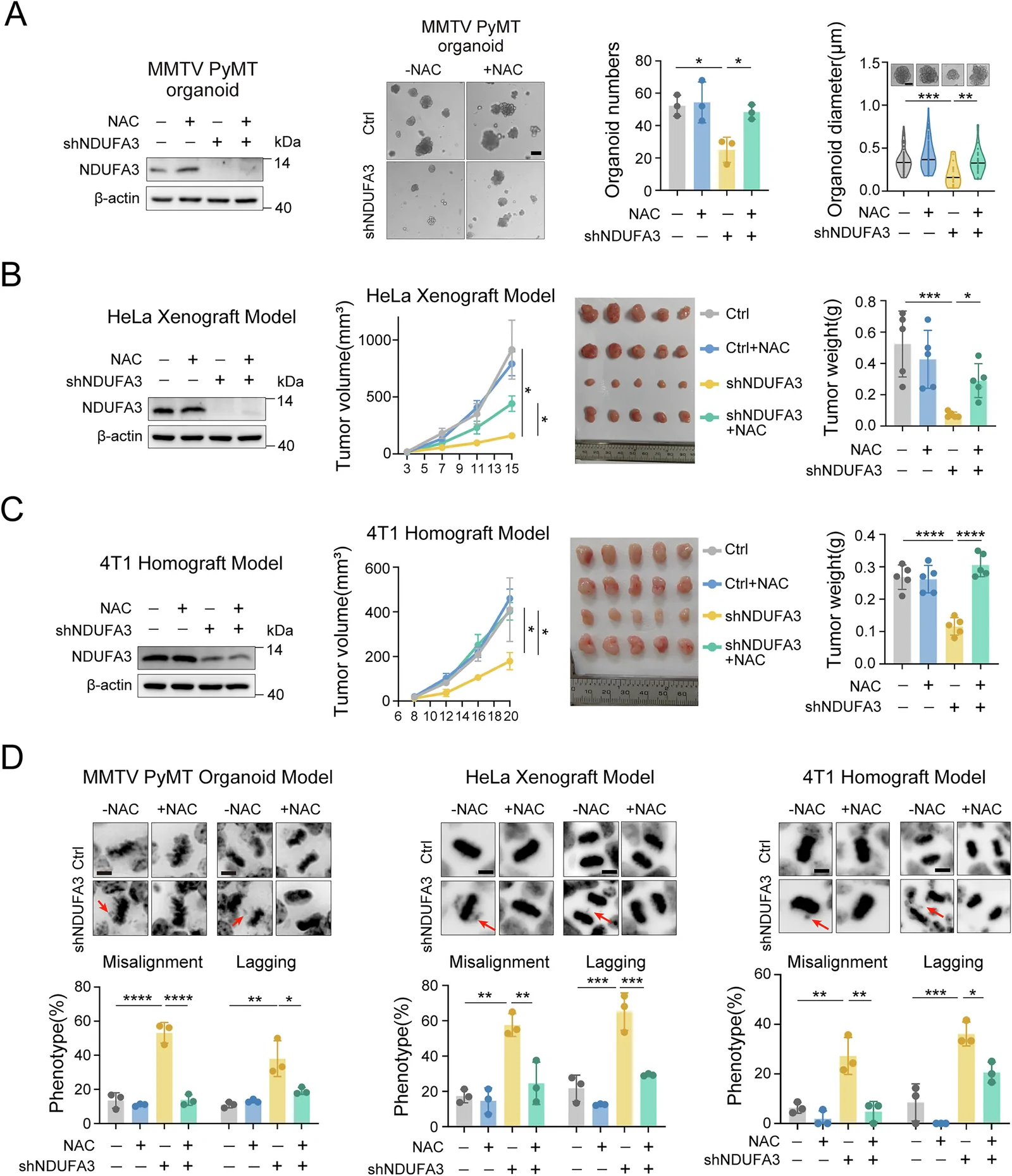
NDUFA3-mediated mitotic supercomplex assembly promotes tumor growth. (**A**) Western blot analysis of cell lysates from Control or NDUFA3 KD MMTV-PyMT organoids with indicated proteins (left). Representative images of the Control or NDUFA3 KD MMTV-PyMT organoids following treatment with or without NAC (middle-left). Quantification of organoid numbers was analyzed based on the images (*n* = 3 biologically independent experiments for each group) (middle- right). Data with error bars are means ± SD. *P  = 0.0173, *P  = 0.0384, from left to right. Quantification of organoid diameter (right) was analyzed based on the images (*n* = 94, 121, 18, 23 biologically independent organoids, from left to right) (right). Data is shown as violin plots. ***P  = 0.0003, **P  = 0.0091, from left to right. *P* values are determined using one-way ANOVA. Scale bars indicate 100 μm. (**B**) Western blot analysis of cell lysates from HeLa tumors upon indicated treatment with indicated proteins (left). Tumor volume of Control and NDUFA3 KD HeLa cells following treatment with or without NAC was measured at 3 d after inoculation (*n* = 5 biologically independent tumors for each group) (middle-left). Data points with error bars are means ± SD. *P  = 0.0140, *P  = 0.0447, from left to right. *P* values are determined using two‑way repeated measures ANOVA with Geisser–Greenhouse correction, followed by Tukey’s multiple comparisons test. Xenograft tumors were collected at the endpoint and shown (middle- right). Tumor weight at the endpoint was analyzed (*n* = 5 biologically independent tumors for each group) (right). Data with error bars are means ± SD. ***P  = 0.0008, *P  = 0.0487, from left to right. *P* values are determined using one-way ANOVA. (**C**) Western blot analysis of cell lysates from 4T1 tumors upon indicated treatment with indicated proteins (left). Tumor volume of Control and NDUFA3 KD 4T1 cells following treatment with or without NAC was measured at 8 d after inoculation (*n* = 5 biologically independent tumors for each group) (middle-left). Data points with error bars are means ± SD. *P  = 0.0432, *P  = 0.0466, from left to right. *P* values are determined using two‑way repeated measures ANOVA with Geisser–Greenhouse correction, followed by Tukey’s multiple comparisons test. Homograft tumors were collected at the endpoint and shown (middle-right). Tumor weight at the endpoint was analyzed (*n* = 5 biologically independent tumors for each group) (right). Data with error bars are means ± SD. ****P  = 3.29 × 10^−5^, ****P  = 2.03 × 10^−6^, from left to right. *P* values are determined using one-way ANOVA. (**D**) Frozen sections of three tumor models—MMTV-PyMT organoids (left), HeLa xenograft tumors (middle), and 4T1 homograft tumors (right)—were subjected to immunofluorescence staining with DAPI. Representative images of misalignment and lagging chromosome in the organoids and tumors upon Control or NDUFA3 KD following treatment with or without NAC (top). Arrowheads point to misalignment and lagging chromosomes in the organoids and tumors upon NDUFA3 KD. Scale bar indicates 10 μm. Quantification of misalignment and lagging chromosomes in the organoids and tumors was analyzed based on the images (*n* = 3 biologically independent organoids or tumors for each group) (bottom). Data with error bars are means ± SD. ****P  = 1.14 × 10^−5^ (misalignment, MMTV-PyMT organoids), ****P  = 1.20 × 10^−5^ (misalignment, MMTV-PyMT organoids), **P  = 0.0013 (lagging, MMTV-PyMT organoids), *P  = 0.0114 (lagging, MMTV-PyMT organoids), **P  = 0.0011 (misalignment, HeLa xenograft tumors), **P  = 0.0041 (misalignment, HeLa xenograft tumors), ***P  = 0.0002 (lagging, HeLa xenograft tumors), ***P  = 0.0007 (lagging, HeLa xenograft tumors), **P  = 0.0026 (misalignment, 4T1 homograft tumors), **P  = 0.0017 (misalignment, 4T1 homograft tumors), ***P  = 0.0006 (lagging, 4T1 homograft tumors), *P  = 0.0199 (lagging, 4T1 homograft tumors), from left to right. *P* values are determined using one-way ANOVA. Source data are available online for this figure.

To determine the contribution of mitotic supercomplex assembly to tumor progression, we first employed tumor organoids derived from MMTV-PyMT mice. NDUFA3 knockdown reduced both organoid number and size, phenotypes that were ameliorated by NAC treatment (Fig. 6A). We further evaluated tumor growth using xenograft and syngeneic models. Growth curves and endpoint tumor sizes demonstrated that NDUFA3 knockdown significantly suppressed the growth of HeLa (Fig. 6B) and 4T1 (Fig. 6C) tumors. This suppression was partially rescued when mice bearing NDUFA3‑knockdown tumors received NAC in their drinking water (Fig. 6B,C). Consistent with cellular phenotypes, NDUFA3‑deficient organoids and tumors exhibited increased frequencies of misaligned and lagging chromosomes during mitosis, which were again reduced upon NAC administration (Fig. 6D).

Collectively, these results indicate that loss of supercomplex integrity elevates mitotic ROS levels, thereby promoting chromosome mis‑segregation and ultimately impairing tumor progression in MMTV-PyMT-derived organoids, as well as HeLa and 4T1 xenograft models.

## Discussion

**Figure 7:**
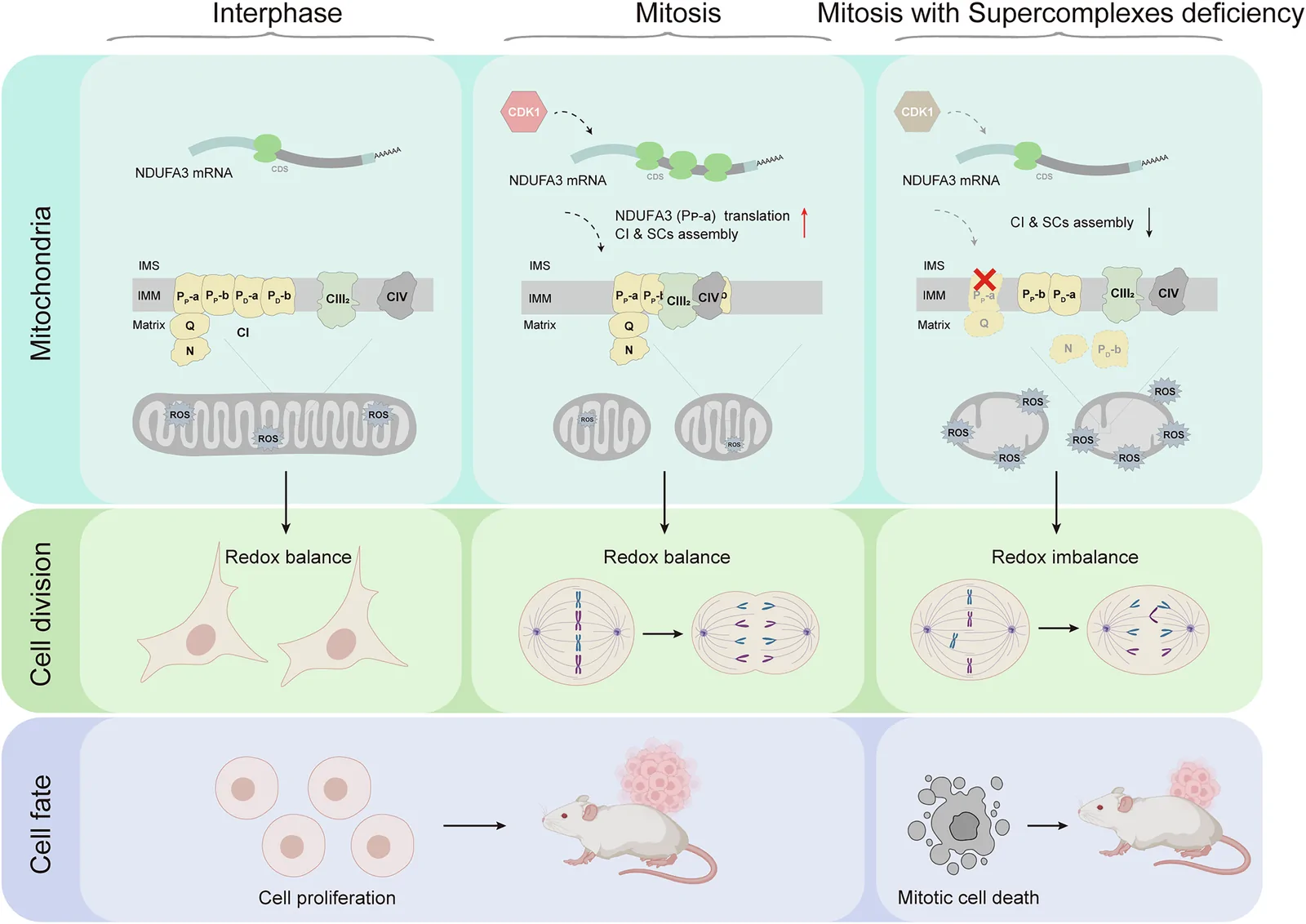
Schematic illustrating the underlying mechanism of NDUFA3-mediated mitotic supercomplex assembly in promoting tumor growth. Model for enhanced supercomplex assembly safeguards chromosome segregation by avoiding ROS overload during mitosis. Upon mitotic entry, activated CDK1 upregulates translation of the electron transport chain subunit NDUFA3, which promotes stepwise assembly of CI and respiratory supercomplexes. This sustains mitochondrial function and redox balance, permitting accurate chromosome segregation and cell proliferation. Supercomplex deficiency during mitosis disrupts cristae structure and impairs chromosome segregation due to ROS overproduction. In murine xenograft models, supercomplex deficiency-induced mitotic impairment and cell death are associated with suppressed tumor growth, underscoring the physiological relevance of this pathway in cancer cell division and tumor growth.

In this study, we demonstrate that despite mitochondrial fragmentation during mitosis, respiratory supercomplex formation is markedly upregulated. This process is driven by enhanced translation of specific ETC subunits, particularly those within the P_P_-a module of CI. We further establish that the supercomplexes is critical for maintaining redox homeostasis and cristae integrity following mitochondrial fission, which in turn is required for accurate chromosome segregation. Disruption of supercomplex assembly—via knockdown of NDUFA3—led to ROS overproduction and severe chromosome segregation errors. Our findings reveal a coordinated mechanism linking mitochondrial dynamics, inner membrane ETC organization, and metabolic adaptation during mitosis within these cancer cell models. Importantly, the suppression of tumor growth upon NDUFA3 knockdown in HeLa and 4T1 xenograft models, which was rescued by antioxidant treatment, underscores the critical role of ROS-mediated genomic instability in this process (Fig. 7).

We identify NDUFA3 as a key ETC component required for the early-stage assembly of the supercomplexes during mitosis. Supercomplex levels generally correlate with the abundance of individual respiratory complexes (Protasoni et al, 2020; Vercellino and Sazanov, 2022), and their alterations can reflect mitochondrial biogenesis in various physiological and pathological contexts (Greggio et al, 2017; Rodriguez-Nuevo et al, 2022; Wang et al, 2021). The mitotic mitochondrial proteome is highly plastic to meet cellular demands (Ly et al, 2017; Mahdessian et al, 2021; Tanenbaum et al, 2015). Similarly to NDUFA3, NDUFA8 and NDUFA13 also increase in mitosis, suggesting a broader demand for P_P_-a module subunits during cell division, although their functional roles in mitotic supercomplex assembly warrant future investigation. Supercomplex assembly is proposed to occur through intermediate steps, where pre-existing CIII₂ and CIV associate with incomplete CI modules (Protasoni et al, 2020). Recent study further indicates that the P_D_-a module of CI is sufficient to interact with CIII₂ and CIV to form the supercomplexes (Fang et al, 2021). Building on this, we propose that NDUFA3, likely as part of the P_P_-a module, hierarchically regulates the early-stage assembly of both CI and the supercomplexes during mitosis, thereby stabilizing individual respiratory complexes (Figs. 2A–E and EV2A–C). The high abundance of P_P_-a module subunits at assembly sites may facilitate CI and supercomplex formation, though the stoichiometric incorporation of these subunits into the final complex may not be strictly required. We acknowledge that the BN-PAGE resolution used here does not distinguish all possible supercomplex stoichiometries. Our data therefore primarily reflect changes in high-molecular-weight CI/CIII/CIV-containing assemblies. The precise mechanisms and stoichiometric relationships warrant further investigation.

Our data show that the mitotic kinase CDK1 is required for NDUFA3 expression at the translational level (Fig. 3). Mitochondrial metabolic adaptation involves multiple processes, including ETC proteome remodeling, protein import, cristae biogenesis, membrane dynamics, lipid remodeling, and supercomplex assembly (Bennett et al, 2022). CDK1 is known to regulate these processes by phosphorylating mitochondrial substrates such as TOM6 and CI subunits, thereby enhancing respiration (Harbauer et al, 2014; Wang et al, 2014; Xie et al, 2019) and activating MnSOD to alleviate oxidative stress (Candas et al, 2013; Xie et al, 2019) during mitosis. Here, we propose CDK1 as a key regulator of mitosis-dependent NDUFA3 translation, which promotes CI and supercomplex assembly to maintain redox balance. Ribosome profiling (Stumpf et al, 2013), combined with our ribosomal S6-IP and MG132 treatment data (Figs. 3E and EV3E), strongly suggests that NDUFA3 is translationally upregulated. The exact mechanism by which CDK1 regulates this translation remains unclear but may involve the phosphorylation of translation initiation factors (Imami et al, 2018; Kalous et al, 2020; Shuda et al, 2015) or specific structural features of the NDUFA3 mRNA (Ly et al, 2024; Novoa et al, 2010; Park et al, 2016; Sherrill et al, 2004; Shuda et al, 2015). We assessed CDK1-regulated translation factors (S6, 4EBP1) and confirmed their CDK1-dependent phosphorylation during mitosis (Fig. EV3F), supporting this regulatory model. Thus, CDK1 appears to support mitochondrial proteome stability during mitosis by upregulating both NDUFA3 translation and mitochondrial protein import (Harbauer et al, 2014), though the detailed pathways require further elucidation.

Mitotic cells exhibited elevated oxygen consumption rate and ATP levels compared to interphase cells (Fig. 4B,C). While these findings are consistent with an increase in respiratory capacity during mitosis, it remains possible that the changes in supercomplex assembly involve additional or alternative mechanisms. Previous studies have shown that reduced respiratory capacity can also promote supercomplex assembly under certain conditions (Balsa et al, 2019; Greggio et al, 2017; Hollinshead et al, 2020; Lopez-Fabuel et al, 2016). Given the complex and context‑dependent relationship between metabolic state and supercomplex assembly, our data are compatible with a model in which enhanced expression of Complex I subunits drives supercomplex assembly during mitosis. Nevertheless, we acknowledge that the observed metabolic changes may also represent a concurrent phenomenon rather than a distinct primary cause.

Although NDUFA3 knockdown impaired the assembly of the supercomplexes, it did not significantly alter overall cellular energy metabolism under high-glucose conditions (Fig. EV4B,C). This lack of a pronounced metabolic phenotype may be attributed to glycolytic compensation (Balsa et al, 2019; Wang et al, 2021), as evidenced by increased ECAR in NDUFA3-depleted HeLa cells (Fig. 4D). The inherent reliance on glycolysis in HeLa cells likely masks the respiratory deficiency. By contrast, in OXPHOS-dependent models (Chen et al, 2023; Hollinshead et al, 2020; Ikeda et al, 2019; Yang et al, 2024) such as MDA-MB-468 cells, NDUFA3 knockdown reduced ATP production and exacerbated mitochondrial functional defects (Fig. EV4D,G,H). Consistently, culturing NDUFA3-KD HeLa cells in galactose medium to enforce OXPHOS dependency also significantly exacerbated mitochondrial and mitotic defects (Fig. EV4C,E,F). Therefore, energy deficiency amplifies mitotic defects specifically under OXPHOS-dependent conditions, highlighting the importance of supercomplex assembly under such conditions. We acknowledge the preliminary nature of this study, including limited sample sizes. The partial NAC rescue effect in vivo is suggestive and requires validation in larger cohorts and additional models. We also acknowledge that the mitotic increase in respiratory supercomplex assembly was substantially weaker in non-transformed RPE cells than in cancer cell lines. This observation raises the possibility that the pathway we describe may be particularly relevant in cancer cells—which often operate under elevated oxidative and mitotic stress—rather than representing a universal requirement for cell division. Further studies are needed to determine whether mitotic supercomplex remodeling is a general feature or specific to certain cancer types.

In conclusion, our work highlights the multifaceted roles of supercomplex assembly in mitochondrial adaptation during mitosis, and points to the therapeutic potential of targeting supercomplex assembly factors—particularly in combination with mitotic arrest agents—in these cancer models.

## Methods

**Table:** Reagents and tools table

Reagent/resource	Reference or source	Identifier or catalog number
**Experimental models**		
BALB/C nude mouse	Shanghai SLAC Laboratory Animal Company	N/A
BALB/C mouse	Shanghai SLAC Laboratory Animal Company	N/A
MMTV-PyMT mouse	Jackson Laboratory	N/A
HEK293T	ATCC	CRL-3216
HeLa	CAS, Shanghai	TCHu187
MDA-MB-468	ATCC	HTB-132
4T1	ATCC	CRL-2539
T47D	CAS, Shanghai	TCHu87
MCF-7	ATCC	HTB-22
RPE	ATCC	CRL-4000
**Recombinant DNA**		
NDUFA3-3xHA	This manuscript	N/A
Clover-ScFv	This manuscript	N/A
HA-rUQCRC1^WT/DEL-AED^	This manuscript	N/A
iNap1	Tao et al, 2017	N/A
ATeam1.03	Addgene	Cat# 28003
**Antibodies**		
β-actin	Proteintech	Cat# 60008-1-Ig
NDUFA3	GeneTex	Cat# GTX56108
NDUFA3	Proteintech	Cat# 17257-1-AP
NDUFA8	Proteintech	Cat# 15064-1-AP
NDUFA13(GRIM19)	Proteintech	Cat# 10986-1-AP
NDUFV2	Proteintech	Cat# 15301-1-AP
NDUFS1	Proteintech	Cat# 12444-1-AP
NDUFS3	Proteintech	Cat# 15066-1-AP
NDUFA9	Proteintech	Cat# 20312-1-AP
NDUFC2	Proteintech	Cat# 15573-1-AP
NDUFA10	Proteintech	Cat# 32947-1-AP
NDUFB6	Proteintech	Cat# 16037-1-AP
NDUFB11	Proteintech	Cat# 16720-1-AP
NDUFB8	Proteintech	Cat# 14794-1-AP
SDHA	Proteintech	Cat# 14865-1-AP
UQCRSF1	Proteintech	Cat# 18443-1-AP
UQCRQ	Proteintech	Cat# 14975-1-AP
COX4	Proteintech	Cat# 11242-1-AP
SCAF1/COX7A2L	Proteintech	Cat# 11416-1-AP
Tom20	Proteintech	Cat# 11802-1-AP
Tim23	Proteintech	Cat# 11123-1-AP
α-tubulin	Proteintech	Cat# 11224-1-AP
Aurora A	BD biosciences	Cat# BD610939
Aurora B	BD biosciences	Cat# 611082
pAurora A/B/C	CST	Cat# 2914
CCNB1	Proteintech	Cat# 55004-1-AP
phosphor-H3S10	Millipore	Cat# 06-570
MPM2	Millipore	Cat# 2669855
HA	Proteintech	Cat# 51064-2-AP
4EBP1	CST	Cat# 9644
p4EBP1(Thr37/46)	CST	Cat# 2855
eIF2α	CST	Cat# 5324
peIF2α(Ser51)	CST	Cat# 3398
S6	Santa Cruz	Cat# sc-74459
pS6(Ser235/236)	CST	Cat# 62016
HRP-conjugated anti-rabbit secondary antibody	Jackson ImmunoResearch Labs	Cat# 111-035-003
HRP-conjugated anti-mouse secondary antibody	Jackson ImmunoResearch Labs	Cat# 115-035-003
Alexa 594-conjugated anti-rabbit secondary antibody	Jackson ImmunoResearch Labs	Cat# 111-585-144
Alexa 488-conjugated anti-mouse secondary antibody	Jackson ImmunoResearch Labs	Cat# 115-545-146
Alexa 594-conjugated anti-mouse secondary antibody	Jackson ImmunoResearch Labs	Cat# 115-585-146
**Oligonucleotides and other sequence-based reagents**		
Human-NDUFA3-shRNA	CTACTTCAAGTACTCCGTCAT	N/A
Mouse-NDUFA3-shRNA	TGTGAGAGATGACGGGAACAT	N/A
Human-SCAF1-shRNA	CCGATTCCACAGTGTATGATT	N/A
Human-UQCRC1-shRNA	GCACACAGCTTACTACATCAA	N/A
qPCR-Human-NDUFA3-F	GGGGCCTCGCTGTAATTCTG	N/A
qPCR-Human-NDUFA3-R	GACGGGCACTGGGTAGTTG	N/A
qPCR-Human-CCNB1-F	GCACTTTCCTCCTTCTCA	N/A
qPCR-Human-CCNB1-R	CGATGTGGCATACTTGTT	N/A
NDUFA3-F	ATGGCTGCGAGAGTCGGC	N/A
NDUFA3-R	CAGTTTCTTCAGCCACT	N/A
**Chemicals, enzymes and other reagents**		
Thymidine	Sigma	Cat# T1895
RO3306	Selleck	Cat# S7747
Rapamycin	Selleck	Cat# S1039
MG132	Selleck	Cat# S2619
2DG	Sigma	Cat# D8375
Oligomycin A	Selleck	Cat# S1478
Glucose	Sigma	Cat# 7021
Galactose	Beyotime	Cat# T1218
ATP	Sigma	Cat# A2383
Rotenone	Sigma	Cat# R8875
Antimycin A	GLPBIO	Cat# GC49360
Hesperadin	Selleck	Cat# S1529
Trizol	SuperfecTRI	Cat# 3101-100
Puromycin	Sigma	Cat# P8833
G418	Sangon Biotech	Cat# A600958-0001
DAPI	Sigma	Cat# D9542
DMSO	Sangon Biotech	Cat# A600163
DAKO	Dako	Cat# S302380-2
Polyethylenimine	Polysciences	Cat# 23966
JC-1	Sigma	Cat# T4069
MitoSox	Thermo	Cat# M36008
CM-H2DCFDA	Thermo	Cat# C6827
NAC	Sigma	Cat# A9165
SKQ1	Selleck	Cat# S9729
mitoQ	MedChemExpress	Cat# HY-100116A
Tissue-Tek® O.C.T. Compound	Sakura Finetek	Cat# 25608-930
DHEA	Selleck	Cat# S2604
Aminocaproic acid	Sigma	Cat# A2504
Bis-tris	Sigma	Cat# B7535
Coomassie blue G250 (Serva Blue G)	Serva	Cat# 35050
Digitonin	Sigma	Cat# D141
Tricine	Sigma	Cat# T9784
Nitrotetrazolium Blue chloride(NTB)	Sangon Biotech	Cat# A610379-0001
Glutamax 100×	Thermo	Cat# 35050061
HEPES	Gibco	Cat# 15630106
penicillin/streptomycin	Beyotime	Cat# C0222
B27	Thermo	Cat# 17504044
EGF (murine)	Thermo	Cat# PMG8045
Noggin (murine)	Peprotech	Cat# 120-10 C
R-spondin 1 (murine)	Peprotech	Cat# 120-38
TrypLE	Thermo	Cat# 12604013
Matrigel	Corning	Cat# 354234
Matrigel	Corning	Cat# 356231
**Software**		
ImageJ	NIH	https://imagej.nih.gov/
GraphPad Prism 8.0	Graphpad	https://www.graphpad.com/
FlowJo_v10.6.2	FlowJo	https://www.flowjo.com/
**Other**		
ATP Assay Kit	Beyotime	Cat# S0026
Seahorse XF Cell Mito Stress Test Kit	Agilent	Cat# 103015-100
DMEM Medium	Gibco	Cat# 12800017
DMEM medium	Gibco	Cat# A1443001
Leibovitz L-15 medium	Gibco	Cat# 21083027
Advanced DMEM/F12	Gibco	Cat# 12634010
µ-Slide 8 Well	ibidi	Cat# 80826
Glass Bottom Cell Culture dish	NEST	Cat# 801001
Four-chamber glass bottom dishes	Cellvis	Cat# D35C4-20-1-N
Nunclon delta surface tissue culture dish 24 wells	Thermo	Cat# 142475

### Experimental model and subject details

#### Animals

Animals were housed within a specific pathogen-free facility, maintained at a temperature range of 20–22 °C. The environment was regulated to ensure a 12-h light and 12-h dark cycle, with relative humidity levels kept between 50% and 60%. All animals had free access to food and water throughout the study. BALB/c and BALB/c Nude mice strains were purchased from Shanghai SLAC Laboratory Animal. FVB MMTV-PyMT mice were purchased from The Jackson Laboratory. Female mice at 6–8 weeks old were used for experiments maintained under specific pathogen-free conditions. The mice were randomly divided into different groups (*n* = 5 per group). All animal experiments were performed according to the approval of the Animal Research Ethics Committee of the University of Science and Technology of China: 2023-N (A)-393.

#### Cell culture

HeLa, MDA-MB-468, MCF-7 and HEK293T cell lines were maintained in Dulbecco’s modified Eagle’s medium (DMEM) (Gibco) supplemented with 5% fetal bovine serum and 5% newborn calf serum, 1% penicillin and streptomycin. T47D and 4T1 cell lines were maintained in RPMI-1640 medium (Gibco) supplemented with 10% fetal bovine serum (Gibco, Thermo Scientific) and 1% penicillin and streptomycin. All cells were cultured in a 5% CO_2_ humidified incubator at 37 °C.

#### Generation of xenograft and homograft model

For xenograft experiments, 5 × 10^6^ HeLa cells were injected subcutaneously into the left or right flank of BALB/c nude mice. For homograft experiments, 1 × 10^5^ 4T1 cells were inoculated subcutaneously into the fourth and ninth mammary glands of BALB/c mice. Tumor volumes were assessed every 2 days and calculated as 0.5 ×  length × width × width. The mice were treated with NAC (4 mg/kg, every 2 days), Doxycycline (1 mg/ml, every 2 days), and Sucrose (20 mg/ml, every 2 days) after injection until sacrificed the mice. The tumor burden was less than the maximum tumor size (1 cm^3^). The NAC dose was selected based on protocols established in our previous work (Cheng et al, 2023), which demonstrated effective antioxidant activity in murine xenograft models.

#### Generation of mouse-derived organoid

Primary mouse breast cancer tumor tissues were from the MMTV-PyMT mice and were established into the MMTV-PyMT organoids models as previously described. In detail, the fresh mouse breast cancer tissues were minced and digested with collagenase on an orbital shaker for 2 h at 37 °C. The digested tissue suspension was strained over a 100 mm filter, centrifugated at 400 rcf and then the red blood cell lysis buffer was added for 5 min. The pellet was resuspended in 20 μl PBS followed by mixing with 20 μl growth-factor-reduced Matrigel (Corning, cat# 356231), and the drops of Matrigel-cell suspension were added on prewarmed 24-well culture plates to solidify for 30 min at 37 °C before covering with the organoid culture medium (Dekkers et al, 2021; Duarte et al, 2017).

#### Plasmids and cell transfection

For lentiviral shRNA constructs, the annealed oligonucleotides were inserted into pLKO.1. All the target sequences for shRNA were listed in the Reagents and Tools Table. To knock down NDUFA3, SCAF1 in cells, the plasmid encoding the targeted shRNA or a scrambled control was transfected into 293 T cells. For lentiviral production, 293T cells were transfected with a lentiviral vector plus two packaging plasmids, psPAX2 and pMD2.G mixed with Polyethylenimine (PEI). Lentiviral supernatant was collected through filtered using a 0.45-μm syringe filter 48 h after transfection. For lentivirus infection, cells were supplemented with viral medium containing 8 μg/ml Polybrene (Millipore) for 24 h. Subsequently, transfected cells were selected with puromycin (2 μg/ml) or G418 (500 μg/ml). After selection, the transfected cells were analyzed 48–96 h after transfection or maintained with 1 mg/ml puromycin and subjected to further analysis.

For lentivirus infection, organoids were dissociated into single cells with TrypLE (Thermo, 12604013) at 37 °C for 10 min. Cells were pelleted at 1200 rpm for 5 min, resuspended in virus suspension supplemented with 8 μg/ml Polybrene (Millipore) at 37 °C for 5–7 h. After the drops of Matrigel-cell suspension were added on prewarmed 24-well culture plates, the medium was supplemented with 2 μg/ml puromycin as previously described (Duarte et al, 2017).

#### Cell synchronization

For interphase (G1/S) synchronization, cancer cells were incubated in the medium containing 2 mM thymidine for 20 h and then collected for experiments. For mitotic synchronization, HeLa or breast cancer cells were incubated in the medium containing 2 mM thymidine or 10 μM RO3306 for 19 h, and released into fresh medium for 9 h or 1 h. Then, mitotic cells were collected by shaking off and centrifugation. To maintain mitotic arrest during downstream assays, mitotic cells were replated in medium containing 10 μM MG132.

#### Immunofluorescence analysis

Cells were seeded on glass slides. The next day, cells were permeabilized with 0.2% Triton X-100 for 10–15 s. Next, the cells were fixed with 4% paraformaldehyde for 15 min at room temperature and then blocked with 1% BSA for 1 h. Cells were incubated with the primary antibodies for 2 h at room temperature followed by secondary antibodies incubation and DAPI staining. Frozen sections were permeabilized with 0.2% Triton X-100 for 15 min and then blocked with 1% BSA for 2 h. DAPI Nuclei were visualized by DAPI staining. Finally, the cell slides or frozen sections were mounted with DAKO Fluorescence Mounting Medium (Dako North America, S302380-2). Images were acquired using a DeltaVision Deconvolution microscope (GE Healthcare, Buckinghamshire, UK).

#### Time-lapse imaging of mitotic progression

HeLa or MDA-MB-468/GFP-H2B stable cell lines were seeded on an eight-chambered cover glass (Ibidi, 80826). Images were collected every 3 min using ×20 lens objective with Nikon Eclipse Ti-E NIS-Elements AR software (version 4.30.1.10210). The temperature of the imaging medium was kept at 37 °C. Image sequences were viewed and analyzed with ImageJ software.

Plvx-NDUFA3-3xHA-G418 was transfected into HEK293T cells along with psPAX2 and pMD2.G. 48 h later, HeLa-Clover-ScFv stable cell lines were transduced with lentivirus containing sequences for NDUFA3-3xHA. Monoclonal cell populations were isolated and selected for use in the subsequent experiments. Next, cells were plated on a 35-mm glass-bottom dish with DMEM medium. Images were collected every 5 min using ×40 lens objective with DeltaVision softWoRx software (version 6.5.2). The temperature of the imaging medium was kept at 37 °C. Image sequences were viewed and analyzed with ImageJ software. *F*_*Min*_ is calculated as the minimal background-subtracted fluorescence intensity. *F* indicates the background-subtracted fluorescence intensity at each time point. *F/F*_*Min*_ indicates the NDUFA3-3xHA concentration change during the cell cycle.

#### Live-cell imaging of NADPH biosensor

Plasmid for the NADPH biosensor (iNap1, a gift from Y. Yang and Y. Zhao at East China University of Science and Technology) was respectively transfected into HEK293T cells along with psPAX2 and pMD2.G. Forty-eight hours later, HeLa cells were transduced with lentivirus containing sequences for the NADPH biosensor. Next, cells were plated on a 35-mm glass-bottom dish with DMEM medium. Images were acquired with DeltaVision softWoRx software (version 6.5.2). For dual-excitation ratio imaging, cells were excited at 425 nm or 485 nm and measured at 525 nm. Images were analyzed with ImageJ software (Zou et al, 2018).

#### ATP measurement

ATP was measured using a luciferase-based luminescence kit (Beyotime) and the assay was performed according to the manufacturer’s instructions. After centrifugation to remove cell debris, the supernatant was added to the substrate solution. The luminescence was recorded in an Illuminometer with an integration time of 10 s per well. The ATP levels were determined using a standard curve for the luciferase assay and then normalized to the loading control of β-actin.

For ATeam1.03 imaging, 438/24 nm excitation filter and two emission filters (474/24 nm for cyan fluorescent protein (CFP) and 548/22 nm for yellow fluorescent protein (YFP)) were used. Exposure time was recorded at the indicated time points. Images were acquired with DeltaVision softWoRx software (version 6.5.2). Imaging data were analyzed with ImageJ software, and fluorescence ratios were normalized to the control condition values.

#### Measurement of ROS production

Cells were incubated in 1 μM CM-H_2_DCFDA (Thermo, C6827), 2.5 μM MitoSOX (Thermo, M36008) for 30 min and then washed away the excess probes (Murphy et al, 2022). Cells were analyzed by flow cytometers (BD Bioscience) or imaged in 35-mm glass-bottom MatTek dishes in PBS following the manufacturer’s instructions. Imaging data were analyzed with ImageJ software.

#### Mitochondrial membrane potential probe

Cells were incubated in 2.5 μg/ml JC-1 (Sigma, T4069) for 30 min and washed away the excess probes. Cells were analyzed by flow cytometers (BD Bioscience) or imaged in 35-mm glass-bottom MatTek dishes. The emission of JC-1 monomers was detected in Fl-1 using a 530/30 nm bandpass filter, and JC-1 oligomers were detected in Fl-2 using a 585/42 nm bandpass filter. Imaging data were analyzed with ImageJ software. Statistics were calculated according to the ratio of JC-1 oligomers to monomers.

#### Mitochondrial respiration assay

In total, 4 × 10^4^ mitotic cells were shaken off after synchronization and washed twice with XF base medium (Agilent) supplemented with 10 mM glucose, 1 mM pyruvate and 2 mM glutamine for OCR or 1 mM pyruvate and 2 mM glutamine for EACR. The mitotic cells were flung onto a Seahorse XF96 plate (Agilent) coated with 0.001% (w/v) poly-L-lysine (Sigma) for improved cell adherence. Cells were then incubated in a non-CO_2_ incubator at 37 °C for 1 h. For the Mito Stress Test, OCR values after the addition of 15 μM oligomycin, 10 μM FCCP, or 5 μM rotenone/antimycin A. For the Glycolysis Stress Test, ECAR values were obtained after the addition of 10 mM glucose, 10 μM oligomycin or 500 mM 2-deoxyglucose. OCR and ECAR were recorded using the XFe96 extracellular flux analyzer (Agilent).

#### Western blot

Cells or mitochondrial fractions were lysed in the cell lysis buffer (50 mM Tris-HCl pH 7.4, 150 mM NaCl, 1% Triton X-100, 1 mM EDTA, 1 mM Na_2_P_2_O_4_, 1 mM Na_3_VO_4_ and 50 mM NaF and protease inhibitors cocktail) and incubated on ice for 20 min. After centrifugation at 4 °C, the supernatant was transferred to the new tubes and protein concentrations were measured using the Bradford method. For immunoblotting, the lysates were boiled at 95 °C for 5 min and separated on 10%–15% SDS–PAGE followed by transfer to 0.45 or 0.2-μm PVDF membranes (Millipore). Membranes were blocked with 5% non-fat milk in TBST for 1 h and incubated with primary antibodies overnight at 4 °C and secondary antibodies for 1 h at room temperature. Images were acquired with Western Lightning Chemiluminescence Reagent Plus ChemiScope software (version 17.05.17 A). Quantification was performed using ImageJ. β-actin, or Tubulin was used as a loading control for the whole-cell samples; TOM20 was used as a loading control for the purified mitochondrial samples. Detailed information of antibodies is listed in the Reagent_Table.

#### qPCR

Total RNA was extracted using RNAiso plus (Takara), and the PrimeScript RT Master mix kit (Takara) was used for the reverse transcription reaction. Quantitative RT-PCR (RT-qPCR) was performed using an Applied Biosystems 7500HT sequence detection system with a FastStart Universal SYBR Green Master Mix kit (Monad). β-actin served as an internal control. Primer sequences are listed in the Reagent_Table.

#### Ribosomal protein S6 immunoprecipitation assay

Cell lysates for S6-IP were prepared using RNA immunoprecipitation buffer (10 mM Hepes, pH 8.0, 100 mM KCl, 5 mM MgCl₂, 1% Triton X-100, 0.5% sodium deoxycholate, 100 U/ml RNase inhibitor, and 100 μg/ml cycloheximide). The lysates were subjected to immunoprecipitation with an anti-ribosomal protein S6 antibody (Santa Cruz) for 2 h, followed by incubation with protein A beads for an additional hour at 4 °C. After three washes with the RNA-IP buffer, RNA was extracted from the S6-IP beads using Trizol (Invitrogen). The mRNAs associated with S6 were subsequently analyzed by RT‑qPCR.

#### Cytosolic and mitochondrial fractionation

Cells were collected and resuspended in mitochondrial isolation buffer (225 mM mannitol, 75 mM sucrose, 30 mM Tris-HCl (pH 7.4), 0.1 mM EGTA) supplemented with protease inhibitor cocktail, and subsequently homogenized using a prechilled Dounce homogenizer (Wieckowski et al, 2009). The homogenate was initially centrifuged for 10 min at 700 × *g* at 4 °C to remove unbroken cells and nuclei. The resulting supernatant was centrifuged for 10 min at 8000 × *g* at 4 °C. The supernatants were saved as cytosolic fractions, whereas the mitochondrial pellets were washed twice and stored in the mitochondrial buffer.

#### BN-PAGE

BN-PAGE was performed according to a published protocol (Wittig et al, 2006). Briefly, cells were harvested and resuspended in cold PBS supplemented with 2 mg/ml digitonin (Sigma, D141) and protease inhibitors cocktail. Next, the solution was incubated on ice for 10 min and centrifugated at 10,000 × *g* for 10 min at 4 °C. The mitochondrial-enriched pellets were solubilized in a Solubilization Buffer (50 mM NaCl, 20 mM Bis-tris, 2 mM aminocaproic acid, 1 mM EDTA, 10% (v/v) glycerol, pH 7.0) supplemented with 1% digitonin and protease inhibitors cocktail. The solution was incubated on ice for 15 min and was centrifugated at 18,000 × *g* for 30 min at 4 °C. The supernatants were transferred to the new tubes and protein concentrations were measured using the Bradford method. One-tenth of 10×BN-PAGE loading dye (100 mM Bis-Tris, pH 7.0, 500 mM aminocaproic acid and 5% Coomassie Blue G-250) was added for loading onto the gel.

For BN-PAGE, continuous 6%–15% gels were made using a gradient mixer. The gels were made with acrylamide/bis-acrylamide solutions in 3×BN gel buffer (1.5 M aminocaproic acid, 150 mM Bis-Tris, pH 7.0). The separating gel was composed of 6% acrylamide/bis-acrylamide alone or 15% acrylamide/bis-acrylamide with 20% (w/v) glycerol. The stacking gel was composed of 4% acrylamide/bis-acrylamide and layered onto the separating gel. Electrophoresis was performed using Blue cathode buffer (50 mM tricine, 15 mM Bis-Tris, pH 7.0, 0.02% (w/v) Coomassie blue G-250) and anode buffer (50 mM Bis-Tris, pH 7.0). When the dye reaches 2/3 of the gel length, the Blue cathode buffer was replaced with Cathode buffer (50 mM tricine, 15 mM Bis-Tris, pH 7.0). Following electrophoresis, proteins were transferred to a PVDF membrane that was soaked in 8% acetic acid for 20 min, rinsed with water and air-dried. The membrane was washed with methanol until most of the residual Coomassie Blue G-250 was removed, before proceeding to standard immunoblotting with specific antibodies against complex I (NDUFB8, NDUFV2, NDUFS1, NDUFV2, NDUFA9, NDUFB6, NDUFC2, NDUFA13), CIII (UQCRFS1 or UQCRQ), CIV (COX4). The immunoblot bands with high molecular mass were used to reflect the CI-, CIII-, or CIV-containing supercomplexes. For 2D BN/SDS–PAGE analysis, BN-PAGE lane per sample was excised from the gel and incubated with a Denaturing Solution (1x SDS loading buffer) on an orbital shaker for 30 min at RT. Then, the lanes were inserted into 12 or 15% SDS–PAGE in a 90 orientation and separated following electrophoresis.

#### Complex I in-gel activity

Complex I activity was assessed by incubating the native gels in the assay solution containing NADH (0.1 mg/ml), nitrotetrazolium blue chloride (NBT, 2.5 mg/ml), and 5 mM tris-HCl (pH 7.4), denoted by the appearance of dark purple colour. The intensities of reduced NBT were normalized to TOM20 levels of the same samples, detected by SDS–PAGE followed by immunoblotting. Gels were imaged using Tanon 1600 (Tanon).

#### Transmission electron microscopy

Cells were fixed in 0.1 M sodium cacodylate buffer (pH 7.2) containing 2% paraformaldehyde and 3% glutaraldehyde for 2–3 h at RT and overnight at 4 °C, post-fixed with 1% osmium tetroxide and 1.5% potassium ferrocyanide (pH 7.4) for 1 h at RT, then block stained in 0.25% aqueous uranyl acetate, processed in a standard manner and embedded in EMbed 812 (Electron Microscopy Sciences). Ultrathin sections (60 nm) were cut, mounted on copper grids and stained with uranyl acetate and lead citrate. Stained grids were viewed with a Tecnai T12 (FEI) transmission electron microscope operating at 120 kV. Images were captured with a Veleta (Olympus Soft Imaging System) digital camera and analyzed with ImageJ software.

#### Proteomic analysis of BN-PAGE fractions

For proteomic analysis of BN-PAGE fractions, digitonin solubilized mitochondria proteins were subjected to BN-PAGE analysis. Three supercomplex bands per example, determined in Fig. 1A,B, were excised and cut into 1-mm^3^ pieces followed by in-gel digestion using LysC and trypsin enzymes. After digestion, the peptides were desalted and analyzed using an Orbitrap Exploris 480 mass spectrometer (Thermo). Raw data were analyzed with MaxQuant. The human database was from UniProt (Proteome ID: UP000005640). Protein intensities based on label-free quantification (LFQ) were normalized to the maximum abundance across all samples analyzed. Thirteen bands per example, determined in Fig. 2B, were excised and cut into 1-mm^3^ pieces followed by in-gel digestion using LysC and trypsin enzymes. After digestion, the peptides were desalted and analyzed using a timsTOF Pro2 mass spectrometer (Bruker). Raw data were analyzed with MaxQuant. The human database was from UniProt (Proteome ID: UP000005640). Protein intensities based on label-free quantification (LFQ) were normalized to the maximum abundance across all samples analyzed. Heatmaps of proteomic analysis of BN-PAGE fractions consisting of proteins grouped according to the migration profiles in BN-PAGE electrophoresis were generated by representing the normalized abundance in each gel slice by a three-color gradient (blue/white/orange) (Cabrera-Orefice et al, 2022; Guerrero-Castillo et al, 2017). Microsoft Excel was used for visualization and analysis.

#### Mass-spectrometry analysis of mitochondrial proteome

Mitochondria-enriched fractions isolated from interphase (G1/S) and mitotic HeLa cells, determined in Fig. 1D,E, were digested with trypsin and analyzed by LC-MS/MS on timsTOF Pro mass spectrometry. The DIA data were processed using DIA-NN search engine (v.1.8). Tandem mass spectra were searched against Homo_sapiens_9606_SP_20231220.fasta (20429 entries) concatenated with reverse decoy database. Trypsin/P was specified as a cleavage enzyme allowing up to 1 missing cleavage. FDR was adjusted to <1%. LC-MS/MS analysis was performed twice with consistent results. All raw data are deposited in iProx and representative spectra from one run are shown. For the experiment, identified proteins were selected as mitochondrial if they were found in MitoCarta 3.0 (Rath et al, 2021).

#### Quantification and statistical analysis

Statistical analyses were performed using GraphPad Prism v8.0.2 software (La Jolla, CA, USA). Differences between two groups were analyzed by two-tailed Student’s *t* test. Differences between three or more groups were analyzed by one-way ANOVA. Comparisons between more than two variables were analyzed by two-way ANOVA. For each experiment, at least three biological replicates were analyzed. The data were presented as mean ± SD. Statistical significance was defined by *P < 0.05, **P < 0.01, ***P < 0.001, ****P < 0.0001 and (ns) *P* > 0.05 for not significant.

## Supplementary information

Peer Review File

Source data Fig. 1

Source data Fig. 2

Source data Fig. 3

Source data Fig. 4

Source data Fig. 5

Source data Fig. 6

Expanded View Figures

## Data Availability

The original mass spectrometry data and the database search results produced in this study are available via the ProteomeXchange consortium (through the iProX partner repository) under the following accessions and URLs: PXD078881 (Fig. 1B, https://www.iprox.cn/page/project.html?id=IPX0017441000), PXD078879 (Fig. 1E, https://www.iprox.cn/page/project.html?id=IPX0017437000), PXD079047 (Fig. 2B, https://www.iprox.cn/page/project.html?id=IPX0017444000). The source data of this paper are collected in the following database record: biostudies:S-SCDT-10_1038-S44319-026-00937-8.
