# Supplementary material for: Mitotic assembly of respiratory supercomplexes ensures accurate cell division and fuels tumor growth
Source: EMBO Rep. 2026 Sep 23;27(20):6061–90. doi: 10.1038/s44319-026-00937-8 (PMC13694716; doi:10.1038/s44319-026-00937-8)
Supplement: Supplementary file 1 [file 44319_2026_937_MOESM1_ESM.pdf]

EMBO reports

Mitotic Assembly of Respiratory Supercomplexes Ensures Accurate Cell Division and Fuels Tumor Growth

Ting Wang, Aoxing Cheng, Lingyu Kong, Weiyi You, Jihua Liu, Xing Liu, Xuebiao Yao, Yi Yang, Yuzheng Zhao, Weiwei Yang, Kaiguang Zhang, Zhenye Yang, and Jing Guo

Corresponding author(s): Jing Guo (jguo2013@ustc.edu.cn) , Zhenye Yang (zhenye@ustc.edu.cn), Weiwei Yang (wyang@sibcb.ac.cn), Kaiguang Zhang (Zhangkaiguang@ustc.edu.cn)

Review Timeline:

Submission Date:	17th Jan 26
Editorial Decision:	3rd Mar 26
Revision Received:	5th Jun 26
Editorial Decision:	20th Aug 26
Revision Received:	26th Aug 26
Accepted:	7th Sep 26

Editor: Kurt Weir / Achim Breiling

Transaction Report:

(Note: With the exception of the correction of typographical or spelling errors that could be a source of ambiguity, letters and reports are not edited. Depending on transfer agreements, referee reports obtained elsewhere may or may not be included in this compilation. Referee reports are anonymous unless the Referee chooses to sign their reports.)

1st Editorial Decision **3rd Mar 2026**

Dear Dr. Guo

Thank you for the submission of your research manuscript to our journal. We have now received the full set of referee reports that is copied below.

As you will see, the referees acknowledge that the findings are interesting and that the conclusions are overall supported by the data presented but they also raise a number of concerns and have suggestions how to further strengthen the data, particularly the inclusion of additional control experiments to rule out alternative explanations and the revision of statistical analyses.

Given these constructive comments, we would like to invite you to revise your manuscript with the understanding that the referee concerns (as detailed above and in their reports) must be fully addressed and their suggestions taken on board. Please address all referee concerns in a complete point-by-point response. Acceptance of the manuscript will depend on a positive outcome of a second round of review. It is EMBO Reports policy to allow a single round of revision only and acceptance or rejection of the manuscript will therefore depend on the completeness of your responses included in the next, final version of the manuscript.

We realize that it is difficult to revise to a specific deadline. In the interest of protecting the conceptual advance provided by the work, we recommend a revision within 3 months (June 3rd). Please discuss the revision progress ahead of this time with the editor if you require more time to complete the revisions.

I am also happy to discuss the revision further via e-mail or a video call, if you wish.

You can either publish the study as a short report or as a full article. For short reports, the revised manuscript should not exceed 27,000 characters (including spaces but excluding materials & methods and references) and 5 main plus 5 expanded view figures. The results and discussion sections must further be combined, which will help to shorten the manuscript text by eliminating some redundancy that is inevitable when discussing the same experiments twice. For a normal article there are no length limitations, but it should have more than 5 main figures and the results and discussion sections must be separate. In both cases, the entire materials and methods must be included in the main manuscript file.

================
IMPORTANT NOTE:

We perform an initial quality control of all revised manuscripts before re-review. Your manuscript will FAIL this control and the handling will be delayed IN CASE the following APPLIES:

1) A data availability section providing access to data deposited in public databases is missing. If you have not deposited any data, please add a sentence to the data availability section that explains that.

2) Your manuscript contains statistics and error bars based on n=2. Please use scatter blots in these cases. No statistics should be calculated if n=2.

When submitting your revised manuscript, please carefully review the instructions that follow below. Failure to include requested items will delay the evaluation of your revision.

================

When submitting your revised manuscript, we will require:

1) a .docx formatted version of the manuscript text (including legends for main figures, EV figures and tables). Please make sure that the changes are highlighted to be clearly visible.

2) individual production quality figure files as .eps, .tif, .jpg (one file per figure).
Please download our Figure Preparation Guidelines (figure preparation pdf) from our Author Guidelines pages https://www.embopress.org/page/journal/14693178/authorguide for more info on how to prepare your figures.

3) a .docx formatted letter INCLUDING the reviewers' reports and your detailed point-by-point responses to their comments. As part of the EMBO Press transparent editorial process, the point-by-point response is part of the Review Process File (RPF), which will be published alongside your paper.

4) a complete author checklist, which you can download from our author guidelines (<https://www.embopress.org/page/journal/14693178/authorguide>). Please insert information in the checklist that is also

reflected in the manuscript. The completed author checklist will also be part of the RPF.

5) Please note that all corresponding authors are required to supply an ORCID ID for their name upon submission of a revised manuscript (<https://orcid.org/>). Please find instructions on how to link your ORCID ID to your account in our manuscript tracking system in our Author guidelines (<https://www.embopress.org/page/journal/14693178/authorguide#authorshipguidelines>)

6) We replaced Supplementary Information with Expanded View (EV) Figures and Tables that are collapsible/expandable online. A maximum of 5 EV Figures can be typeset. EV Figures should be cited as 'Figure EV1, Figure EV2" etc... in the text and their respective legends should be included in the main text after the legends of regular figures.

- For the figures that you do NOT wish to display as Expanded View figures, they should be bundled together with their legends in a single PDF file called *Appendix*, which should start with a short Table of Content. Appendix figures should be referred to in the main text as: "Appendix Figure S1, Appendix Figure S2" etc. See detailed instructions regarding expanded view here: <https://www.embopress.org/page/journal/14693178/authorguide#expandedview>

- Additional Tables/Datasets should be labeled and referred to as Table EV1, Dataset EV1, etc. Legends have to be provided in a separate tab in case of .xls files. Alternatively, the legend can be supplied as a separate text file (README) and zipped together with the Table/Dataset file.

7) Before submitting your revision, primary datasets (and computer code, where appropriate) produced in this study need to be deposited in an appropriate public database (see < https://www.embopress.org/page/journal/14693178/authorguide#dataavailability>).

Please remember to provide a reviewer password if the datasets are not yet public.

The accession numbers and database should be listed in a formal "Data Availability " section (placed after Materials & Method) that follows the model below (see also < https://www.embopress.org/page/journal/14693178/authorguide#dataavailability>). Please note that the Data Availability Section is restricted to new primary data that are part of this study.

Data availability

The datasets (and computer code) produced in this study are available in the following databases:

- RNA-Seq data: Gene Expression Omnibus GSE46843 (https://www.ncbi.nlm.nih.gov/geo/query/acc.cgi?acc=GSE46843)
- [data type]: [name of the resource] [accession number/identifier/doi] ([URL or identifiers.org/DATABASE:ACCESSION])

*** Note - All links should resolve to a page where the data can be accessed. ***

8) At EMBO Press we ask authors to provide source data for the main manuscript figures. You will receive a separate email with instructions for providing source data with your revised manuscript, including how to upload and organize the files.

Additional information on source data and instruction on how to label the files are available <https://www.embopress.org/page/journal/14693178/authorguide#sourcedata>

9) The journal requires a statement specifying whether or not authors have competing interests (defined as all potential or actual interests that could be perceived to influence the presentation or interpretation of an article). In case of competing interests, this must be specified in your disclosure statement. Further information: https://www.embopress.org/competing-interests

10) Figure legends and data quantification:
The following points must be specified in each figure legend:

- the name of the statistical test used to generate error bars and P values,
- the EXACT p-values,
- the number (n) of independent experiments (please specify technical or biological replicates) underlying each data point,
- the nature of the bars and error bars (s.d., s.e.m.)

- If the data are obtained from n {less than or equal to} 5, show the individual data points in addition to the SD or SEM.
- If the data are obtained from n {less than or equal to} 2, use scatter blots showing the individual data points.

Discussion of statistical methodology can be reported in the materials and methods section, but figure legends should contain a basic description of n, P and the test applied.

See also the guidelines for figure legend preparation:

https://www.embopress.org/page/journal/14693178/authorguide#figureformat

- Please also include scale bars in all microscopy images and define their size in the legend, not the image.

11) Our journal encourages inclusion of *data citations in the reference list* to directly cite datasets that were re-used and obtained from public databases. Data citations in the article text are distinct from normal bibliographical citations and should directly link to the database records from which the data can be accessed. In the main text, data citations are formatted as follows: "Data ref: Smith et al, 2001" or "Data ref: NCBI Sequence Read Archive PRJNA342805, 2017". In the Reference list, data citations must be labeled with "[DATASET]". A data reference must provide the database name, accession number/identifiers and a resolvable link to the landing page from which the data can be accessed at the end of the reference. Further instructions are available at <https://www.embopress.org/page/journal/14693178/authorguide#referencesformat>.

12) All Materials and Methods need to be described in the main text using our 'Structured Methods' format. According to this format, the Methods section includes a Reagents and Tools Table (listing key reagents, experimental models, software and relevant equipment and including their sources and relevant identifiers) followed by a Methods and Protocols section describing the methods, ideally using a step-by-step protocol format. The aim is to facilitate adoption of the methodologies across labs. Please download and fill our Reagents and Tools Table template (.docx), which you can find in our author guidelines: https://www.embopress.org/page/journal/14693178/authorguide#structuredmethods.
When submitting your revised manuscript, please do not include the Reagents and Tools Table in the Methods section of the manuscript but upload it as a separate file choosing the file type "Reagent Table".
An example of a Method paper with Structured Methods can be found here:
https://www.embopress.org/doi/10.15252/msb.20178071.

13) As part of the EMBO publication's Transparent Editorial Process, EMBO Reports publishes online a Review Process File to accompany accepted manuscripts. This File will be published in conjunction with your paper and will include the referee reports, your point-by-point response and all pertinent correspondence relating to the manuscript.

You are able to opt out of this by letting the editorial office know (emboreports@embo.org). If you do opt out, the Review Process File link will point to the following statement: "No Review Process File is available with this article, as the authors have chosen not to make the review process public in this case."

We would also welcome the submission of cover suggestions, or motifs to be used by our Graphics Illustrator in designing a cover.

I look forward to seeing a revised form of your manuscript when it is ready and please let me know if you have questions or comments regarding the revision.

Yours sincerely,

Kurt Weir
Editor
EMBO Reports

Referee #1:

This is a conceptually strong and experimentally ambitious manuscript that identifies a CDK1-dependent translational upregulation of NDUFA3, which promotes respirasome assembly during mitosis. The authors convincingly show that this mechanism is critical to maintain mitotic redox homeostasis, preserve cristae structure, and ensure faithful chromosome segregation. The integration of complexome profiling, BN-PAGE biochemistry, proteomics, live-cell imaging, and in vivo tumor models is impressive and provides a compelling multi-angle dataset. The manuscript is already strong in its current form. The following suggestions could further increase clarity, mechanistic depth, and overall impact.

1. The manuscript provides strong evidence that NDUFA3 levels increase during mitosis and that its depletion leads to impaired respirasome formation. In Fig. 2, NDUFA3 knockdown appears to reduce several CI subunits broadly (e.g., NDUFB6, NDUFB8, NDUFV2). While this does not contradict the authors' model, it suggests a more global destabilisation of CI, not exclusively a PP-a-restricted effect. BN-PAGE is helpful qualitatively, but a more detailed characterisation of assembly intermediates (e.g., using complexome profiling or quantitative MS of mitochondrial fractions) would strengthen the interpretation that NDUFA3's primary role is in early PP-a integration rather than general membrane-arm stability. This is not required for publication, but it would clarify the mechanistic degree of the observed CI defects.

2. The manuscript provides solid evidence that CDK1 promotes ribosome loading onto NDUFA3 mRNA. The discussion nicely outlines possible models, like phosphorylation of translation initiation factors, ORF/UTR structural features, mTOR-independent translational regulation, etc. Since the authors already discuss this model space well, the study would benefit from a targeted experimental test. For example, assessing the phosphorylation state of known CDK1-regulated translational factors (such as eIF4B, eIF4E, or RPL12) under the conditions used in Fig. 3 would help anchor the proposed mechanism more concretely. Even a negative result would be informative and would help narrow down the possible regulatory pathways. Again, this is an enhancement, not a requirement.

3. The use of SCAF1 knockdown as an orthogonal strategy to disrupt respirasomes is appreciated, and the authors correctly interpret that a similar phenotype supports a broader supercomplex-assembly dependency during mitosis. However, SCAF1's role in $CIII_2$-CIV supercomplex stabilisation varies substantially in human cells, and its knockdown cannot always be interpreted as cleanly "respirasome-specific." To more clearly separate respirasome disassembly from general CI inhibition, the authors could consider treating mitotic cells with low-dose rotenone, which inhibits CI activity without substantially interfering with CI assembly. Further, using Complex III inhibitors (e.g., antimycin A), which generate high ROS specifically through the Q-cycle could be applied. If these inhibitors fail to phenocopy NDUFA3 knockdown, it would strengthen the conclusion that respirasome integrity-not simply CI or CIII activity-is uniquely required for mitotic redox homeostasis. This would reinforce the specificity of the proposed mechanism.

This study provides an important mechanistic link between mitotic bioenergetics, respirasome regulation, and chromosome segregation fidelity. It identifies a CDK1-dependent translational regulatory axis that is both novel and biologically meaningful. The breadth of methods, clarity of the main conclusions, and the thorough discussion make this manuscript highly suitable for publication. The suggestions above are intended to further strengthen an already compelling and well-executed study.

Referee #2:

In this report, Ting Wang et al determine how mitochondrial respiratory chain enzymes change during the cell cycle in synchronized HeLa cells. They find that particularly complex I subunits are induced during mitosis, leading to the formation of respirasomes that are almost absent during interphase. They then knockdown a complex I subunit and find that respiration and connected redox control is important for the faithful segregation of chromosomes, which in turn affects tumor growth. Overall, this is an interesting study that very well describes these changes, using sets of high-quality experiments. The figures are clear and the text well written. The link between respiration, redox control and fidelity of chromosome segregation is interesting but not conceptually novel. The authors attempt to introduce a new twitch to this concept by suggesting that it is the assembly of respiratory chain enzymes into respirasomes that is important, but the causality implied by this claim is not sufficiently elaborated.

Major point:
The authors observe using MS analyses of high molecular weight slices of a BN-PAGE that several complex I subunits are lowly expressed in interphase and induced during mitosis, leading to an almost absence of respirasomes containing Complex I, III and IV during interphase. The main argument of this work is that it is the assembly of respiratory chain complexes into respirasomes that is responsible for all the other effects reported in this story. In addition to presenting the MS work on fractions from the BN-PAGE, the authors should also present whole cell proteomics focusing on mitochondrial proteins as they are curated in mitocarta. Given the strong changes in certain Complex I subunits, it is more likely that the changes in respirasome assembly stem from a general up-regulation of respiratory capacity, ie inducing a change in cellular metabolism, which would then induce all the other effects. To investigate this, the authors should also examine respiratory capacity in interphase and mitotic cells, respectively, using the same experimental setup as in Fig 4.

Minor point:
The authors show series of data in diagrams without providing the basis for their statistics. n-numbers should be provided for all experiments where necessary, eg Figures 5A, 5B, 5C, S5B, S5G, 6B, 6D, S6E. Furthermore, why are the N identical for S4C and S4D?

Referee #3:

The study addresses a timely and interesting question of how mitochondrial respiratory chain organization adapts during mitosis and proposes that CDK1-driven NDUFA3 upregulation promotes respirasome assembly, thereby maintaining redox homeostasis and faithful chromosome segregation. The work is ambitious and spans proteomics, BN-PAGE, live-cell imaging, organoids and in vivo models. However, for me, several key claims are not sufficiently supported by the data as currently presented, particularly the interpretation of the "complexome" experiments, the BN-PAGE analysis, and the way statistics and controls are handled

across figures.
Major points:

1. The central "complexome profiling" experiment is not, in my view, a true complexome analysis but rather a targeted proteomic characterization of a few high-molecular-weight BN-PAGE bands that are assumed to correspond to respirasomes. The authors excise only three very large bands, rather than fractionating the entire lane into many slices (usually 50-60) and reconstructing continuous migration profiles for individual subunits, which is a key strength of genuine complexome profiling. As a consequence, they cannot rigorously distinguish mature respirasomes from other I/III/IV-containing assemblies, partially assembled intermediates, or heterogeneous aggregates. The assignment of these bands to a specific supercomplex species therefore remains largely qualitative. This is problematic because the central claim of "enhanced respirasome assembly in mitosis" relies on these bands being correctly identified and quantified as respirasomes. I would expect orthogonal validation (e.g. 2D BN/SDS, in-gel activity aligned with the proteomics, systematic assessment of stoichiometry and migration of CI, CIII and CIV across the entire gel) to underpin such a strong conclusion, and at present this level of rigor is missing.

2.Related to the above, the BN-PAGE data throughout the manuscript are mostly qualitative and even then not of highest quality, which substantially weakens the conclusions about altered supercomplex architecture. The key BN-PAGE figures are shown as "representative" blots, with very limited or no quantification of band intensities for CI, CIII, CIV, and supercomplexes across independent biological replicates, and there is heavy reliance on TOM20 alone as a loading control. This does not convincingly exclude differences in mitochondrial mass or extraction efficiency between interphase and mitotic samples. Detergent and solubilization conditions, which are known to strongly influence supercomplex stability, are only briefly described and are not systematically validated to demonstrate that the observed patterns are not artefacts of sample preparation. In addition, the assignment of individual BN bands to specific CI assembly intermediates or modules (PP-a, PP-b, PD-a, etc.) is based on single antibodies and schematic models; there is no rigorous mapping using multiple subunits per module, 2D BN/SDS or known assembly factor mutants. As a result, the narrative of a "hierarchical, modular assembly" in mitosis feels considerably more precise than what the band resolution and validation actually allow.
3.The mechanistic attribution of the observed phenotypes specifically to "respirasome disassembly" is not entirely convincing and, in my opinion, underestimates the contribution of more general CI dysfunction and mitochondrial stress. NDUFA3 is an accessory CI subunit required for early CI assembly; its depletion predictably disrupts CI integrity, reduces CI activity and lowers the abundance of multiple CI components. Many of the downstream readouts, like ROS accumulation, mitochondrial depolarization, cristae changes, are exactly what one would expect from a generic CI deficiency and impaired electron flow, regardless of the precise supercomplex architecture. The attempt to distinguish "respirasome-specific" effects via SCAF1 knockdown is helpful but not fully conclusive, as SCAF1 manipulations also have broader effects on CIII/CIV organization and overall respiration, and the data provided do not exhaustively document total CI and other complexes in this setting. Furthermore, the rescue strategy rests almost entirely on NAC, a very non-specific antioxidant and thiol donor; without additional genetic or pharmacological approaches (e.g. mitochondria-targeted ROS scavengers, partial CI inhibition in the absence of supercomplex disruption...), the causal chain from respirasome organization to ROS, Aurora inhibition and chromosome mis-segregation remains more correlative than the text suggests.

4.I am concerned about the way statistics and controls are handled across multiple figures, which affects my confidence in several of the quantitative claims. Many panels summarize complex biological phenomena (e.g. mitotic phenotypes, ROS, ΔΨm, NADPH, cristae morphology, organoid size, tumor growth) using simple two-group comparisons, often with very small numbers of biological replicates and without clear information on how sample size was determined, whether data distribution and variance assumptions were checked, or whether any correction for multiple testing was applied despite the large number of endpoints. This is particularly important for the in vivo tumor growth curves, where repeated measurements over time appear to be treated essentially as independent time points instead of being analyzed with an appropriate longitudinal approach or a global test. In addition, in several panels, controls are arbitrarily set to 1.0 and all experimental conditions are plotted relative to this baseline, effectively removing the variation of the control group from the statistical assessment and making it difficult to evaluate the true effect size and variability. This practice can artificially inflate apparent significance and obscures how noisy the control measurements actually are. I would strongly recommend re-plotting and re-analyzing the key datasets with absolute values, explicit reporting of n (biological vs technical), and more appropriate statistics given the number of comparisons and the repeated-measures nature of some experiments.

5. Finally, I feel the conceptual narrative tends to overreach what the current dataset can firmly support. Most of the in-depth mechanistic work is carried out in two cancer cell lines, with additional models used only for selected readouts, yet the discussion generalizes to "cancer cells" and "tumor progression" in a broad sense. In highly glycolytic cells, total ATP is maintained through glycolytic compensation, and the authors do not test conditions in which mitochondrial ATP becomes limiting (e.g. low glucose, galactose) or in which cells are truly OXPHOS-dependent, which would be important to underpin statements about respirasome assembly being "essential" for mitotic progression. The organoid and in vivo data, although interesting, are based on relatively small cohorts and show partial effects that can have multiple explanations in complex tumor ecosystems; the partial rescue by NAC in vivo is suggestive but not definitive. Taken together, I would encourage the authors to either temper their claims, framing this as an important example of mitotic mitochondrial remodeling in specific cancer contexts, or to provide additional data that more rigorously demonstrate that mitotic respirasome assembly is a central, targetable driver of chromosome stability and tumor growth across models.

Point-by-point responses

We sincerely thank the editor and reviewers for their insightful and constructive critiques. These comments prompted us to perform additional experiments and data re-analyses, which have strengthened our findings, further substantiated the central conclusions, and improved the overall rigor and impact of the manuscript. In the following pages, we provide a point-by-point response (**in blue text**) to each comment, along with the corresponding revisions implemented in the main text and supplementary materials.

Point-by-point response to referee's comments

Referee # 1 (Remarks to the Author):

This is a conceptually strong and experimentally ambitious manuscript that identifies a CDK1-dependent translational upregulation of NDUFA3, which promotes respirasome assembly during mitosis. The authors convincingly show that this mechanism is critical to maintain mitotic redox homeostasis, preserve cristae structure, and ensure faithful chromosome segregation. Th e integration of complexome profiling, BN-PAGE biochemistry, proteomics, live-cell imaging, and in vivo tumor models is impressive and provides a compelling multi-angle dataset. The manuscript is already strong in its current form. The following suggestions could further increase clarity, mechanistic depth, and overall impact.

***Response*:** We appreciate the reviewer's accurate summarization and positive feedback.

1. The manuscript provides strong evidence that NDUFA3 levels increase during mitosis and that its depletion leads to impaired respirasome formation. In Fig. 2, NDUFA3 knockdown appears to reduce several CI subunits broadly (e.g., NDUFB6, NDUFB8, NDUFV2). While this does not contradict the authors' model, it suggests a more global destabilisation of CI, not exclusively a PP-a-restricted effect. BN-PAGE is helpful qualitatively, but a more detailed characterisation of assembly intermediates (e.g., using complexome profiling or quantitative MS of mitochondrial fractions) would strengthen the interpretation that NDUFA3's primary role is in early PP-a integration rather than general membrane-arm stability. This is not required for publication, but it would clarify the mechanistic degree of the observed CI defects.

***Response*:** We thank the reviewer for this insightful suggestion. In response, we have performed more comprehensive complexome profiling (**Fig. 2B**) and 2D BN/SDS-PAGE (**Fig. 2C**) on mitochondrial proteins from G1/S and mitotic cells, with or without NDUFA3 knockdown. These orthogonal approaches map CI assembly intermediates and validate subunit stoichiometry. In addition to confirming that NDUFA3 knockdown reduces multiple CI subunit

levels in mitosis, both approaches consistently show that the primary defect is at the PP-a module (100-500 kDa), evidenced by accumulation of PP-b, PD-a, and Q intermediates and failed assembly of PD-b and N modules. Reduced CI/CIII/CIV supercomplexes with $CIII_2$/CIV accumulation were also observed (**Fig. 2B, C**). We propose that the broad CI subunit reduction is a consequence rather than the cause of impaired early assembly. Once assembly stalls at the PP-a module, unassembled subunits likely become destabilized and degraded. Increased CIII/CIV levels further suggest a compensatory response rather than general ETC collapse. Thus, NDUFA3 is specifically required for early PP-a integration during mitotic CI assembly. We appreciate the reviewer's constructive suggestion, which has helped refine our mechanistic interpretation.

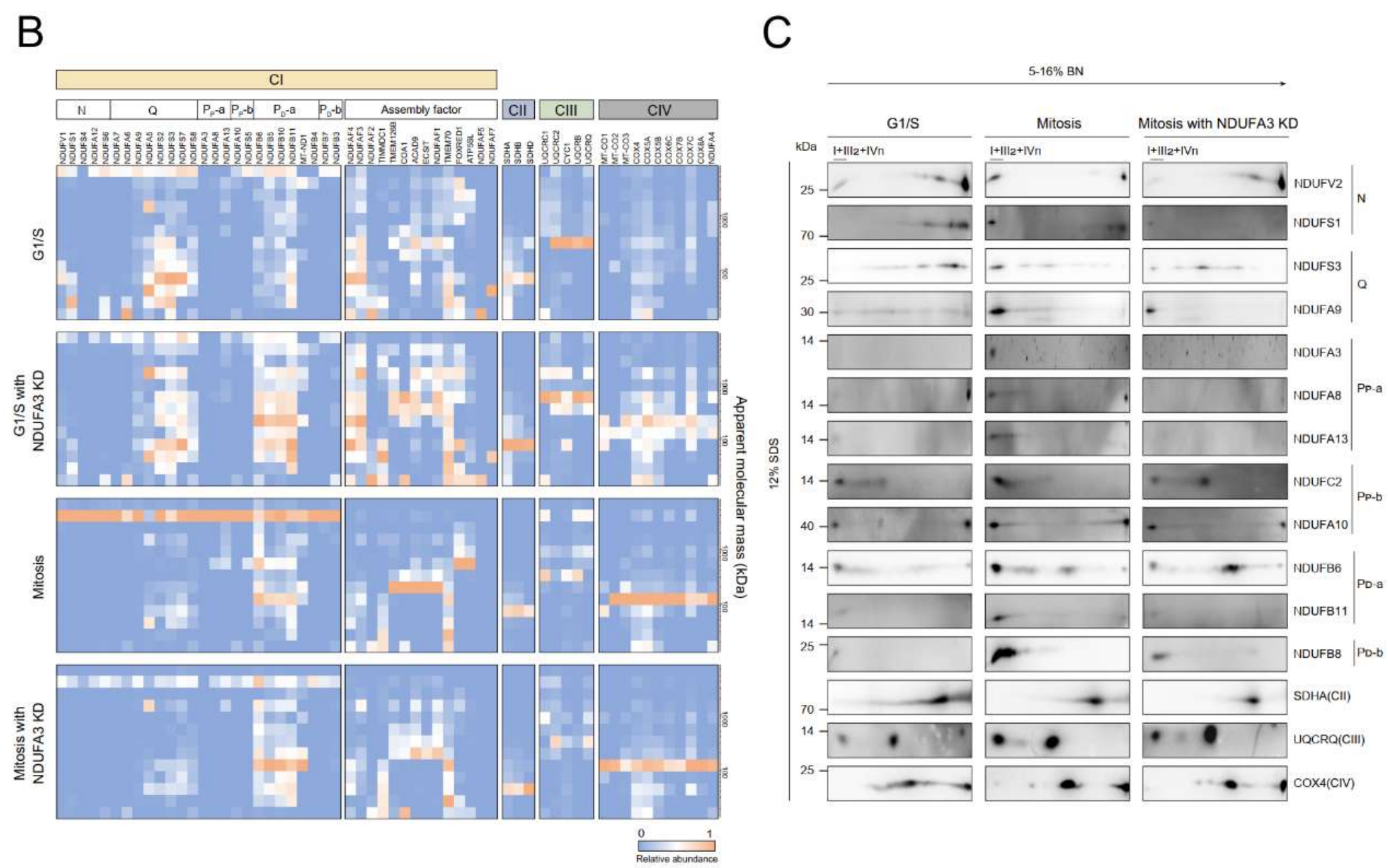

2. The manuscript provides solid evidence that CDK1 promotes ribosome loading onto NDUFA3 mRNA. The discussion nicely outlines possible models, like phosphorylation of translation initiation factors, ORF/UTR structural features, mTOR-independent translational regulation, etc. Since the authors already discuss this model space well, the study would benefit from a targeted experimental test. For example, assessing the phosphorylation state of known CDK1-regulated translational factors (such as eIF4B, eIF4E, or RPL12) under the conditions used in Fig. 3 would help anchor the proposed mechanism more concretely. Even a negative result would be informative and would help narrow down the possible regulatory pathways. Again, this is an enhancement, not a requirement.

Response: We thank the reviewer for this insightful suggestion. In response, we have performed western blotting using phospho-specific antibodies against known CDK1-regulated translation factors (eIF2α Ser51, 4EBP1 Thr37/46, S6 Ser235/236) in G1/S, mitotic, and mitotic cells

treated with the CDK1 inhibitor RO3306. For other factors such as RPL12, specific phospho-antibodies are currently unavailable, precluding their assessment. Our results show that CDK1 activity correlates with increased phosphorylation of 4EBP1 and S6 during mitosis (**Fig. EV3F**). In contrast, eIF2α did not exhibit CDK1-dependent phosphorylation changes. These findings provide positive evidence for CDK1-regulated translational factors while the negative result helps narrow down the possible regulatory pathways. Thus, CDK1 may promote ribosome loading onto NDUFA3 mRNA at least in part through phosphorylation of specific translation initiation factors.

F

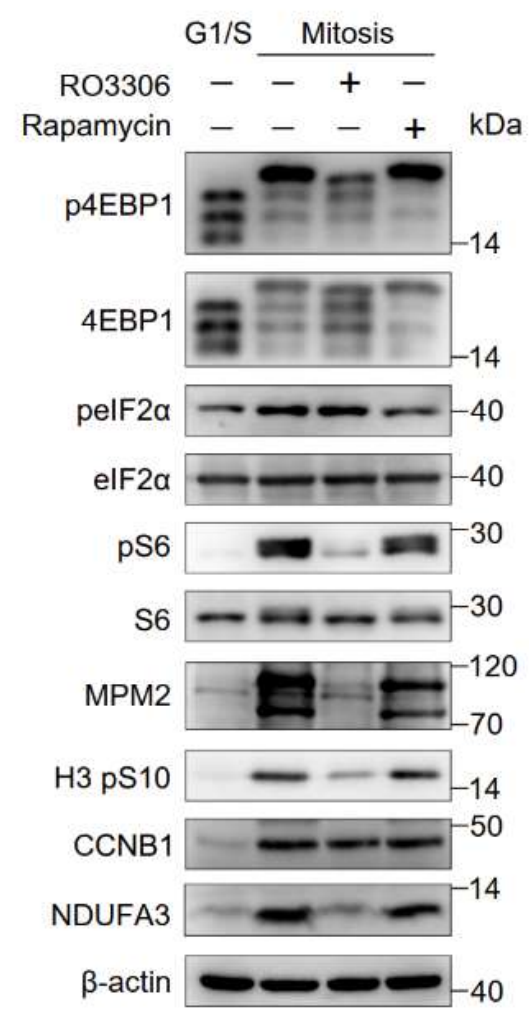

3. The use of SCAF1 knockdown as an orthogonal strategy to disrupt respirasomes is appreciated, and the authors correctly interpret that a similar phenotype supports a broader supercomplex-assembly dependency during mitosis. However, SCAF1's role in $CIII_2$-CIV supercomplex stabilisation varies substantially in human cells, and its knockdown cannot always be interpreted as cleanly "respirasome-specific." To more clearly separate respirasome disassembly from general CI inhibition, the authors could consider treating mitotic cells with low-dose rotenone, which inhibits CI activity without substantially interfering with CI assembly. Further, using Complex III inhibitors (e.g., antimycin A), which generate high ROS specifically through the Q-cycle could be applied. If these inhibitors fail to phenocopy NDUFA3 knockdown, it would strengthen the conclusion that respirasome integrity-not simply CI or CIII activity-is uniquely required for mitotic redox homeostasis. This would reinforce the specificity of the proposed mechanism.

***Response*:** We thank the reviewer for this important suggestion. In response, we have treated mitotic cells with low-dose rotenone or antimycin A. BN-PAGE confirmed that at the low concentrations used, respirasome integrity in mitotic cells remained unaffected (**Fig. EV5K**). Under these conditions, both inhibitors elevated ROS and reduced mitochondrial membrane

potential (**Fig. EV5L, M**). Neither inhibitor induced chromosome alignment or segregation defects in mitosis (**Fig. EV5L, M**). At higher concentrations, both inhibitors caused cell death prior to mitotic entry, precluding assessment of mitotic progression. Thus, pharmacological inhibition of CI or CIII activity, in the absence of respirasome disassembly, unable to phenocopy NDUFA3 knockdown. These results strengthen the conclusion that respirasome integrity—not merely CI or CIII activity—is uniquely required for mitotic redox homeostasis and chromosome segregation.

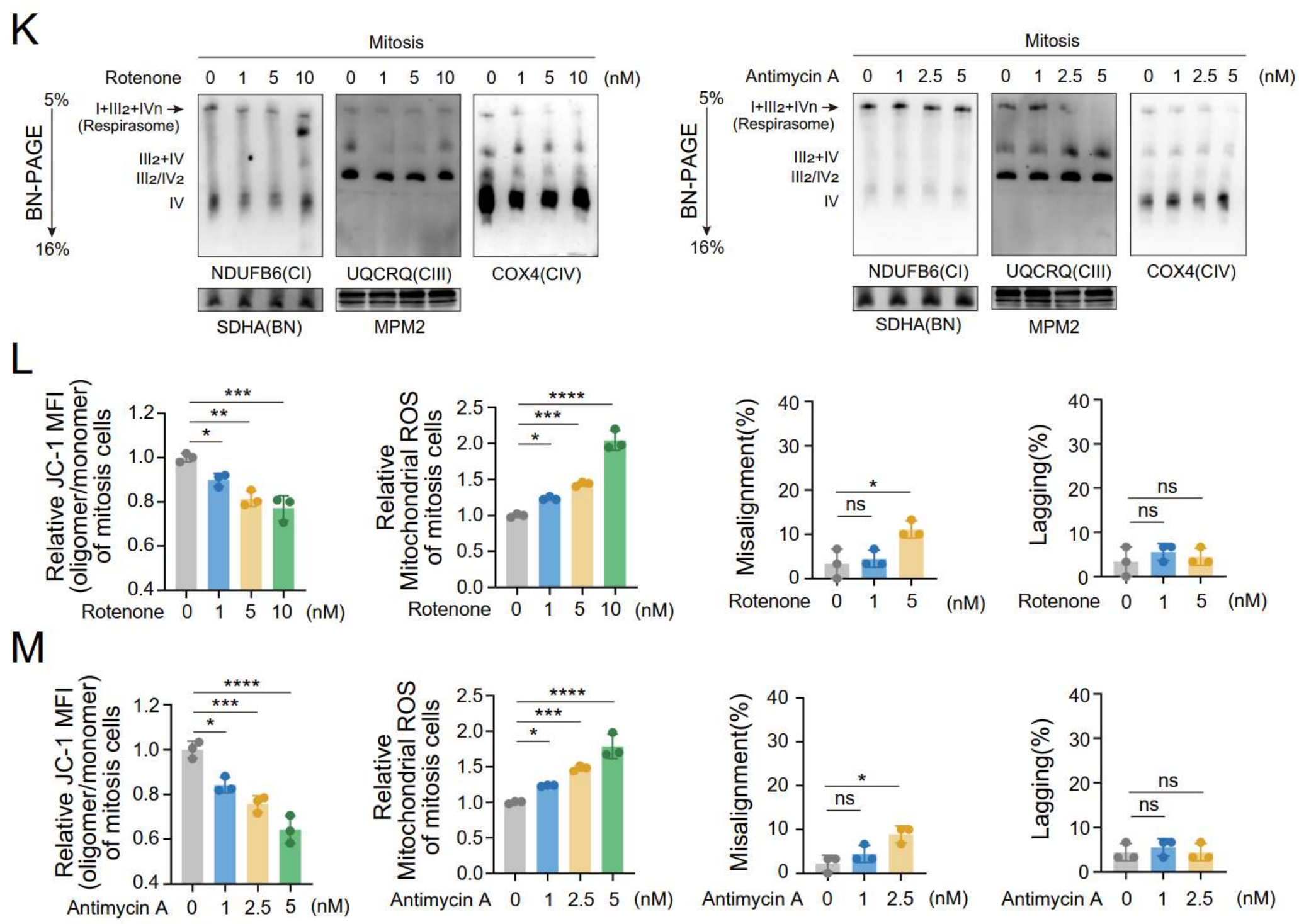

This study provides an important mechanistic link between mitotic bioenergetics, respirasome regulation, and chromosome segregation fidelity. It identifies a CDK1-dependent translational regulatory axis that is both novel and biologically meaningful. The breadth of methods, clarity of the main conclusions, and the thorough discussion make this manuscript highly suitable for publication. The suggestions above are intended to further strengthen an already compelling and well-executed study.

***Response*: We appreciate the reviewer's positive feedback and constructive suggestions.**

Referee 2

In this report, Ting Wang et al determine how mitochondrial respiratory chain enzymes change during the cell cycle in synchronized HeLa cells. They find that particularly complex I subunits are induced during mitosis, leading to the formation of respirasomes that are almost absent during interphase. They then knockdown a complex I subunit and find that respiration and connected redox control is important for the faithful segregation of chromosomes, which in turn affects tumor growth. Overall, this is an interesting study that very well describes these changes, using sets of high-quality experiments. The figures are clear and the text well written. The link between respiration, redox control and fidelity of chromosome segregation is interesting but not conceptually novel. The authors attempt to introduce a new twitch to this concept by suggesting that it is the assembly of respiratory chain enzymes into respirasomes that is important, but the causality implied by this claim is not sufficiently elaborated.

Major point:

1. The authors observe using MS analyses of high molecular weight slices of a BN-PAGE that several complex I subunits are lowly expressed in interphase and induced during mitosis, leading to an almost absence of respirasomes containing Complex I, III and IV during interphase. The main argument of this work is that it is the assembly of respiratory chain complexes into respirasomes that is responsible for all the other effects reported in this story. In addition to presenting the MS work on fractions from the BN-PAGE, the authors should also present whole cell proteomics focusing on mitochondrial proteins as they are curated in mitocarta.

Response**:** We thank the reviewer for the insightful comments and suggestions. In addition to the MS analysis of high molecular weight BN-PAGE fractions, we have also performed MS analysis on mitochondrial-enriched fractions isolated from interphase and mitotic cells (**Fig. 1D, E**). This analysis specifically captures mitochondrial proteins curated in the Mitocarta database (PMID: 33174596) and indeed reveals mitotic induction of specific Complex I subunits. Importantly, these findings are consistent with previously published datasets (PMID: 26305499, PMID: 29052541, PMID: 33627808), which collectively demonstrate that many mitochondrial proteins are globally upregulated during mitosis.

2. Given the strong changes in certain Complex I subunits, it is more likely that the changes in respirasome assembly stem from a general up-regulation of respiratory capacity, ie inducing a change in cellular metabolism, which would then induce all the other effects. To investigate this, the authors should also examine respiratory capacity in interphase and mitotic cells,

respectively, using the same experimental setup as in Fig 4.

***Response*:** We thank the reviewer for this important suggestion. In response, we measured oxygen consumption rate (OCR) and ATP levels in G1/S-arrested and mitotic cells under the same experimental conditions as in Fig. 4. Our results show that mitotic cells exhibit elevated OCR and ATP levels (**Fig. 4B, C**). We agree with the reviewer that these findings do not formally exclude the possibility that a general upregulation of respiratory capacity contributes to respirasome assembly. However, current literatures (PMID: 31023583, PMID: 27916530, PMID: 33027658, PMID: 27799543) indicate that reduced respiratory capacity, rather than increased capacity, can promote respirasome assembly under certain conditions. Given that the relationship between metabolic state and respirasome assembly is likely complex and context-dependent, we interpret our data as being consistent with the model that enhanced expression of Complex I subunits drives respirasome assembly during mitosis. Nevertheless, we acknowledge that the observed metabolic changes may also represent a concurrent phenomenon rather than a distinct primary cause. We have revised the text accordingly to present this interpretation more cautiously.

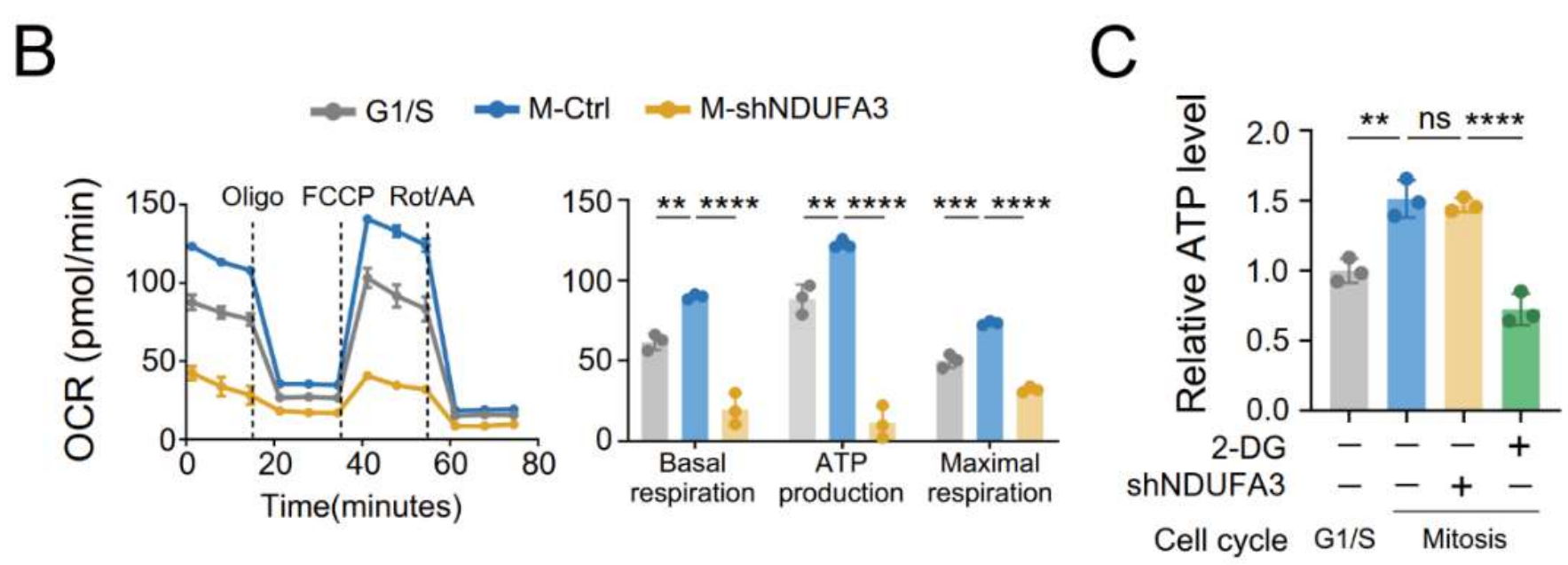

Minor point:

The authors show series of data in diagrams without providing the basis for their statistics. n-numbers should be provided for all experiments where necessary, eg Figures 5A, 5B, 5C, S5B, S5G, 6B, 6D, S6E. Furthermore, why are the N identical for S4C and S4D?

***Response*:** We thank the reviewer for the careful observation regarding statistical reporting. In response, we have revised all figure legends to explicitly indicate the number of independent biological replicates (n-numbers) for each experiment, including those requested for **Figures 5A–5C, S5B, S5G, 6B, 6D, and S6E**. Regarding Figures S4C and S4D, we verified the raw data and identified a duplication error in the n-number reporting. To resolve this, we have now merged these two panels into a single figure panel (**new Fig. 4E**) with a corrected and clearly stated n-number. All corresponding statistical analyses have been updated accordingly. We believe these revisions address the reviewer's concerns.

Referee 3

The study addresses a timely and interesting question of how mitochondrial respiratory chain organization adapts during mitosis and proposes that CDK1-driven NDUFA3 upregulation promotes respirasome assembly, thereby maintaining redox homeostasis and faithful chromosome segregation. The work is ambitious and spans proteomics, BN-PAGE, live-cell imaging, organoids and in vivo models. However, for me, several key claims are not sufficiently supported by the data as currently presented, particularly the interpretation of the "complexome" experiments, the BN-PAGE analysis, and the way statistics and controls are handled across figures.

***Response*:** We thank the very constructive criticisms and suggestions from the reviewer. We have performed more advanced experiments, strictly examined the statistics and control, and elaborated the interpretation in the revised manuscript.

Major points:

1. The central "complexome profiling" experiment is not, in my view, a true complexome analysis but rather a targeted proteomic characterization of a few high-molecular-weight BN-PAGE bands that are assumed to correspond to respirasomes. The authors excise only three very large bands, rather than fractionating the entire lane into many slices (usually 50-60) and reconstructing continuous migration profiles for individual subunits, which is a key strength of genuine complexome profiling. As a consequence, they cannot rigorously distinguish mature respirasomes from other I/III/IV-containing assemblies, partially assembled intermediates, or heterogeneous aggregates. The assignment of these bands to a specific supercomplex species therefore remains largely qualitative. This is problematic because the central claim of "enhanced respirasome assembly in mitosis" relies on these bands being correctly identified and quantified as respirasomes. I would expect orthogonal validation (e.g. 2D BN/SDS, in-gel activity aligned with the proteomics, systematic assessment of stoichiometry and migration of CI, CIII and CIV across the entire gel) to underpin such a strong conclusion, and at present this level of rigor is missing.

***Response*:** We thank the reviewer for this critical and constructive feedback.

First, to address the concern that our initial approach does not constitute true complexome profiling, we have now performed a more comprehensive analysis by slicing entire BN-PAGE lanes into 13 consecutive fractions, followed by LC-MS/MS analysis (PMID: 34038727). This allowed us to reconstruct continuous migration profiles for all OXPHOS subunits across the gel (new **Fig. 2B**), providing a more rigorous basis for identifying respiratory chain complexes and supercomplexes.

Second, to provide orthogonal validation of the assembly status and stoichiometry of

Complex I, III, and IV, we performed 2D BN/SDS-PAGE coupled with immunoblotting targeting multiple modules of Complex I (N, Q, PP-a, PP-b, PD-a, PD-b), as well as subunits of Complex III and Complex IV (**Fig. 2C**). This approach enables us to distinguish fully assembled respirasomes from partial assemblies or intermediates.

Our combined results show that interphase cells accumulate various assembly intermediates of Complex I, whereas mitotic cells exhibit reduced levels of these intermediates and a concomitant increase in mature respirasomes. These findings support the conclusion that mitotic progression actively promotes respirasome maturation, rather than merely increasing subunit expression.

We greatly appreciate the reviewer's rigorous assessment, which has substantially strengthened our study.

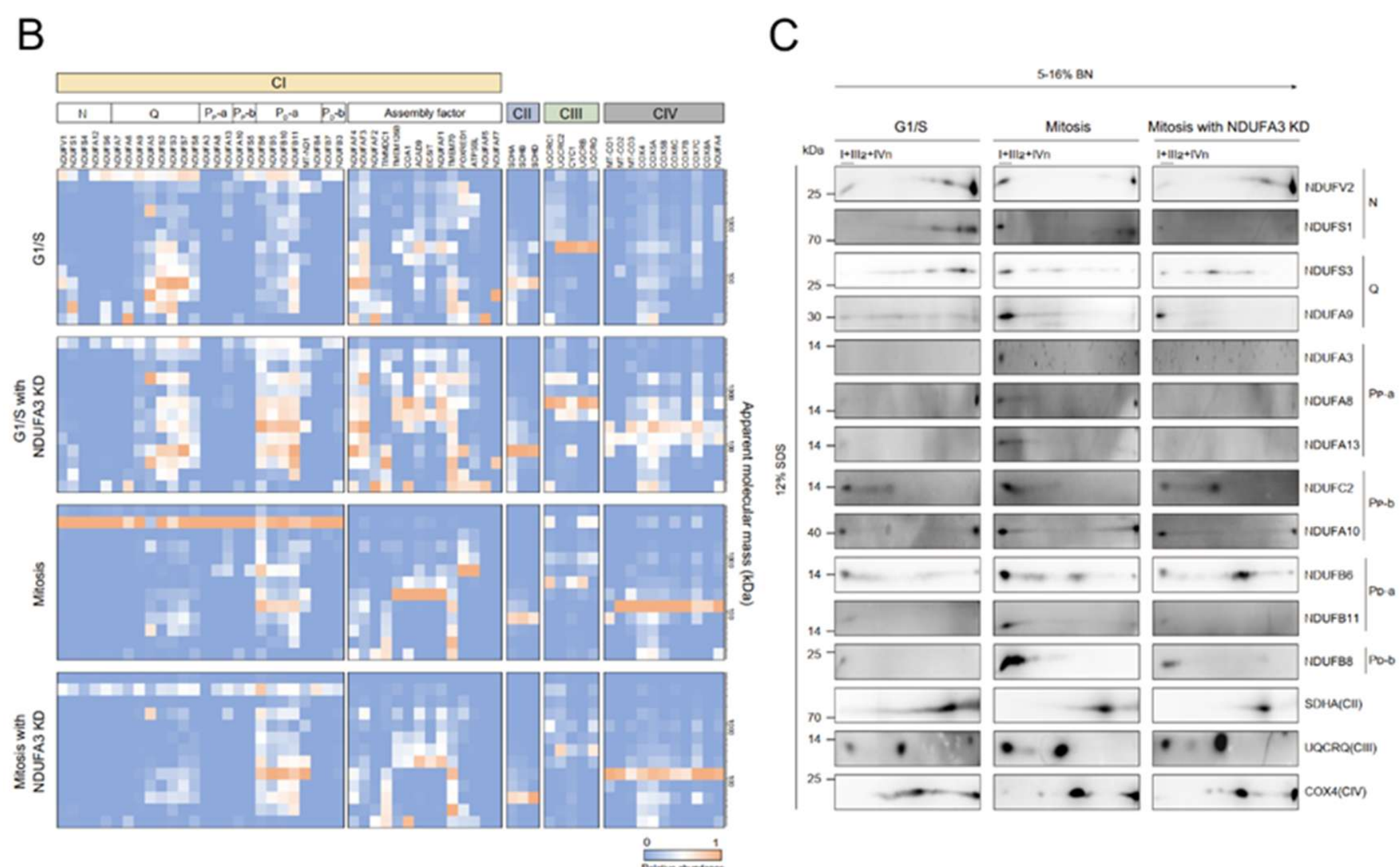

2. Related to the above, the BN-PAGE data throughout the manuscript are mostly qualitative and even then not of highest quality, which substantially weakens the conclusions about altered supercomplex architecture. The key BN-PAGE figures are shown as "representative" blots, with very limited or no quantification of band intensities for CI, CIII, CIV, and supercomplexes across independent biological replicates, and there is heavy reliance on TOM20 alone as a loading control. This does not convincingly exclude differences in mitochondrial mass or extraction efficiency between interphase and mitotic samples.

***Response*:** We thank the reviewer for this critical feedback. To address the concerns regarding the qualitative nature and limited quantification of our BN-PAGE data, we have undertaken the following revisions:

1. Quantification across biological replicates: All key BN-PAGE experiments have now

been performed with at least three independent biological replicates. Band intensities corresponding to Complex I (CI), Complex III (CIII), Complex IV (CIV), and supercomplexes have been quantified and subjected to statistical analysis. These quantified data are presented in revised figures (**Fig. 1C; Fig. EV2A–C; Fig. 3F**).

2. Improved loading control: In addition to TOM20, we have now included SDHA as a second loading control for BN-PAGE to more reliably normalize for mitochondrial mass and extraction efficiency between G1/S and mitotic samples. This dual-control approach is applied across the revised figures, including **Fig. 1C; Fig. EV1A–F; Fig. 2B–C; Fig. EV2A–C; Fig. 3F; and Fig. EV5F, I, K**.

All representative blots are now accompanied by corresponding quantification data from multiple replicates in the revised manuscript. We believe these enhancements substantially improve the rigor and interpretability of our BN-PAGE analyses.

Detergent and solubilization conditions, which are known to strongly influence supercomplex stability, are only briefly described and are not systematically validated to demonstrate that the observed patterns are not artefacts of sample preparation.

***Response*:** We thank the reviewer for raising this important methodological concern. To address whether the observed supercomplex patterns are influenced by detergent and solubilization conditions, we systematically varied the detergent-to-protein ratios for both G1/S and mitotic mitochondrial samples. Specifically, we tested digitonin concentrations ranging from 4–20 g/g protein and DDM concentrations ranging from 1–16 g/g protein, followed by BN-PAGE analysis (**new Fig. EV1E, F**). Under all conditions tested, mitotic samples consistently exhibited enhanced supercomplex assembly compared to interphase samples. These results confirm that the mitotic enrichment of respirasomes is a robust biological phenomenon and not an artifact of specific sample preparation conditions.

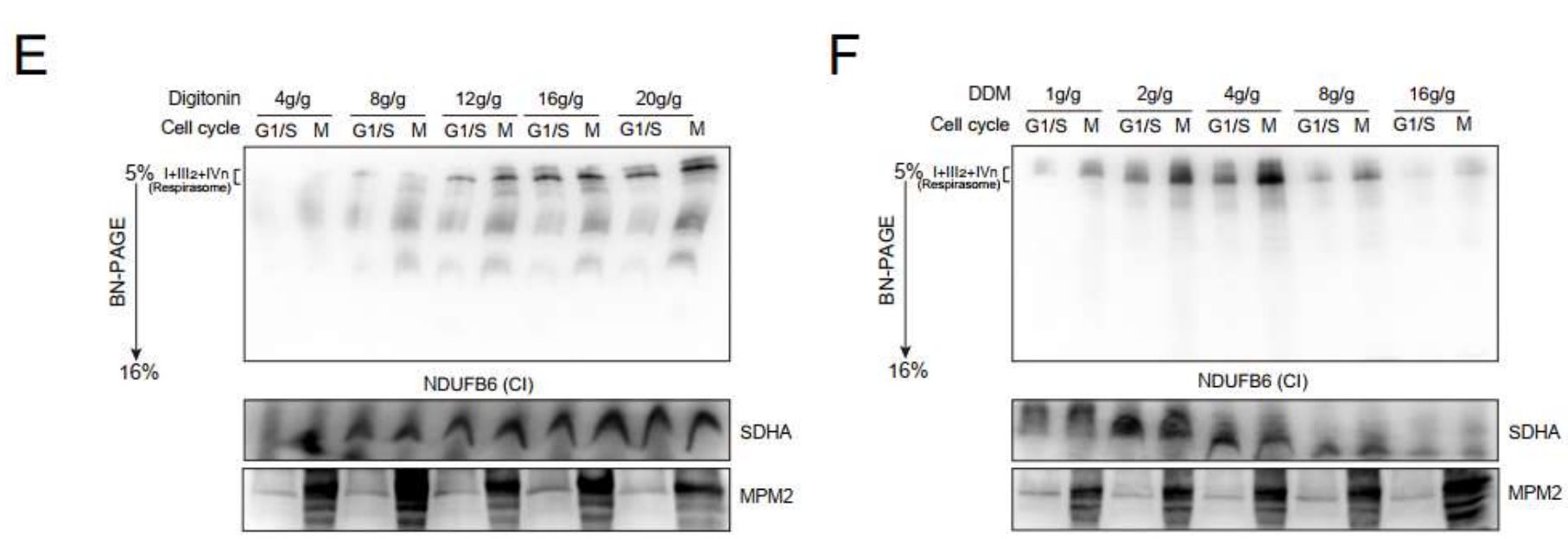

In addition, the assignment of individual BN bands to specific CI assembly intermediates or modules (PP-a, PP-b, PD-a, etc.) is based on single antibodies and schematic models; there is no rigorous mapping using multiple subunits per module, 2D BN/SDS or known assembly

factor mutants. As a result, the narrative of a "hierarchical, modular assembly" in mitosis feels considerably more precise than what the band resolution and validation actually allow.

***Response*:** We thank the reviewer for this critical feedback. To address the concern that the assignment of BN bands to specific CI assembly intermediates or modules is insufficiently validated, we have performed two complementary orthogonal analyses.

First, we carried out advanced complexome profiling by slicing entire BN-PAGE lanes into 13 fractions for LC-MS/MS analysis, enabling continuous migration profiling of all CI subunits (**Fig. 2B**).

Second, we conducted 2D BN/SDS-PAGE followed by immunoblotting using two antibodies per module to map the distribution of CI modules (N, Q, PP-a, PP-b, PD-a, PD-b) in G1/S versus mitotic cells, with or without NDUFA3 knockdown (**Fig. 2C**).

Our results demonstrate that NDUFA3 depletion specifically arrests CI assembly at the PP-a module (100–500 kDa) during mitosis, as evidenced by accumulation of PP-b, PD-a, and Q intermediates, accompanied by failed assembly of downstream modules (PD-b, N), as well as accumulation of $CIII_2$/CIV. These data provide a more rigorous mapping of modular assembly intermediates than was previously available.

In addition, in response to the reviewer's concern regarding overinterpretation, we have revised the manuscript text to remove the term "hierarchical" when describing the assembly process.

We believe these revisions address the reviewer's concerns and provide a more substantiated foundation for our conclusions.

3. The mechanistic attribution of the observed phenotypes specifically to "respirasome disassembly" is not entirely convincing and, in my opinion, underestimates the contribution of more general CI dysfunction and mitochondrial stress. NDUFA3 is an accessory CI subunit required for early CI assembly; its depletion predictably disrupts CI integrity, reduces CI activity and lowers the abundance of multiple CI components. Many of the downstream readouts, like ROS accumulation, mitochondrial depolarization, cristae changes, are exactly what one would expect from a generic CI deficiency and impaired electron flow, regardless of the precise supercomplex architecture. The attempt to distinguish "respirasome-specific" effects via SCAF1 knockdown is helpful but not fully conclusive, as SCAF1 manipulations also have broader effects on CIII/CIV organization and overall respiration, and the data provided do not exhaustively document total CI and other complexes in this setting.

Response: We thank the reviewer for raising this important mechanistic concern. To rigorously distinguish respirasome-specific effects from general Complex I dysfunction and mitochondrial stress, we have employed multiple orthogonal approaches.

First, we examined SCAF1 knockdown, which disrupts respirasome assembly without affecting the expression levels of individual CI, CIII, or CIV complexes (**Fig. EV5E**). This indicates that the integrity of individual complexes remains intact despite the loss of supercomplex organization.

Second, we generated UQCRC1 knockdown cells complemented with an shRNA-resistant UQCRC1$^{DEL\text{-}AED}$ mutant, which specifically disrupts the CI–$CIII_2$ interaction (PMID: 37633273). BN-PAGE confirmed selective respirasome disassembly in these cells without compromising the assembly of individual complexes (**Fig. EV5H, I**). These cells phenocopied the mitochondrial and mitotic defects observed upon NDUFA3 knockdown, and importantly, these defects were rescued by treatment with the mitochondrial-targeted antioxidant SKQ1 (**Fig. EV5J**).

Third, as a pharmacological control, we treated cells with rotenone or antimycin A, which inhibit CI or CIII activity, respectively, without disrupting supercomplex assembly. While these treatments elevated ROS and reduced mitochondrial membrane potential, they did not recapitulate the chromosome segregation defects seen upon NDUFA3 depletion (**Fig. EV5K–M**).

Taken together, these complementary approaches allow us to convincingly distinguish the phenotypic consequences of respirasome disassembly from those of general CI deficiency. We greatly appreciate the reviewer's rigorous critique, which has substantially strengthened the mechanistic conclusions of our study.

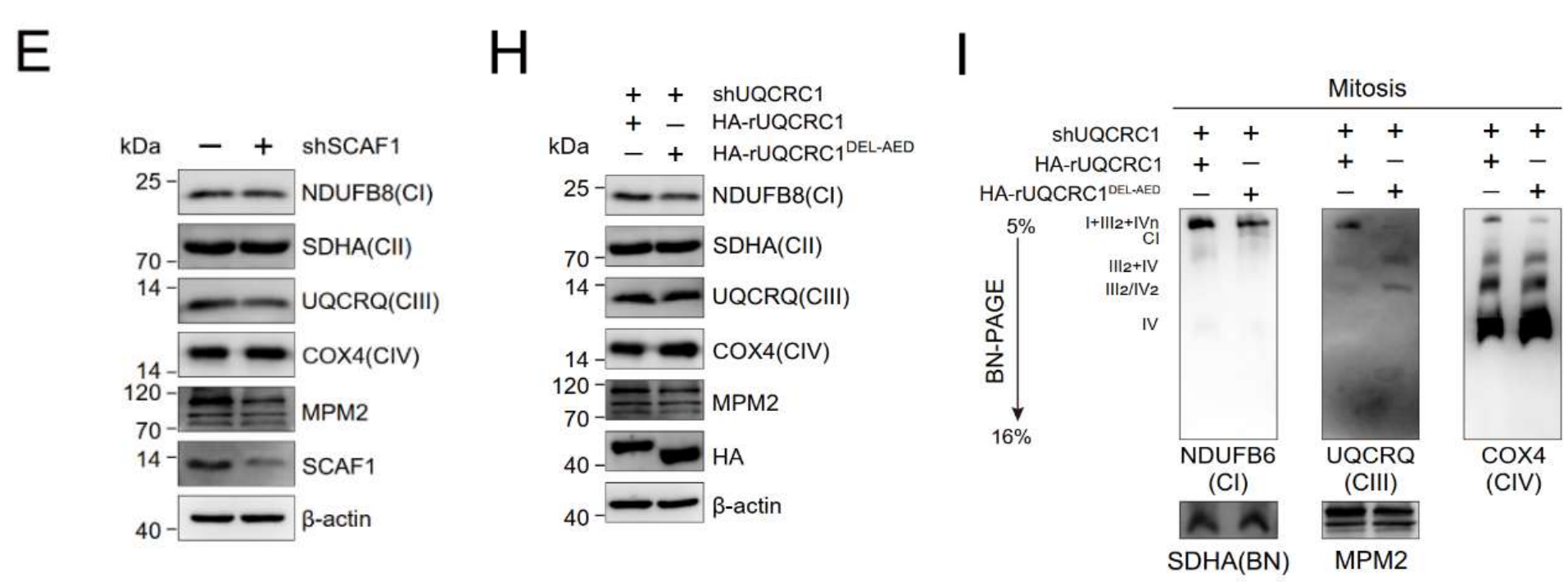

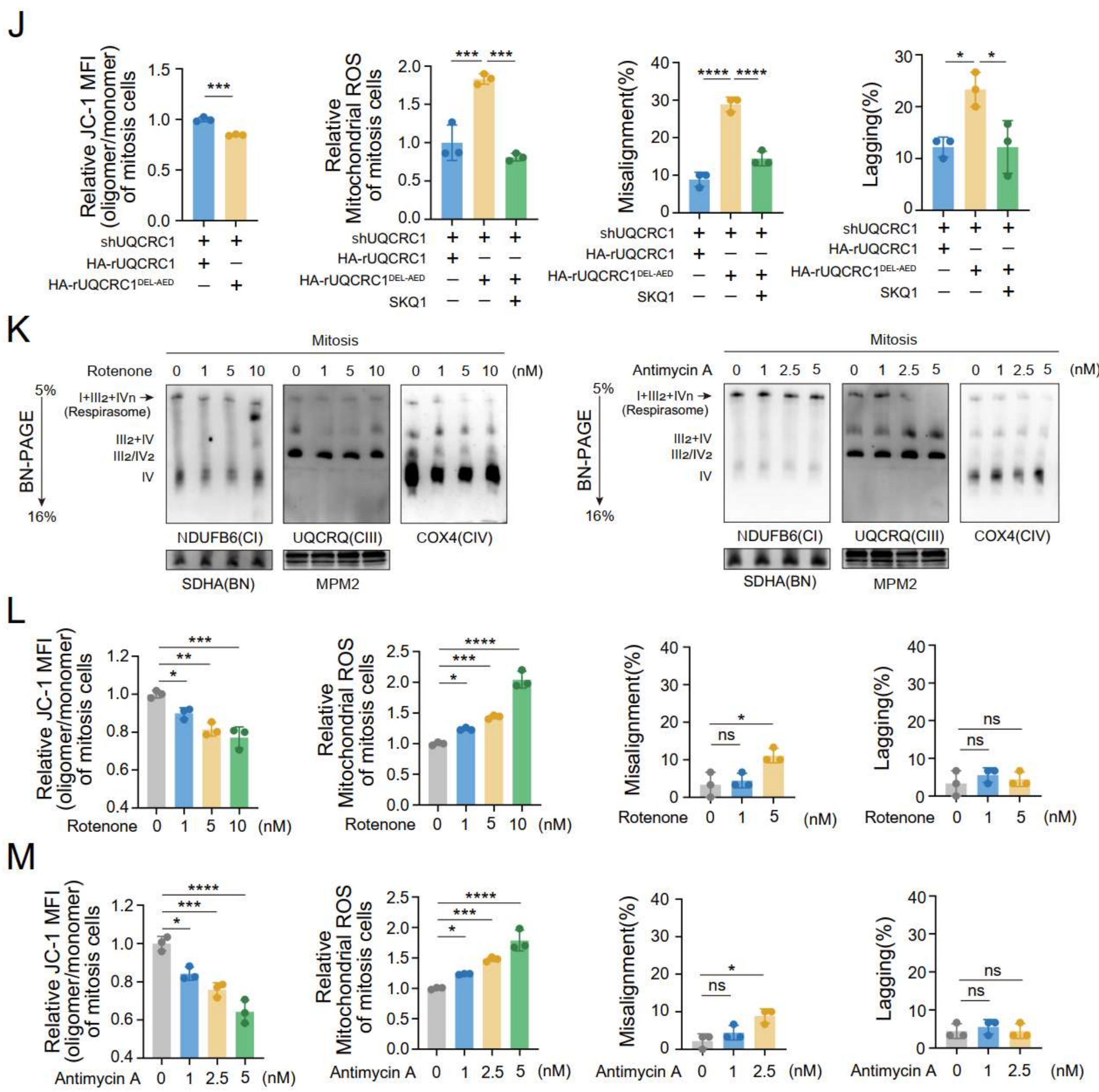

Furthermore, the rescue strategy rests almost entirely on NAC, a very non-specific antioxidant and thiol donor; without additional genetic or pharmacological approaches (e.g. mitochondria-targeted ROS scavengers, partial CI inhibition in the absence of supercomplex disruption...), the causal chain from respirasome organization to ROS, Aurora inhibition and chromosome mis-segregation remains more correlative than the text suggests.

Response**:** We thank the reviewer for raising this important methodological concern. To address the issue that our previous rescue experiments relied primarily on NAC, a relatively non-specific antioxidant and thiol donor, we have now employed two independent mitochondria-targeted ROS scavengers, SKQ1 and MitoQ, in NDUFA3 knockdown cells. Treatment with either SKQ1 or MitoQ effectively alleviated ROS elevation, restored Aurora kinase activity, and reduced chromosome segregation errors in NDUFA3 KD cells (**new Fig. EV5B–D**). These results provide stronger, more specific evidence supporting the causal

relationship from respirasome disassembly to mitotic ROS accumulation and subsequent Aurora kinase inhibition.

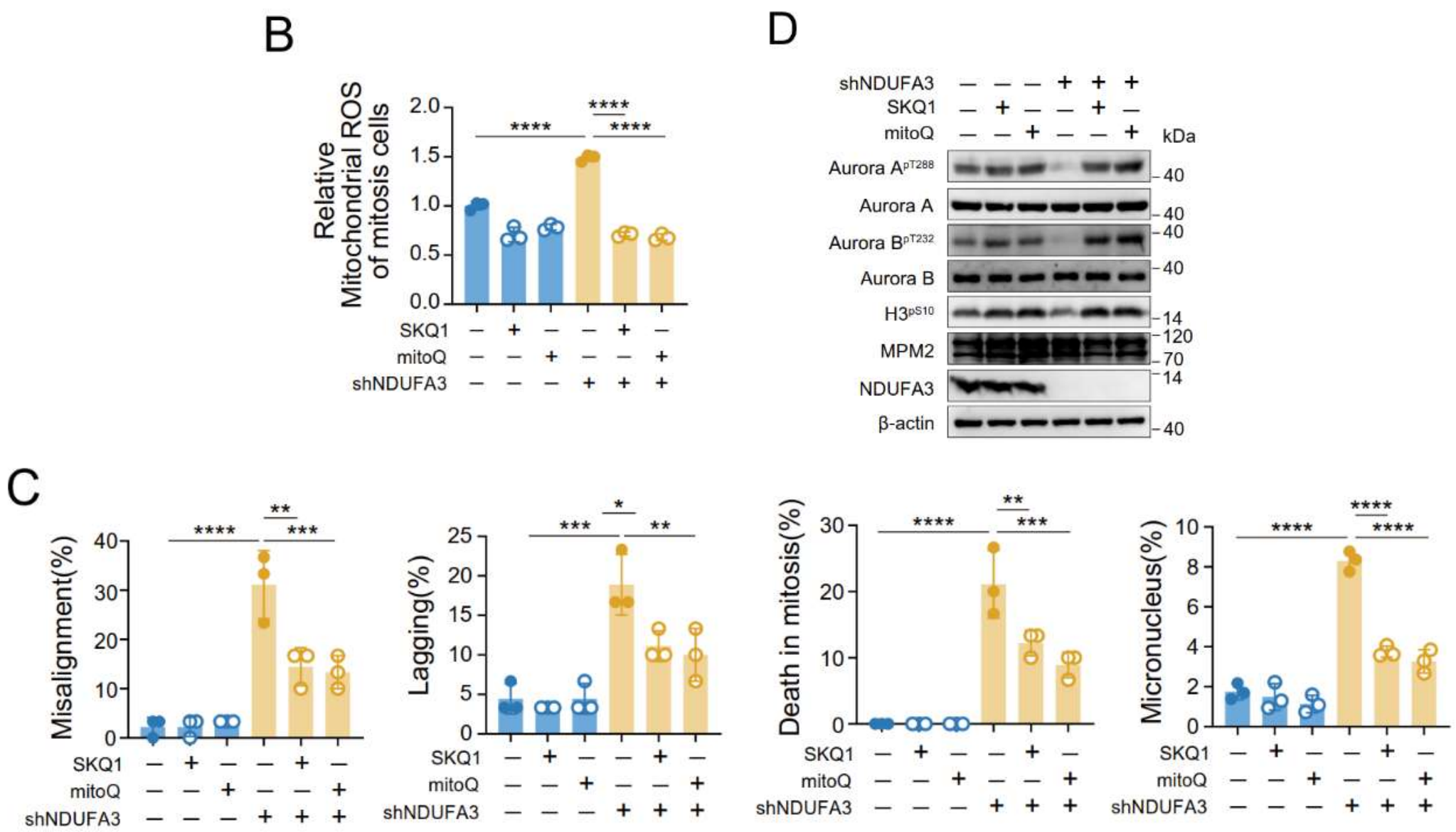

4. I am concerned about the way statistics and controls are handled across multiple figures, which affects my confidence in several of the quantitative claims. Many panels summarize complex biological phenomena (e.g. mitotic phenotypes, ROS, ΔΨm, NADPH, cristae morphology, organoid size, tumor growth) using simple two-group comparisons, often with very small numbers of biological replicates and without clear information on how sample size was determined, whether data distribution and variance assumptions were checked, or whether any correction for multiple testing was applied despite the large number of endpoints. This is particularly important for the in vivo tumor growth curves, where repeated measurements over time appear to be treated essentially as independent time points instead of being analyzed with an appropriate longitudinal approach or a global test. In addition, in several panels, controls are arbitrarily set to 1.0 and all experimental conditions are plotted relative to this baseline, effectively removing the variation of the control group from the statistical assessment and making it difficult to evaluate the true effect size and variability. This practice can artificially inflate apparent significance and obscures how noisy the control measurements actually are. I would strongly recommend re-plotting and re-analyzing the key datasets with absolute values, explicit reporting of n (biological vs technical), and more appropriate statistics given the number of comparisons and the repeated-measures nature of some experiments.

***Response*:** We thank the reviewer for this thorough and important critique regarding statistics and data presentation. In response, we have comprehensively revised our statistical analysis

and data presentation across all figures, as detailed below.

1. **Biological versus technical replicates**: All figure legends now clearly distinguish between biological and technical replicates, and the number of independent biological replicates (n) is explicitly stated for each experiment.
2. **Longitudinal data** (e.g., tumor growth curves): Tumor growth curves have been re-analyzed using a mixed-effects model for repeated measures, with appropriate correction for within-subject correlations over time, rather than treating each time point as independent.
3. **Multiple testing correction**: For analyses involving multiple endpoints, we have applied appropriate multiple testing corrections (e.g., Tukey's multiple comparisons test), and the correction method is now indicated in the respective figure legends.
4. **Control normalization**: We have removed the practice of arbitrarily setting control values to 1.0. All key datasets have been re-plotted using absolute values, and the variation in control measurements is now fully visible. Statistical comparisons are performed on absolute values, not normalized ratios.
5. **Statistical assumptions**: For each comparison, we have verified data distribution and variance assumptions (e.g., normality tests, equality of variances) and reported the results in the figure legends. Appropriate parametric or non-parametric tests are used accordingly.

We appreciate the reviewer's rigorous assessment, which has substantially improved the statistical integrity and transparency of our study.

5. Finally, I feel the conceptual narrative tends to overreach what the current dataset can firmly support. Most of the in-depth mechanistic work is carried out in two cancer cell lines, with additional models used only for selected readouts, yet the discussion generalizes to "cancer cells" and "tumor progression" in a broad sense.

***Response*:** We thank the reviewer for this thoughtful critique. In response, we have carefully revised the manuscript to ensure that our conclusions do not overreach the experimental scope. Specifically, our primary mechanistic findings are derived from HeLa and MDA-MB-468 cells, with additional validation in T47D and MCF7 cells. Accordingly, we have replaced overly broad terms such as "cancer cells" and "tumor progression" with more precise language, referring instead to "the tested cervical and breast cancer cell lines" and "proliferation/mitotic progression in vitro." We believe these modifications appropriately contextualize our findings and avoid overgeneralization. We appreciate the reviewer's careful reading, which has helped us improve the precision and rigor of our language.

In highly glycolytic cells, total ATP is maintained through glycolytic compensation, and the authors do not test conditions in which mitochondrial ATP becomes limiting (e.g. low glucose, galactose) or in which cells are truly OXPHOS-dependent, which would be important to underpin statements about respirasome assembly being "essential" for mitotic progression.

***Response*:** We thank the reviewer for this important metabolic insight. To directly address whether the requirement for respirasome assembly in mitotic progression holds under OXPHOS-dependent conditions, we performed the following experiments. First, we cultured NDUFA3 knockdown cells in galactose-containing medium to enforce OXPHOS dependence (PMID: 31023583). Under this condition, NDUFA3 depletion led to ATP depletion, exacerbated mitochondrial dysfunction, and more severe mitotic defects (**Fig. EV4C, E, F and EV5A**). Second, we noted that MDA-MB-468 cells, which are inherently more OXPHOS-dependent than HeLa cells, already exhibited significant ATP depletion and marked mitochondrial dysfunction upon NDUFA3 knockdown even in standard glucose medium (**Fig. EV4D, G, H**). Collectively, these results demonstrate that respirasome assembly becomes critically important when mitochondrial ATP production is limiting. We thank the reviewer for this thoughtful suggestion, which has substantially strengthened the metabolic rigor of our conclusions.

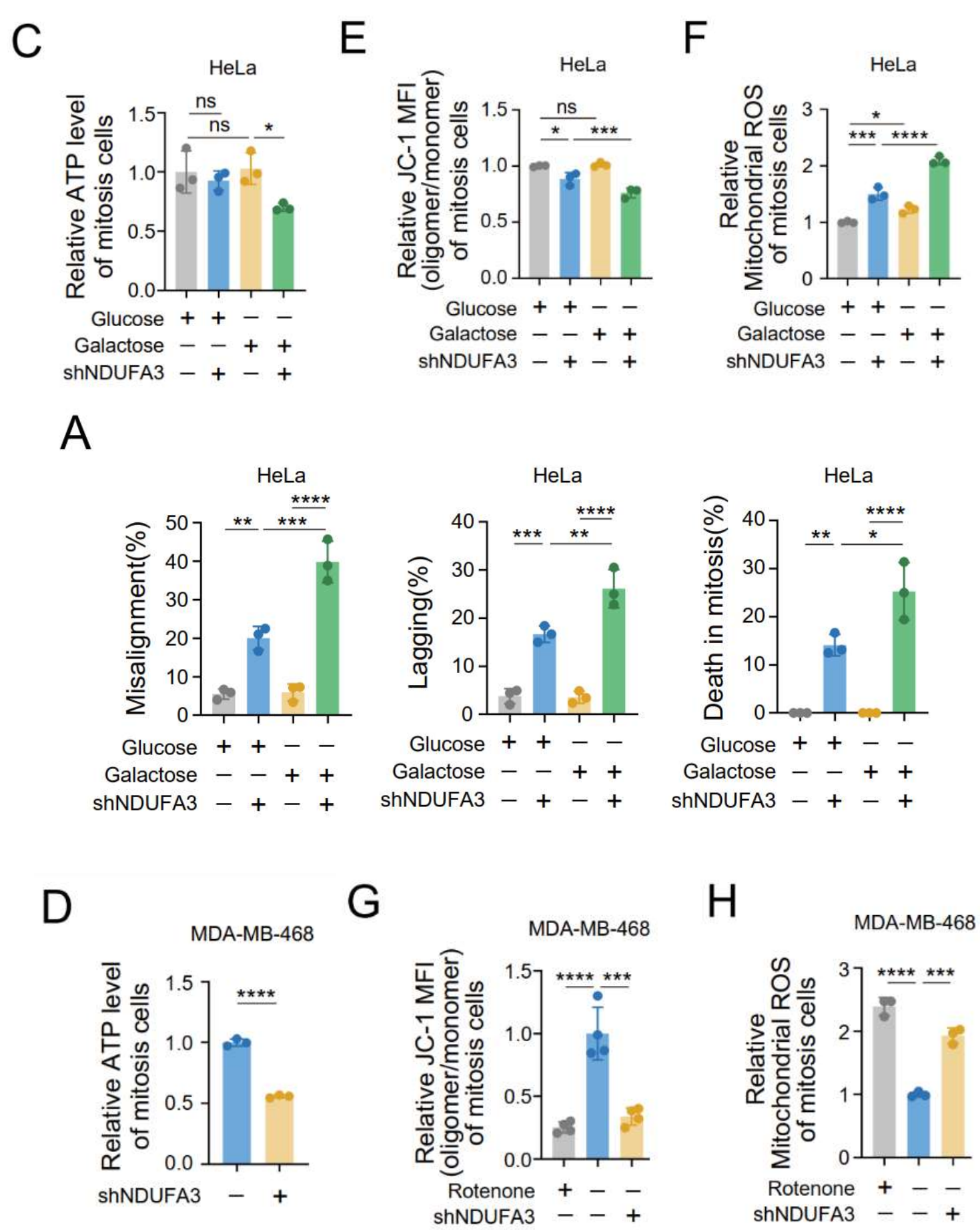

The organoid and in vivo data, although interesting, are based on relatively small cohorts and show partial effects that can have multiple explanations in complex tumor ecosystems; the partial rescue by NAC in vivo is suggestive but not definitive. Taken together, I would encourage the authors to either temper their claims, framing this as an important example of mitotic mitochondrial remodeling in specific cancer contexts, or to provide additional data that more rigorously demonstrate that mitotic respirasome assembly is a central, targetable driver of chromosome stability and tumor growth across models.

Response**:** We thank the reviewer for this thoughtful feedback. In response, we have revised the manuscript to temper our claims appropriately. Specifically, mitotic respirasome assembly is now presented as an important example of mitotic mitochondrial remodeling in specific cancer contexts (e.g., the cervical and breast cancer cell lines examined in this study), rather than as a universal driver across all cancer types or tumor models. We acknowledge the preliminary nature of our organoid and in vivo experiments, including the limited cohort sizes, and we have explicitly noted this limitation in the revised discussion. The partial rescue observed with NAC in vivo is now described as suggestive rather than definitive, and we have added a statement emphasizing the need for future validation in larger cohorts and additional models. We appreciate the reviewer's constructive guidance, which has helped us better contextualize our findings.

References:

1. Rath, S. et al. MitoCarta3.0: an updated mitochondrial proteome now with sub-organelle localization and pathway annotations. *Nucleic Acids Res* **49**, D1541-D1547 (2021). PMID: 33174596
2. Tanenbaum, M.E., Stern-Ginossar, N., Weissman, J.S. & Vale, R.D. Regulation of mRNA translation during mitosis. *eLife* **4**, e07957 (2015). PMID: 26305499
3. Ly, T. et al. Proteomic analysis of cell cycle progression in asynchronous cultures, including mitotic subphases, using PRIMMUS. *eLife* **6**, e27574 (2017). PMID: 29052541
4. Mahdessian, D. et al. Spatiotemporal dissection of the cell cycle with single-cell proteogenomics. *Nature* **590**, 649-654 (2021). PMID: 33627808
5. Balsa, E. et al. ER and Nutrient Stress Promote Assembly of Respiratory Chain Supercomplexes through the PERK-eIF2α Axis. *Mol Cell* **74**, 877-890 (2019). PMID: 31023583
6. Greggio, C. et al. Enhanced Respiratory Chain Supercomplex Formation in Response to Exercise in Human Skeletal Muscle. *Cell Metab* **25**, 301-311 (2017). PMID: 27916530
7. Hollinshead, K.ER. et al. Respiratory Supercomplexes Promote Mitochondrial Efficiency and Growth in Severely Hypoxic Pancreatic Cancer. *Cell Rep* **33**, 108231 (2020). PMID:

33027658

8. Lopez-Fabuel, I. et al. Complex I assembly into supercomplexes determines differential mitochondrial ROS production in neurons and astrocytes. *Proc Natl Acad Sci U S A* **113**, 108231 (2016). PMID: 27799543
9. Gonzalez-Franquesa, A. et al. Mass-spectrometry-based proteomics reveals mitochondrial supercomplexome plasticity. *Cell Rep* **35**, 109180 (2021). PMID: 34038727
10. Milenkovic, D. et al. Preserved respiratory chain capacity and physiology in mice with profoundly reduced levels of mitochondrial respirasomes. *Cell Metab* **35**, 1-15 (2023). PMID: 37633273

1st Revision - Editorial Decision 20th Aug 2026

Dear Dr. Guo

Thank you for the submission of your revised manuscript to EMBO reports. We have now received the full set of referee reports and cross-comments that is copied below.

As you can see, the referees find that the study is significantly improved during revision and recommend publication. Referee 3 has remaining concerns, but Referee 1 and Referee 2 feel that these can be addressed textually without additional experimentation. Before I can accept the manuscript, I need you to address some minor points below:

- As a standard procedure, we edit the abstract of manuscripts to make them more accessible to a general readership. Please find the suggested version below my signature.

- Please rename the "Competing interests" to Disclosure and Competing Interests Statement.

- Figure legends should be placed at the end of the manuscript, after the References.

- Please remove the instructions and example table from the Reagents and Tools Table.

- CRediT has replaced the traditional author contributions section because it offers a systematic machine readable author contributions format that allows for more effective research assessment. Please remove the Author Contributions section from the manuscript and use the free text boxes beneath each contributing author's name in our online systems to add specific details on the author's contribution. More information is available in our guide to authors.

- Only one funder and two grant numbers are entered in our system. We need all grant numbers and all funders to be provided as separate entries in the system. The comment box should not be used.

- We do not allow multiple files/pages for a figure. Please split Figure EV5 into two EV figures.

- Please incorporate more of the mouse housing conditions included in the Author Checklist in the Methods section of the manuscript.

- Please note that the specific URLs for PXD078881, PXD078879, PXD079047 datasets need to be provided in the data availability section.

- Our production/data editors have asked you to clarify several points in the figure legends (see below). Please incorporate these changes in the manuscript and return the revised file with tracked changes with your final manuscript submission.
- Please note that the exact p values are not provided in the legends of figures 4C, D, E, F, G, H, I; 5B, 6C, D; EV1 G, EV4 D, F, G, H; EV5 A, B, C, J, L, M.
- Please note that the dashed borders are not defined in the legend of figures EV1 G. This needs to be rectified.

- Please note that EMBO press papers are accompanied online by:
A) a short (2 sentences) summary of the findings and their significance,
B) 2-5 short bullet points highlighting the key results, and
C) a synopsis image in .jpg or .png format that is exactly 550 pixels wide and 300 pixels high. Please note that the text needs to be legible at the final size. Please upload this information along with your revised manuscript (the text for A and B should be provided in one separate Word file uploaded as Cover Art/Synopsis Image).

We look forward to seeing a final version of your manuscript as soon as possible.

With kind regards,

Kurt Weir
Editor
EMBO Reports

As cells progress from interphase into mitosis, fluctuating metabolic demands coincide with mitochondrial fission. However, the mechanisms by which mitochondria coordinate morphological changes and metabolic adjustments during mitosis remain poorly understood. Using complexome profiling, we show that assembly of the mitochondrial respirasome, a supercomplex comprising electron transport chain complexes I, III, and IV, is markedly enhanced during mitosis in HeLa and MDA-MB-468 cancer cells. Mechanistically, the upregulation of specific CI subunits, including NDUFA3, drives the modular assembly of the respirasome in mitosis. We further demonstrate that CDK1 promotes the translation of NDUFA3 to enable respirasome formation. Disruption of this process impairs mitochondrial integrity, energy production, and redox homeostasis, leading to ROS accumulation that

triggers mitotic cell death and chromosome segregation defects across multiple models. Importantly, inhibiting mitotic respirasome assembly induces chromosome mis-segregation and suppresses tumor growth in vivo. Our findings reveal respirasome assembly as a key mechanism coordinating mitochondrial fission with metabolic adaptation to support cell division and tumor proliferation in these cancer cell contexts.

Referee #1:

The authors have carefully addressed all suggestions and have further strengthened what was already a compelling manuscript. I am satisfied with the revisions and recommend publication in EMBO Reports.

Referee #2:

In this revised version, the authors have addressed very well all the points I raised in the initial submission. They have performed further experiments and textual adjustments, and thereby improved the manuscript significantly.

Referee #3:

Wang et al. report that complex I subunits and high-molecular-weight respiratory assemblies increase during mitosis in cancer cells. They propose that CDK1-dependent translational induction of NDUFA3 promotes respirasome assembly, limits ROS, preserves Aurora kinase activity, and thereby supports chromosome segregation and tumour growth. The manuscript contains a genuine and potentially important observation. The mitotic increase in NDUFA3 is supported by independent ribosome-profiling datasets, and the dependence on CDK1 is consistent with the broader role of CDK1 in mitotic translational control. The finding that depletion of NDUFA3 and other PP-a module subunits impairs complex I-containing assemblies is also consistent with established complex I assembly biology. The strongest conclusion is therefore that mitosis is accompanied by regulated remodelling of complex I (CI) subunit abundance, with consequences for mitochondrial and redox homeostasis in cancer cells. However, the manuscript builds a broader mechanistic narrative that is not fully supported by the data. In particular, it does not establish that a structurally defined respirasome is specifically increased, that loss of this assembly directly increases CI-derived ROS, or that the phenomenon represents a universal requirement of normal cell division. The evidence instead raises the possibility that this is predominantly a tumour-cell adaptation.

Major concerns
1. Is the mechanism described here specific for tumour cells? Most experiments are performed in HeLa and MDA-MB-468 cells, both of which have high basal oxidative stress, marked aneuploidy, and pre-existing mitotic instability. In contrast, the mitotic increase in high-molecular-weight respiratory assemblies is reported to be substantially weaker in non-transformed RPE cells. This is arguably the most important observation in the manuscript, yet it is treated as a minor supplementary result. The weaker response in RPE cells directly challenges the claim that increased respiratory supercomplex assembly is a universal mechanism required for mitosis. Instead, tumour cells may have a greater dependence on increased CI assembly capacity during division because they already operate close to a threshold of redox and mitotic stress. Under these conditions, NDUFA3 depletion may exacerbate pre-existing vulnerabilities and thereby cause oxidative stress, reduced Aurora kinase activity, and chromosome-segregation defects.
The authors should fully characterise NDUFA3 abundance, high-molecular-weight CI assemblies, oxidative stress, Aurora kinase activity, and chromosome segregation after NDUFA3 depletion in RPE cells and preferably in additional non-transformed or primary models. They should also determine whether inhibition of this pathway selectively affects tumour cells while being tolerated by non-transformed proliferating cells.
A tumour-specific interpretation would not weaken the paper. It could provide a more compelling framework in which CDK1-dependent NDUFA3 translation represents a mitotic redox adaptation and potential metabolic vulnerability of cancer cells. The organoid and xenograft experiments are more naturally aligned with this interpretation than with a universal mechanism of cell division.

2. The structural and proteomic evidence does not support the terms "respirasome" or "complexome profiling". The manuscript repeatedly refers to a specific respirasome, generally understood as a defined CI+$CIII_2$+CIV assembly. However, the BN-PAGE bands are broad, and the proteomic analysis of three or thirteen gel fractions cannot reliably distinguish CI+$CIII_2$, CI+$CIII_2$+CIV_1, CI+$CIII_2$+CIV_2, or other co-migrating assemblies.
The data support changes in high-molecular-weight complex I-containing or CI/CIII/CIV-containing fractions, but not the precise stoichiometric complex implied by the term "respirasome". This distinction is important because functional claims concerning electron transfer, ROS suppression, and respiratory efficiency depend on the specific architecture of the assembly.
Similarly, analysis of three selected bands or thirteen broad fractions does not constitute conventional complexome profiling, which requires sufficiently dense fractionation of the complete BN-PAGE lane to reconstruct protein migration profiles and resolve assembly intermediates. The experiments should instead be described as proteomic analysis of excised BN-PAGE fractions unless correct higher-resolutioncomplexome profiling is performed. The authors should therefore either provide genuine high-resolution complexome or structural evidence, or revise the terminology throughout to "high-molecular-weight respiratory assemblies", "complex I-containing supercomplexes", or another formulation that accurately reflects the data.

3. The proposed ROS mechanism is not supported by either the biochemistry or the measurements. The manuscript argues that respirasome assembly suppresses CI-derived ROS and that NDUFA3 depletion increases ROS by disrupting this function. However, NDUFA3 depletion also reduces complex I activity and oxygen consumption. Reduced forward electron flow through CI would not by itself be expected to increase superoxide production from its major redox sites. The observed oxidative signal may instead result from broader mitochondrial dysfunction, including membrane depolarisation, altered NAD^+/NADH and NADPH balance, impaired antioxidant capacity, and compensatory metabolic changes. These effects are compatible with loss of CI function but do not demonstrate a specific ROS-suppressive role of physical association between complexes I and III. The rotenone, antimycin A, SCAF1, and UQCRC1ΔAED experiments do not cleanly separate supercomplex organisation from changes in respiratory-chain activity and redox balance. They therefore do not establish that loss of a particular respiratory assembly is the direct cause of CI-derived ROS.
The measurements themselves are also insufficiently specific for the mechanistic claims made. CM-H_2DCFDA reports a broad and poorly defined oxidative signal, while standard fluorescence-based MitoSOX measurements cannot distinguish the superoxide-specific product from non-specific oxidation products, or even mtDNA levels. The authors should supplement these experiments with more specific, preferably ratiometric and compartment-targeted approaches, such as mitochondrial HyPer7 or roGFP2-Orp1 for H_2O_2. Claims of mitochondrial superoxide production would require appropriate product separation or another validated method. Until the source and species of the oxidant are established, the conclusions should refer to oxidative stress or redox imbalance rather than specifically to complex I-derived superoxide.

4. The proposed mitosis-CDK1-NDUFA3 mechanism remains incomplete. The study shows that NDUFA3 translation and CI-containing assemblies increase during mitosis and are reduced by CDK1 inhibition. However, CDK1 inhibition globally disrupts mitosis and affects many components of the mitotic translational programme. The present data therefore establish CDK1 dependence but not a specific regulatory pathway acting directly on NDUFA3 mRNA.
To support a specific CDK1-NDUFA3 translational axis, the authors would need to identify the relevant CDK1 substrate, RNA-binding protein, translation factor, or cis-regulatory element within the NDUFA3 transcript. A reporter containing the NDUFA3 5′ UTR or other candidate regulatory regions could help determine whether the transcript itself confers mitosis- and CDK1-dependent translation.
The upstream reason for increased CI assembly during mitosis is also unresolved. The authors propose that mitochondrial fragmentation reduces respiratory efficiency and creates a compensatory demand for additional respiratory assemblies. This is plausible but remains untested. It is not shown that blocking mitotic fission prevents NDUFA3 induction or that inducing mitochondrial fragmentation outside mitosis is sufficient to activate the same response. The manuscript therefore identifies a mitosis-associated increase in NDUFA3 translation and CI assembly capacity, but it does not yet establish either the specific translational mechanism or the physiological trigger. Unless these relationships are tested directly, they should be presented as associations rather than as a complete mechanistic pathway.

Minor and technical concerns
- The Seahorse experiments should confirm that the analysed cells remain mitotic at the time of measurement. Mitotic cells collected by shake-off are replated and allowed to attach before the assay, during which some cells may complete mitosis and enter G1. A mitotic marker should therefore be assessed in the same population at the time of metabolic measurement.
- Because NDUFA8 and NDUFA13 are also increased during mitosis, their depletion should be tested to determine whether the phenotype is specific to NDUFA3 or reflects a broader requirement for PP-a module assembly.
- The in vivo NAC dose appears low relative to doses commonly used to alter systemic redox balance. The authors should justify the selected dose, perform a dose-response, or test an alternative antioxidant strategy.
- The title should also be revised. "Supports accurate chromosome segregation" would be clearer than "promotes chromosomal segregation", while "tumour growth" is more accurate than "tumour progression".

Cross-Comment

Referee 2

I think most of the major and minor concerns by reviewer #3 could be dealt by with textual changes. If any new experiment would be necessary, it is these from major point #1, ie replicating much of the analyses done in tumor cells in non-transformed RPE cells.

Referee 1

I appreciate Referee #3's thoughtful comments. In my view, most of the concerns raised relate to terminology, interpretation, and the scope of the conclusions, and could be addressed through appropriate textual revisions and clarification of the manuscript. If any additional experimental work were deemed necessary, the strongest case would be for further analysis in non-transformed cells as suggested under major point #1. However, I do not consider extensive new experimentation essential, and I remain supportive of publication following revision.

Point-by-point responses

We sincerely thank the Editor and Reviewers for their positive comments and constructive suggestions. Below, we provide detailed point-by-point responses (**in blue text**) to each concern raised.

Point-by-point response to referee's comments

Referee # 1:

The authors have carefully addressed all suggestions and have further strengthened what was already a compelling manuscript. I am satisfied with the revisions and recommend publication in EMBO Reports.

Cross-Comment：I appreciate Referee #3's thoughtful comments. In my view, most of the concerns raised relate to terminology, interpretation, and the scope of the conclusions, and could be addressed through appropriate textual revisions and clarification of the manuscript. If any additional experimental work were deemed necessary, the strongest case would be for further analysis in non-transformed cells as suggested under major point #1. However, I do not consider extensive new experimentation essential, and I remain supportive of publication following revision.

Response: We appreciate the reviewer's accurate summarization and positive feedback. We have revised the manuscript to clarify terminology, refine interpretation, and appropriately limit the scope of our conclusions. Regarding the suggestion for further analysis in non-transformed cells, we have addressed this limitation in the Discussion and confined our main conclusions to the tumor cell models used in this study.

Referee # 2：

In this revised version, the authors have addressed very well all the points I raised in the initial submission. They have performed further experiments and textual adjustments, and thereby improved the manuscript significantly.

Cross-Comment：I think most of the major and minor concerns by reviewer #3 could be dealt by with textual changes. If any new experiment would be necessary, it is these from major point #1, ie replicating much of the analyses done in tumor cells in non-transformed RPE cells.

Response: We thank the reviewer for the insightful comments and suggestions. We have addressed the textual concerns as recommended. Regarding the suggestion to replicate analyses

in non-transformed RPE cells, we have revised the Discussion to explicitly address the limitations of our findings and have confined our main conclusions to the tumor cell models used in this study.

Referee # 3：

Wang et al. report that complex I subunits and high-molecular-weight respiratory assemblies increase during mitosis in cancer cells. They propose that CDK1-dependent translational induction of NDUFA3 promotes respirasome assembly, limits ROS, preserves Aurora kinase activity, and thereby supports chromosome segregation and tumour growth. The manuscript contains a genuine and potentially important observation. The mitotic increase in NDUFA3 is supported by independent ribosome-profiling datasets, and the dependence on CDK1 is consistent with the broader role of CDK1 in mitotic translational control. The finding that depletion of NDUFA3 and other PP-a module subunits impairs complex I-containing assemblies is also consistent with established complex I assembly biology. The strongest conclusion is therefore that mitosis is accompanied by regulated remodelling of complex I (CI) subunit abundance, with consequences for mitochondrial and redox homeostasis in cancer cells. However, the manuscript builds a broader mechanistic narrative that is not fully supported by the data. In particular, it does not establish that a structurally defined respirasome is specifically increased, that loss of this assembly directly increases CI-derived ROS, or that the phenomenon represents a universal requirement of normal cell division. The evidence instead raises the possibility that this is predominantly a tumour-cell adaptation.

Major concerns

1. Is the mechanism described here specific for tumour cells? Most experiments are performed in HeLa and MDA-MB-468 cells, both of which have high basal oxidative stress, marked aneuploidy, and pre-existing mitotic instability. In contrast, the mitotic increase in high-molecular-weight respiratory assemblies is reported to be substantially weaker in non-transformed RPE cells. This is arguably the most important observation in the manuscript, yet it is treated as a minor supplementary result. The weaker response in RPE cells directly challenges the claim that increased respiratory supercomplex assembly is a universal mechanism required for mitosis. Instead, tumour cells may have a greater dependence on increased CI assembly capacity during division because they already operate close to a threshold of redox and mitotic stress. Under these conditions, NDUFA3 depletion may exacerbate pre-existing vulnerabilities and thereby cause oxidative stress, reduced Aurora kinase activity, and chromosome-segregation defects.

The authors should fully characterise NDUFA3 abundance, high-molecular-weight CI assemblies, oxidative stress, Aurora kinase activity, and chromosome segregation after NDUFA3 depletion in RPE cells and preferably in additional non-transformed or primary models. They should also determine whether inhibition of this pathway selectively affects tumour cells while being tolerated by non-transformed proliferating cells.

A tumour-specific interpretation would not weaken the paper. It could provide a more compelling framework in which CDK1-dependent NDUFA3 translation represents a mitotic redox adaptation and potential metabolic vulnerability of cancer cells. The organoid and xenograft experiments are more naturally aligned with this interpretation than with a universal mechanism of cell division.

Response: We thank the reviewer for the very constructive criticisms and suggestions. We have revised the Discussion to explicitly address the weaker phenotype in non-transformed RPE cells and to emphasize the tumour-specific interpretation of our findings, framing CDK1-dependent NDUFA3 translation as a mitotic redox adaptation and potential metabolic vulnerability in cancer cells rather than a universal requirement for cell division. We have also confined our main conclusions to the tumor cell models used in this study.

2. The structural and proteomic evidence does not support the terms "respirasome" or "complexome profiling". The manuscript repeatedly refers to a specific respirasome, generally understood as a defined CI+$CIII_2$+CIV assembly. However, the BN-PAGE bands are broad, and the proteomic analysis of three or thirteen gel fractions cannot reliably distinguish CI+$CIII_2$, CI+$CIII_2$+CIV_1, CI+$CIII_2$+CIV_2, or other co-migrating assemblies.

The data support changes in high-molecular-weight complex I-containing or CI/CIII/CIV-containing fractions, but not the precise stoichiometric complex implied by the term "respirasome". This distinction is important because functional claims concerning electron transfer, ROS suppression, and respiratory efficiency depend on the specific architecture of the assembly.

Similarly, analysis of three selected bands or thirteen broad fractions does not constitute conventional complexome profiling, which requires sufficiently dense fractionation of the complete BN-PAGE lane to reconstruct protein migration profiles and resolve assembly intermediates. The experiments should instead be described as proteomic analysis of excised BN-PAGE fractions unless correct higher-resolution complexome profiling is performed. The authors should therefore either provide genuine high-resolution complexome or structural evidence, or revise the terminology throughout to "high-molecular-weight respiratory assemblies", "complex I-containing supercomplexes", or another formulation that accurately reflects the data.

Response: We thank the reviewer for the very constructive criticisms and suggestions. We have revised the terminology throughout the manuscript to more accurately reflect the data. Specifically, "complexome profiling" has been replaced with "proteomic analysis of BN-PAGE fractions" to more accurately describe our approach. We have replaced "respirasome" with "mitochondrial respiratory supercomplexes", with explicit clarification that our data do not resolve precise stoichiometry throughout the manuscript.

3. The proposed ROS mechanism is not supported by either the biochemistry or the measurements. The manuscript argues that respirasome assembly suppresses CI-derived ROS and that NDUFA3 depletion increases ROS by disrupting this function. However, NDUFA3 depletion also reduces complex I activity and oxygen consumption. Reduced forward electron flow through CI would not by itself be expected to increase superoxide production from its major redox sites. The observed oxidative signal may instead result from broader mitochondrial dysfunction, including membrane depolarisation, altered NAD^+/NADH and NADPH balance, impaired antioxidant capacity, and compensatory metabolic changes. These effects are compatible with loss of CI function but do not demonstrate a specific ROS-suppressive role of physical association between complexes I and III. The rotenone, antimycin A, SCAF1, and UQCRC1ΔAED experiments do not cleanly separate supercomplex organisation from changes in respiratory-chain activity and redox balance. They therefore do not establish that loss of a particular respiratory assembly is the direct cause of CI-derived ROS.

The measurements themselves are also insufficiently specific for the mechanistic claims made. CM-H_2DCFDA reports a broad and poorly defined oxidative signal, while standard fluorescence-based MitoSOX measurements cannot distinguish the superoxide-specific product from non-specific oxidation products, or even mtDNA levels. The authors should supplement these experiments with more specific, preferably ratiometric and compartment-targeted approaches, such as mitochondrial HyPer7 or roGFP2-Orp1 for H_2O_2. Claims of mitochondrial superoxide production would require appropriate product separation or another validated method. Until the source and species of the oxidant are established, the conclusions should refer to oxidative stress or redox imbalance rather than specifically to complex I-derived superoxide.

Response: We thank the reviewer for the very constructive criticisms and suggestions. We have revised the relevant text to refer to "oxidative stress" or "redox imbalance" rather than specifically to complex I-derived superoxide, and have tempered our mechanistic claims accordingly.

4. The proposed mitosis-CDK1-NDUFA3 mechanism remains incomplete. The study shows that NDUFA3 translation and CI-containing assemblies increase during mitosis and are reduced

by CDK1 inhibition. However, CDK1 inhibition globally disrupts mitosis and affects many components of the mitotic translational programme. The present data therefore establish CDK1 dependence but not a specific regulatory pathway acting directly on NDUFA3 mRNA.

To support a specific CDK1-NDUFA3 translational axis, the authors would need to identify the relevant CDK1 substrate, RNA-binding protein, translation factor, or cis-regulatory element within the NDUFA3 transcript. A reporter containing the NDUFA3 5′ UTR or other candidate regulatory regions could help determine whether the transcript itself confers mitosis- and CDK1-dependent translation.

The upstream reason for increased CI assembly during mitosis is also unresolved. The authors propose that mitochondrial fragmentation reduces respiratory efficiency and creates a compensatory demand for additional respiratory assemblies. This is plausible but remains untested. It is not shown that blocking mitotic fission prevents NDUFA3 induction or that inducing mitochondrial fragmentation outside mitosis is sufficient to activate the same response. The manuscript therefore identifies a mitosis-associated increase in NDUFA3 translation and CI assembly capacity, but it does not yet establish either the specific translational mechanism or the physiological trigger. Unless these relationships are tested directly, they should be presented as associations rather than as a complete mechanistic pathway.

Response: We thank the reviewer for the very constructive criticisms and suggestions. We have revised the manuscript to present these relationships as associations rather than as a complete mechanistic pathway. We have also expanded our Discussion to address the limitations of our study and have toned down our language regarding the CDK1-NDUFA3 axis throughout.

Minor and technical concerns

5. The Seahorse experiments should confirm that the analysed cells remain mitotic at the time of measurement. Mitotic cells collected by shake-off are replated and allowed to attach before the assay, during which some cells may complete mitosis and enter G1. A mitotic marker should therefore be assessed in the same population at the time of metabolic measurement.

Response: We thank the reviewer for the constructive suggestion. We have clarified in the Materials and Methods that mitotic cells were replated in medium containing 10 μM MG132 to maintain mitotic arrest during downstream assays, including Seahorse measurements.

6. Because NDUFA8 and NDUFA13 are also increased during mitosis, their depletion should be tested to determine whether the phenotype is specific to NDUFA3 or reflects a broader requirement for PP-a module assembly.

Response: We thank the reviewer for the constructive suggestion. We have added a statement

in the Discussion noting that NDUFA8 and NDUFA13 also increase during mitosis, suggesting a broader demand for P_P-a module subunits, while acknowledging that their functional roles in mitotic supercomplex assembly warrant future investigation.

7. The in vivo NAC dose appears low relative to doses commonly used to alter systemic redox balance. The authors should justify the selected dose, perform a dose-response, or test an alternative antioxidant strategy.

Response: We thank the reviewer for the constructive suggestion. We have added a justification for the NAC dose in the Materials and Methods, noting that it was selected based on protocols established in our previous work (Cheng *et al*., 2023), which demonstrated effective antioxidant activity in murine xenograft models.

8. The title should also be revised. "Supports accurate chromosome segregation" would be clearer than "promotes chromosomal segregation", while "tumour growth" is more accurate than "tumour progression".

Response: We thank the reviewer for the suggestion. We have revised the title to "Mitotic Assembly of Respiratory Supercomplexes Ensures Accurate Cell Division and Fuels Tumor Growth".

Dr. Jing Guo
University of Science and Techology of China
Hefei 230027
China

Dear Dr. Guo,

Thank you for the submission of your further revised manuscript to our editorial offices. Going through the manuscript and your further p-b-p-response, I consider the remaining concerns of referee #3 and the editorial requests as adequately addressed.

I am thus very pleased to accept your manuscript for publication in the next available issue of EMBO reports. Thank you for your contribution to our journal.

Your manuscript will be processed for publication by EMBO Press. It will be copy edited and you will receive page proofs prior to publication. Please note that you will be contacted by Springer Nature Author Services to complete licensing and payment information.

You may qualify for financial assistance for your publication charges - either via a Springer Nature fully open access agreement or an EMBO initiative. Check your eligibility: https://link.springer.com/journal/44319/how-to-publish-with-us

Should you be planning a Press Release on your article, please notify the production team via this form in order to coordinate publication and release dates: https://forms.cloud.microsoft/e/eH4A8JVP6g

If you have any questions, please do not hesitate to contact the Editorial Office. Thank you for your contribution to EMBO Reports.

Yours sincerely,

Achim Breiling
Senior Editor
EMBO Reports

>>> Please note that it is EMBO Reports policy for the transcript of the editorial process (containing referee reports and your response letter) to be published as an online supplement to each paper. If you do NOT want this, you will need to inform the Editorial Office via email immediately. More information is available here: https://link.springer.com/partners/embo-press/editorial-policies#Peer%20review

EMBO Press Author Checklist

Corresponding Author Name: Jing Guo, Zhenye Yang, Kaiguang Zhang, Weiwei Yang.	
Journal Submitted to: EMBO reports	
Manuscript Number: EMBOR-2026-63652	

USEFUL LINKS FOR COMPLETING THIS FORM
The EMBO Journal - Author Guidelines
EMBO Reports - Author Guidelines
Molecular Systems Biology - Author Guidelines
EMBO Molecular Medicine - Author Guidelines

Reporting Checklist for Life Science Articles (updated January
This checklist is adapted from Materials Design Analysis Reporting (MDAR) Checklist for Authors. MDAR establishes a minimum set of requirements in transparent reporting in the life sciences (see Statement of Task: 10.31222/osf.io/9sm4x). Please follow the journal's guidelines in preparing your manuscript.
Please note that a copy of this checklist will be published alongside your article.

Abridged guidelines for figures
1. Data
The data shown in figures should satisfy the following conditions:
- the data were obtained and processed according to the field's best practice and are presented to reflect the results of the experiments in an accurate and unbiased manner.
- ideally, figure panels should include only measurements that are directly comparable to each other and obtained with the same assay.
- plots include clearly labeled error bars for independent experiments and sample sizes. Unless justified, error bars should not be shown for technical
- if n<5, the individual data points from each experiment should be plotted. Any statistical test employed should be justified.
- Source Data should be included to report the data underlying figures according to the guidelines set out in the authorship guidelines on Data

2. Captions
Each figure caption should contain the following information, for each panel where they are relevant:
- a specification of the experimental system investigated (eg cell line, species name).
- the assay(s) and method(s) used to carry out the reported observations and measurements.
- an explicit mention of the biological and chemical entity(ies) that are being measured.
- an explicit mention of the biological and chemical entity(ies) that are altered/varied/perturbed in a controlled manner.
- the exact sample size (n) for each experimental group/condition, given as a number, not a range;
- a description of the sample collection allowing the reader to understand whether the samples represent technical or biological replicates (including how many animals, litters, cultures, etc.).
- a statement of how many times the experiment shown was independently replicated in the laboratory.
- definitions of statistical methods and measures:
  - common tests, such as t-test (please specify whether paired vs. unpaired), simple χ2 tests, Wilcoxon and Mann-Whitney tests, can be unambiguously identified by name only, but more complex techniques should be described in the methods section;
  - are tests one-sided or two-sided?
  - are there adjustments for multiple comparisons?
  - exact statistical test results, e.g., P values = x but not P values < x;
  - definition of 'center values' as median or average;
  - definition of error bars as s.d. or s.e.m.

Please complete ALL of the questions below.
Select "Not Applicable" only when the requested information is not relevant for your study.

Materials

Newly Created Materials	Information included in the manuscript?	In which section is the information available? (Reagents and Tools Table, Materials and Methods, Figures, Data Availability Section)
New materials and reagents need to be available; do any restrictions apply?	Not Applicable	

Antibodies	Information included in the manuscript?	In which section is the information available? (Reagents and Tools Table, Materials and Methods, Figures, Data Availability Section)
For **antibodies** provide the following information:<br>- Commercial antibodies: RRID (if possible) or supplier name, catalogue number and or/clone number<br>- Non-commercial: RRID or citation	Yes	Reagents and Tools Table (Antibodies).

DNA and RNA sequences	Information included in the manuscript?	In which section is the information available? (Reagents and Tools Table, Materials and Methods, Figures, Data Availability Section)
**Short novel DNA or RNA including primers, probes**: provide the sequences.	Yes	Reagents and Tools Table (Oligonucleotides and other sequence-based reagents)

Cell materials	Information included in the manuscript?	In which section is the information available? (Reagents and Tools Table, Materials and Methods, Figures, Data Availability Section)
**Cell lines:** Provide species information, strain. Provide accession number in repository **OR** supplier name, catalog number, clone number, and/**OR** RRID.	Yes	Reagents and Tools Table (Experimental Models)
**Primary cultures:** Provide species, strain, sex of origin, genetic modification status.	Not Applicable	
Report if the cell lines were recently **authenticated** (e.g., by STR profiling) and tested for mycoplasma contamination.	Yes	Methods (Cell Culture): Cell lines were authenticated using the short tandem repeat (STR) method. All cell lines were periodically assessed to ensure they are free from mycoplasma contamination through the Mycoplasma PCR detecting method.

Experimental animals	Information included in the manuscript?	In which section is the information available? (Reagents and Tools Table, Materials and Methods, Figures, Data Availability Section)
**Laboratory animals or Model organisms:** Provide species, strain, sex, age, genetic modification status. Provide accession number in repository **OR** supplier name, catalog number, clone number, **OR** RRID.	Yes	1. Methods (Animals, Generation of xenograft and homograft model): 6-8-week-old BALB/c nude mice were purchased from Shanghai SLAC Laboratory Animal company.<br>2. Reagents and Tools Table (Experimental Models)
**Animal observed in or captured from the field:** Provide species, sex, and age where possible.	Not Applicable	
Please detail **housing and husbandry conditions**.	Yes	Methods (Animals, Generation of xenograft and homograft model) : Animals were housed within a specific pathogen-free facility, maintained at a temperature range of 20-22 °C. The environment was regulated to ensure a 12-hour light and 12-hour dark cycle, with relative humidity levels kept between 50% and 60%. All animals had free access to food and water throughout the study.

Plants and microbes	Information included in the manuscript?	In which section is the information available? (Reagents and Tools Table, Materials and Methods, Figures, Data Availability Section)
**Plants:** provide species and strain, ecotype and cultivar where relevant, unique accession number if available, and source (including location for collected wild specimens).	Not Applicable	
**Microbes:** provide species and strain, unique accession number if available, and source.	Not Applicable	

Human research participants	Information included in the manuscript?	In which section is the information available? (Reagents and Tools Table, Materials and Methods, Figures, Data Availability Section)
If collected and within the bounds of privacy constraints report on age, sex and gender or ethnicity for all study participants.	Not Applicable	

Core facilities	Information included in the manuscript?	In which section is the information available? (Reagents and Tools Table, Materials and Methods, Figures, Data Availability Section)
If your work benefited from core facilities, was their service mentioned in the acknowledgments section?	Not Applicable	

Design

Study protocol	Information included in the manuscript?	In which section is the information available? (Reagents and Tools Table, Materials and Methods, Figures, Data Availability Section)
If study protocol has been **pre-registered, provide DOI in the manuscript**. For clinical trials, provide the trial registration number **OR** cite DOI.	Not Applicable	
Report the **clinical trial registration number** (at ClinicalTrials.gov or equivalent), where applicable.	Not Applicable	

Laboratory protocol	Information included in the manuscript?	In which section is the information available? (Reagents and Tools Table, Materials and Methods, Figures, Data Availability Section)
Provide DOI OR other citation details if **external detailed step-by-step protocols** are available.	Yes	1. Methods (Generation of mouse-derived organoid, Plasmids and cell Transfection): The protocols for culturing and infecting mouse breast tumor organoids have been previously described. (Nature Methods. 2018. PMID: 29256493, Nature Protocols. 2021. PMID: 33692550). 2. Methods (BN-PAGE): The protocol for blue native page has been previously described. (Nature Protocols. 2006. PMID: 17406264). 3. Methods (Complexome profiling): The protocol for complexome profiling has been previously described. (Frontiers in cell and developmental biology. 2022. PMID: 35096826, Cell Metabolism. 2017. PMID: 27720676).

Experimental study design and statistics	Information included in the manuscript?	In which section is the information available? (Reagents and Tools Table, Materials and Methods, Figures, Data Availability Section)
Include a statement about **sample size** estimate even if no statistical methods were used.	Not Applicable	
Were any steps taken to minimize the effects of subjective bias when allocating animals/samples to treatment (e.g. **randomization procedure**)? If yes, have they been described?	Yes	Methods (Animals, Generation of xenograft and homograft model): Female mice were randomly assigned to different groups for this study.
Include a statement about **blinding** even if no blinding was done.	Yes	Methods (Immunofluorescence analysis, Time-lapse imaging of mitotic progression, Live-cell imaging of NADPH biosensor, ATP Measurement, Measurement of ROS production, Mitochondrial membrane potential probe, Transmission electron microscopy) : To minimize bias, data collection was conducted using a double-blind methodology.
Describe **inclusion/exclusion criteria** if samples or animals were excluded from the analysis. Were the criteria pre-established?<br><br>If sample or data points were omitted from analysis, report if this was due to attrition or intentional exclusion and provide justification.	Not Applicable	
For every figure, are **statistical tests** justified as appropriate? Do the data meet the assumptions of the tests (e.g., normal distribution)? Describe any methods used to assess it. Is there an estimate of variation within each group of data? Is the variance similar between the groups that are being statistically compared?	Yes	For every figure, the statistical tests employed are justified based on the data characteristics. The data were carefully assessed to ensure they meet the necessary assumptions, such as normality distribution where applicable. To evaluate these assumptions, methods such as unpaired two-tailed Student's t-test, one-way ANOVA and two-way ANOVA were utilized when appropriate. Each group of data includes an estimate of variation, typically measured through standard deviation.

Sample definition and in-laboratory replication	Information included in the manuscript?	In which section is the information available? (Reagents and Tools Table, Materials and Methods, Figures, Data Availability Section)
In the figure legends: state number of times the experiment was **replicated** in laboratory.	Yes	Figure legends
In the figure legends: define whether data describe **technical or biological replicates**.	Yes	Figure legends

Ethics

Ethics	Information included in the manuscript?	In which section is the information available? (Reagents and Tools Table, Materials and Methods, Figures, Data Availability Section)
Studies involving **human participants**: State details of **authority granting ethics approval** (IRB or equivalent committee(s), provide reference number for approval.	Not Applicable	
Studies involving **human participants**: Include a statement confirming that **informed consent** was obtained from all subjects and that the experiments conformed to the principles set out in the WMA Declaration of Helsinki and the Department of Health and Human Services Belmont Report.	Not Applicable	
Studies involving **human participants:** For publication of **patient photos**, include a statement confirming that consent to publish was obtained.	Not Applicable	
Studies involving experimental **animals**: State details of **authority granting ethics approval** (IRB or equivalent committee(s), provide reference number for approval. Include a statement of compliance with ethical regulations.	Yes	Methods (Animals, Generation of xenograft and homograft model) : All experimental procedures involving mice were conducted according to the National Guidelines for Animal Usage in Research (China) and were approved by the Animal Research Ethics Committee at the University of Science and Technology of China (permit 2023-N (A)-393).
Studies involving **specimen and field samples:** State if relevant **permits** obtained, provide details of authority approving study; if none were required, explain why.	Not Applicable	

Dual Use Research of Concern (DURC)	Information included in the manuscript?	In which section is the information available? (Reagents and Tools Table, Materials and Methods, Figures, Data Availability Section)
Could your study fall under dual use research restrictions? Please check biosecurity documents and list of **select agents and toxins** (CDC): https://www.selectagents.gov/sat/list.htm	Not Applicable	
If you used a select agent, is the security level of the lab appropriate and reported in the manuscript?	Not Applicable	
If a study is subject to dual use research of concern regulations, is the name of the **authority granting approval and reference number** for the regulatory approval provided in the manuscript?	Not Applicable	

Reporting

The MDAR framework recommends adoption of discipline-specific guidelines, established and endorsed through community initiatives. Journals have their own policy about requiring specific guidelines and recommendations to complement MDAR.

Adherence to community standards	Information included in the manuscript?	In which section is the information available? (Reagents and Tools Table, Materials and Methods, Figures, Data Availability Section)
State if relevant guidelines or checklists (e.g., **ICMJE, MIBBI, ARRIVE, PRISMA**) have been followed or provided.	Not Applicable	
For **tumor marker prognostic studies,** we recommend that you follow the **REMARK** reporting guidelines (see link list at top right). See author guidelines, under 'Reporting Guidelines'. Please confirm you have followed these guidelines.	Not Applicable	
For **phase II and III randomized controlled trials**, please refer to the **CONSORT** flow diagram (see link list at top right) and submit the CONSORT checklist (see link list at top right) with your submission. See author guidelines, under 'Reporting Guidelines'. Please confirm you have submitted this list.	Not Applicable	

Data Availability

Data availability	Information included in the manuscript?	In which section is the information available? (Reagents and Tools Table, Materials and Methods, Figures, Data Availability Section)
Have **primary datasets** been deposited according to the journal's guidelines (see 'Data Deposition' section) and the respective accession numbers provided in the Data Availability Section?	Yes	Data availability: The original mass spectrometry data and the database search results produced in this study are available with the ProteomeXchange ID: PXD078881 (Fig. 1B), PXD078879 (Fig. 1E) and PXD079047 (Fig. 2B).
Were **human clinical and genomic datasets** deposited in a public access-controlled repository in accordance to ethical obligations to the patients and to the applicable consent agreement?	Not Applicable	
Are **computational models** that are central and integral to a study available without restrictions in a machine-readable form? Were the relevant accession numbers or links provided?	Not Applicable	
If publicly available data were reused, provide the respective **data citations in the reference list**.	Yes	1. Stumpf CR, Moreno MV, Olshen AB, Taylor BS, Ruggero D (2013) The translational landscape of the mammalian cell cycle. Mol Cell 52: 574-582<br>2. Rath S, Sharma R, Gupta R, Ast T, Chan C, Durham TJ, Goodman RP, Grabarek Z, Haas ME, Hung WHW et al (2021) MitoCarta3.0: an updated mitochondrial proteome now with sub-organelle localization and pathway annotations. Nucleic Acids Res 49: D1541-D1547
