# Supplementary material for: Mitotic assembly of respiratory supercomplexes ensures accurate cell division and fuels tumor growth
Source: EMBO Rep. 2026 Sep 23;27(20):6061–90. doi: 10.1038/s44319-026-00937-8 (PMC13694716; doi:10.1038/s44319-026-00937-8)
Supplement: Supplementary file 2 [file 44319_2026_937_MOESM8_ESM.pdf]

Expanded View Figures

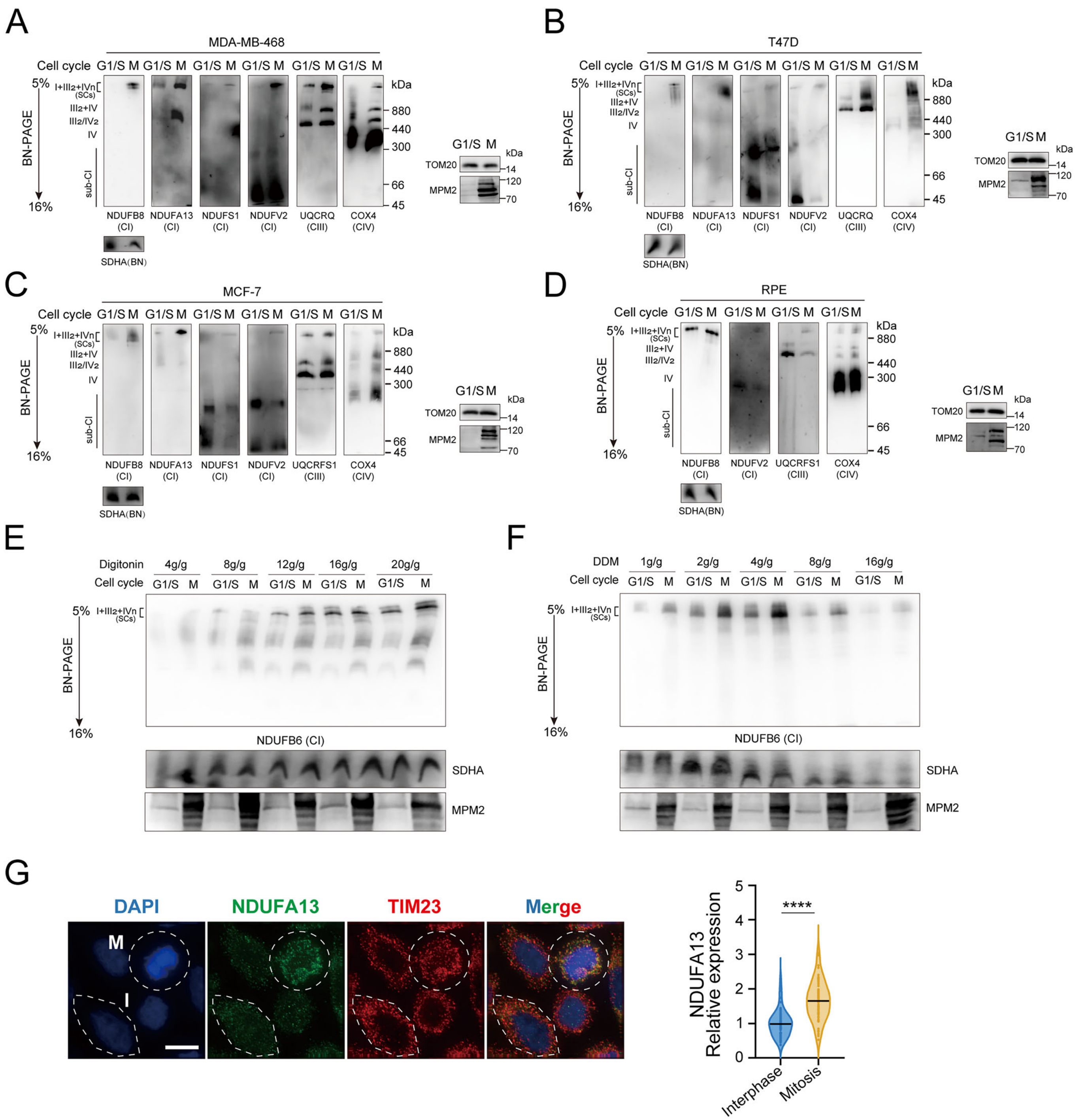

Figure EV1. Mitotic cancer cells exhibit robust upregulation of respiratory supercomplex assembly.

(**A–D**) BN-PAGE of the OXPHOS complexes in the isolated mitochondria from G1/S and mitosis MDA-MB-468 (**A**), T47D (**B**), MCF-7 (**C**), RPE (**D**) cells. Mature supercomplex forms of CI (NDUFB8, NDUFA13, NDUFS1, NDUFV2), CIII (UQCRQ or UQCRFS1), and CIV (COX4) were examined. SDHA and TOM20 were used as loading controls. MPM2 was used as a mitosis marker. (**E**) BN-PAGE of the OXPHOS complexes in the isolated mitochondria from G1/S and mitosis HeLa cells using indicated concentrations of digitonin. Mature supercomplex form of CI (NDUFB6) was examined. SDHA was used as a loading control. MPM2 was used as a mitosis marker. (**F**) BN-PAGE of the OXPHOS complexes in the isolated mitochondria from G1/S and mitosis HeLa cells using indicated concentrations of DDM. Mature supercomplex form of CI (NDUFB6) was examined. SDHA was used as a loading control. MPM2 was used as a mitosis marker. (**G**) Representative immunofluorescent images of HeLa cells stained for NDUFA13 (green), mitochondria (TIM23, red) and DNA (DAPI, blue) (left), and quantification of endogenous NDUFA13 and TIM23 in G1/S and mitosis HeLa cells ($n = 63$ biologically independent cells) (right). Data is shown as violin plots. ****$P = 1.78 \times 10^{-12}$. P values are determined using unpaired two-tailed Student's t test. Scale bar indicates 10 μm. The broken white lines highlight interphase cells (I) and mitotic cells (M).

 © The Author(s)

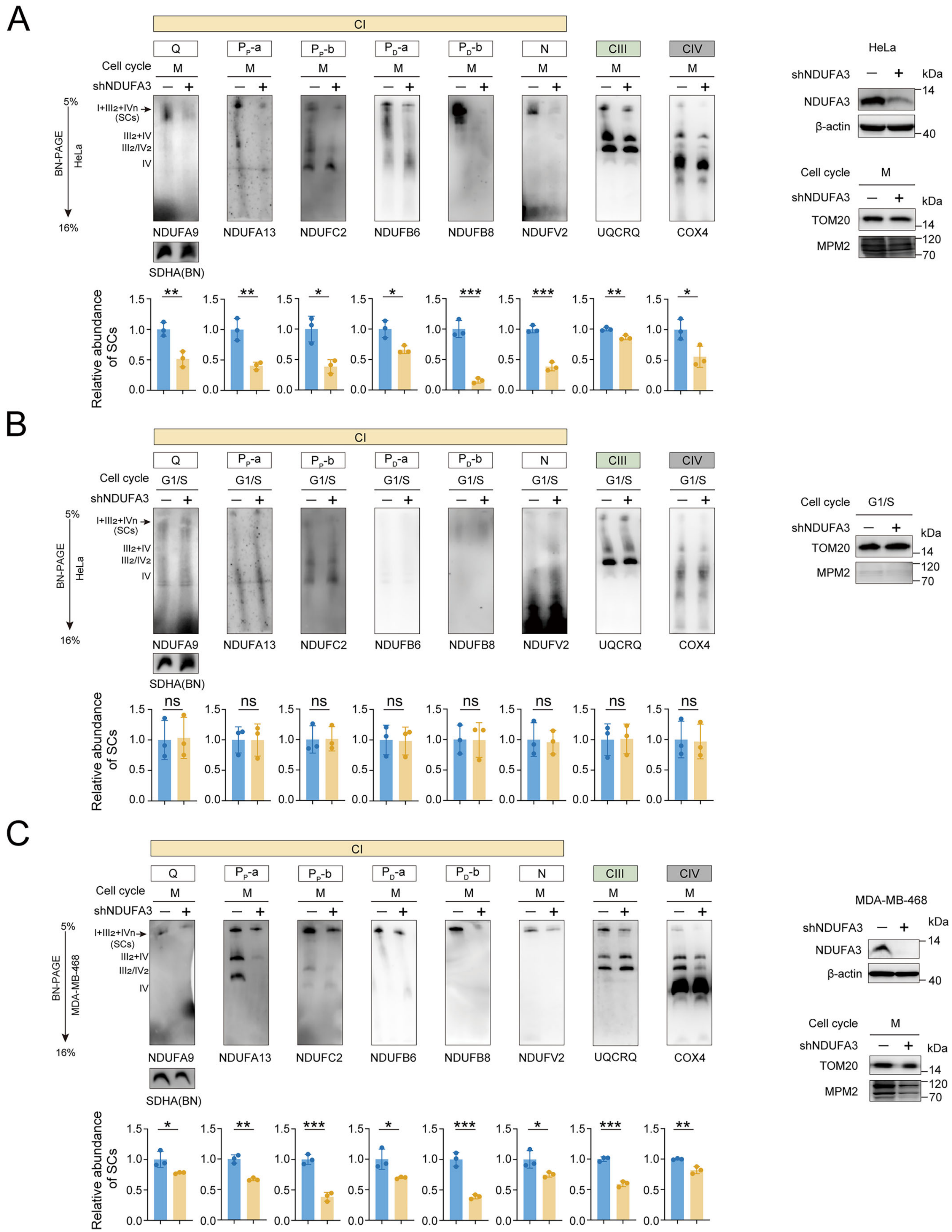
A
CI
Q
P_P-a
P_P-b
P_D-a
P_D-b
N
CIII
CIV
Cell cycle
M
shNDUFA3
− +
5%
16%
BN-PAGE
HeLa
I+III2+IVn
(SCs)
III2+IV
III2/IV2
IV
NDUFA9
NDUFA13
NDUFC2
NDUFB6
NDUFB8
NDUFV2
UQCRQ
COX4
SDHA(BN)
Relative abundance of SCs
** ** * * *** *** ** *
HeLa
NDUFA3
β-actin
TOM20
MPM2
kDa
14
40
120
70
B
G1/S
ns
C
MDA-MB-468
* ** *** * *** * *** **

Figure EV2. NDUFA3 is essential for supercomplex assembly during mitosis.

(**A**) BN-PAGE immunoblots (left) for the ETC complexes and supercomplexes of Control and NDUFA3 KD mitotic HeLa cells, shown by levels of NDUFA9 (Q), NDUFA13 (P_P-a), NDUFC2 (P_P-b), NDUFB6 (P_D-a), NDUFB8 (P_D-b), NDUFV2 (N), and UQCRQ (CIII), COX4 (CIV). Western blot analysis of cell lysates from Control or NDUFA3 KD HeLa cells with the indicated antibodies (right). SDHA and TOM20 were used as loading controls. MPM2 was used as a mitosis marker. Quantified data are presented ($n = 3$ biologically independent experiments) (bottom). Data with error bars are means ± SD. **$P = 0.0079$ (NDUFA9), **$P = 0.0057$ (NDUFA13), *$P = 0.0112$ (NDUFC2), *$P = 0.0189$ (NDUFB6), ***$P = 0.0006$ (NDUFB8), ***$P = 0.0003$ (NDUFV2), **$P = 0.0070$ (UQCRQ), *$P = 0.0330$ (COX4). P values are determined using unpaired two-tailed Student's t test. (**B**) BN-PAGE immunoblots (left) for the ETC complexes and supercomplexes of Control and NDUFA3 KD G1/S HeLa cells, shown by levels of NDUFA9 (Q), NDUFA13 (P_P-a), NDUFC2 (P_P-b), NDUFB6 (P_D-a), NDUFB8 (P_D-b), NDUFV2 (N), and UQCRQ (CIII), COX4 (CIV) (right). SDHA and TOM20 were used as loading controls. MPM2 was used as a mitosis marker. Quantified data are presented ($n = 3$ biologically independent experiments) (bottom). Data with error bars are means ± SD. (ns) $P = 0.9065$ (NDUFA9), (ns) $P = 0.9911$ (NDUFA13), (ns) $P = 0.9539$ (NDUFC2), (ns) $P = 0.9339$ (NDUFB6), (ns) $P = 0.9708$ (NDUFB8), (ns) $P = 0.8434$ (NDUFV2), (ns) $P = 0.9514$ (UQCRQ), (ns) $P = 0.8959$ (COX4). P values are determined using unpaired two-tailed Student's t test. (**C**) BN-PAGE immunoblots (left) for the ETC complexes and supercomplexes of Control and NDUFA3 KD mitotic MDA-MB-468 cells, shown by levels of NDUFA9 (Q), NDUFA13 (P_P-a), NDUFC2 (P_P-b), NDUFB6 (P_D-a), NDUFB8 (P_D-b), NDUFV2 (N), and UQCRQ (CIII), COX4 (CIV). Western blot analysis of cell lysates from Control or NDUFA3 KD MDA-MB-468 cells with the indicated antibodies (right). SDHA and TOM20 were used as loading controls. MPM2 was used as a mitosis marker. Quantified data are presented ($n = 3$ biologically independent experiments) (bottom). Data with error bars are means ± SD. *$P = 0.0445$ (NDUFA9), **$P = 0.0012$ (NDUFA13), ***$P = 0.0006$ (NDUFC2), *$P = 0.0343$ (NDUFB6), ***$P = 0.0008$ (NDUFB8), *$P = 0.0453$ (NDUFV2), ***$P = 0.0003$ (UQCRQ), **$P = 0.0061$ (COX4). P values are determined using unpaired two-tailed Student's t test.

 © The Author(s)

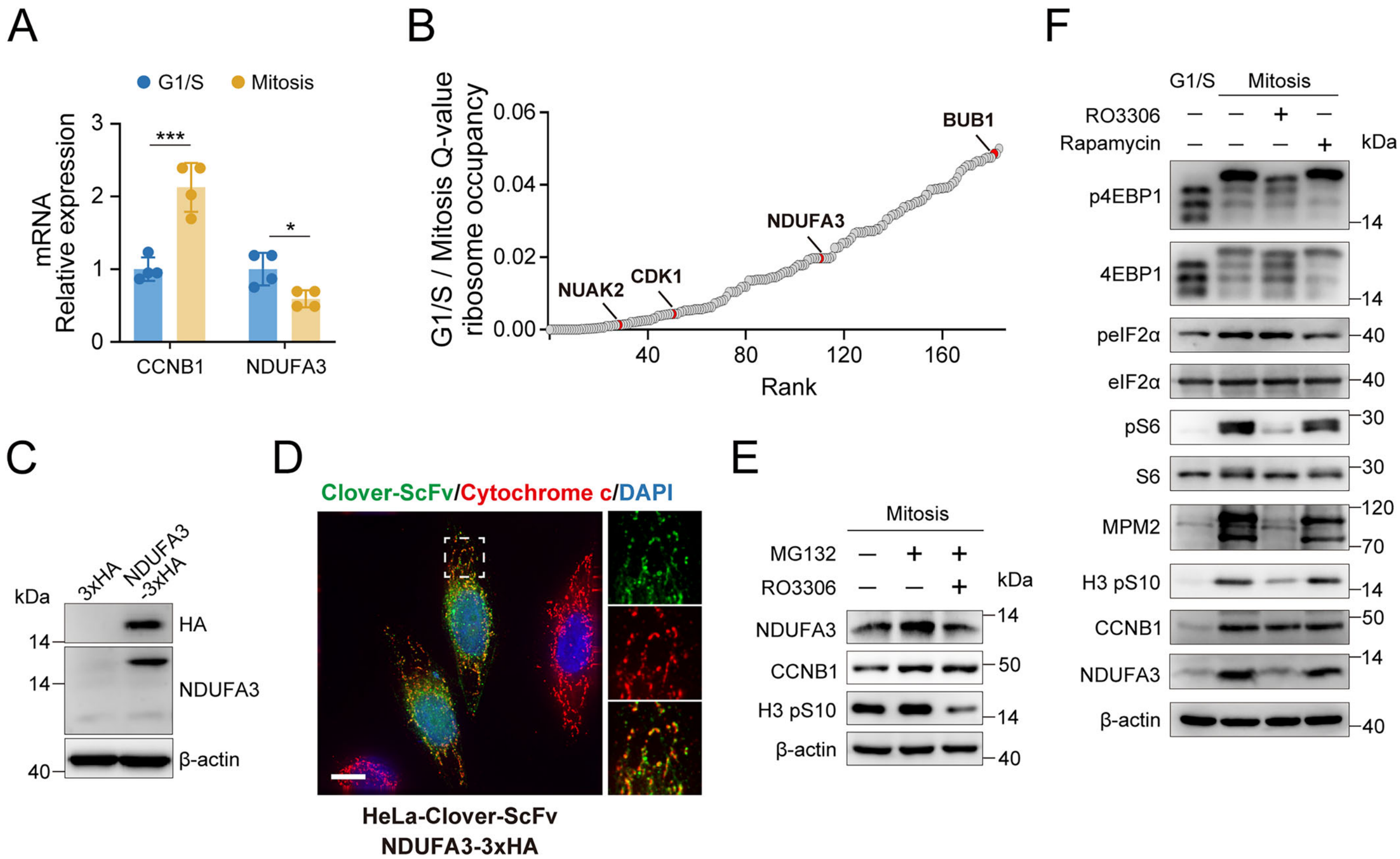

Figure EV3. CDK1 promotes NDUFA3 translation during mitosis and supports supercomplex assembly.

(**A**) The qPCR analysis of NDUFA3 mRNAs in the G1/S and mitotic HeLa cells ($n = 4$ biologically independent experiments). Data with error bars are means ± SD. ***$P = 0.0009$ (CCNB1), *$P = 0.0180$ (NDUFA3). P values are determined using unpaired two-tailed Student's t test. CCNB1 mRNA was used as a positive control. (**B**) Scatter plot of the q values of ribosome occupancy in G1/S as compared to mitotic HeLa cells(Stumpf et al, 2013). Red points denote the known proteins and NDUFA3 during mitosis. (**C**) Western blot analysis of cell lysates from 3xHA or NDUFA3-3xHA HeLa-Clover-ScFv cells with the indicated antibodies. (**D**) Representative immunofluorescent images of HeLa-Clover-ScFv-NDUFA3-3xHA cells stained for mitochondria (Cytochrome c, red) and DNA (DAPI, blue). Scale bar indicates 10 μm. (**E**) Western blot analysis of cell lysates from mitosis HeLa cells treated with or without MG132 or RO3306 with the indicated antibodies. (**F**) Western blot analysis of cell lysates from G1/S, and mitosis HeLa cells treated with inhibitors against CDK1 and mTOR, and immunoblotting of indicated proteins.

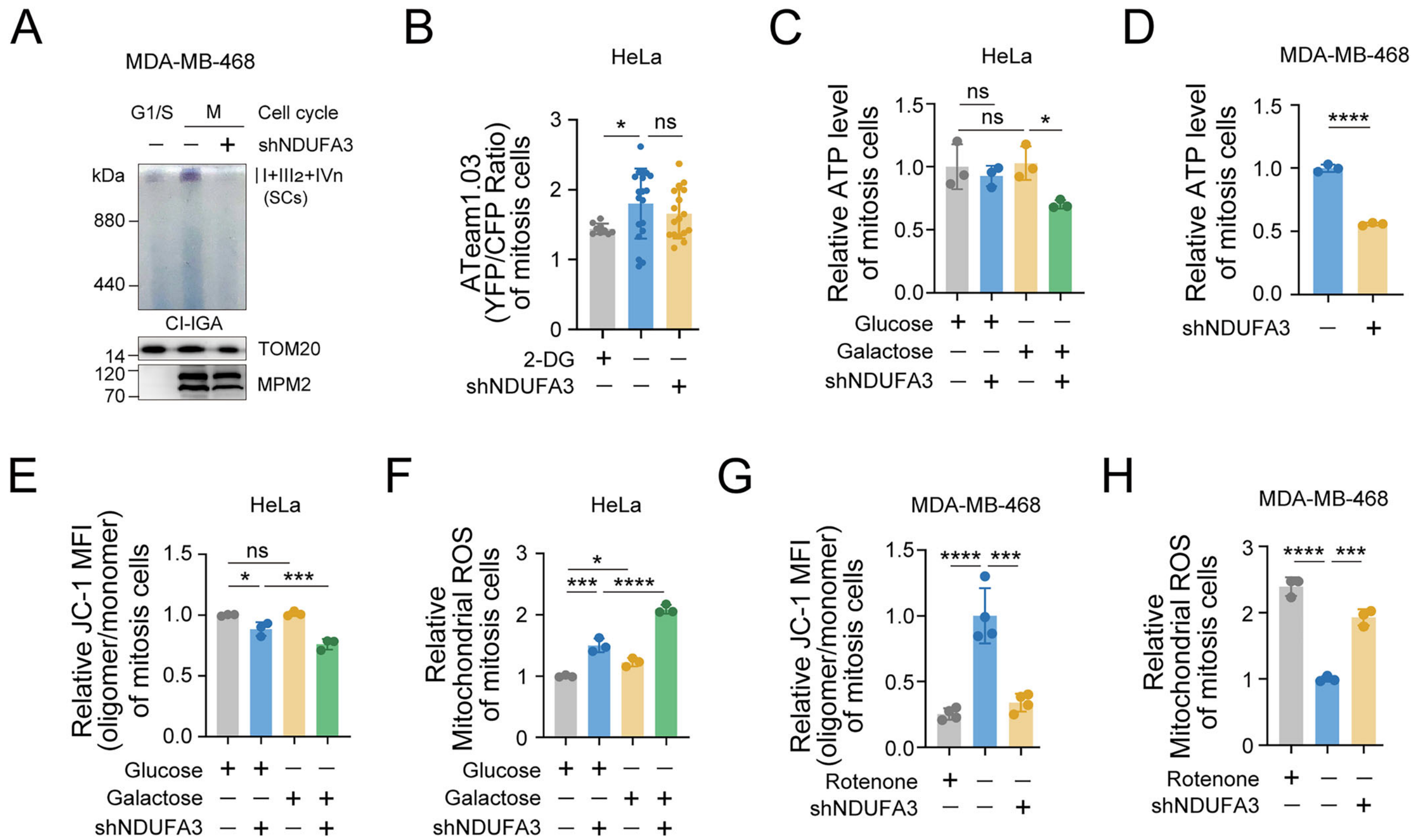

Figure EV4. NDUFA3-mediated supercomplex assembly sustains mitochondrial function and redox homeostasis during mitosis.

(**A**) In-gel activity of CI and supercomplexes of Control and NDUFA3 KD MDA-MB-468 cells. TOM20 was used as a loading control. MPM2 was used as a mitosis marker. (**B**) Quantification of ATP levels was analyzed based on the pseudocolor ratiometric images of the YFP/CFP ratio from Control or NDUFA3 KD mitotic HeLa cells expressing ATeam1.03 ($n = 9$, 18, 17 biologically independent cells, from left to right). Data with error bars are means ± SD. *$P = 0.0297$, (ns) $P = 0.2971$, from left to right. P values are determined using one-way ANOVA. Treatment with 5 mM 2-DG was used as a positive control. (**C**) Intracellular ATP concentrations of Control or NDUFA3 KD upon mitotic HeLa cells cultured under glucose or galactose conditions ($n = 3$ biologically independent experiments). Data with error bars are means ± SD. (ns) $P = 0.8741$, (ns) $P = 0.9905$, *$P = 0.0413$, from left to right. P values are determined using one-way ANOVA. (**D**) Intracellular ATP concentrations of Control or NDUFA3 KD upon mitotic MDA-MB-468 cells ($n = 3$ biologically independent experiments). Data with error bars are means ± SD. ****$P = 1.65 \times 10^{-5}$. P value was determined using unpaired two-tailed Student's t test. (**E**) Flow cytometric analysis of JC-1 in Control or NDUFA3 KD mitotic HeLa cells cultured under glucose or galactose conditions ($n = 3$ biologically independent experiments). Data with error bars are means ± SD. *$P = 0.0224$, (ns) $P = 0.9754$, ***$P = 0.0002$, from left to right. P values are determined using one-way ANOVA. (**F**) Flow cytometric analysis of MitoSOX in Control or NDUFA3 KD mitotic HeLa cells cultured under glucose or galactose conditions ($n = 3$ biologically independent experiments). Data with error bars are means ± SD. ***$P = 0.0002$, *$P = 0.0255$, ****$P = 5.74 \times 10^{-5}$, from left to right. P values are determined using one-way ANOVA. (**G**) Flow cytometric analysis of JC-1 in Control or NDUFA3 KD mitotic MDA-MB-468 cells ($n = 4$ biologically independent experiments). Data with error bars are means ± SD. ****$P = 5.20 \times 10^{-5}$, ***$P = 0.0001$, from left to right. P values are determined using one-way ANOVA. Treatment with 100 µM Rotenone was used as a positive control. (**H**) Flow cytometric analysis of MitoSOX in Control or NDUFA3 KD mitotic MDA-MB-468 cells ($n = 3$ biologically independent experiments). Data with error bars are means ± SD. ****$P = 1.06 \times 10^{-5}$, ***$P = 0.0001$, from left to right. P values are determined using one-way ANOVA. Treatment with 100 µM Rotenone was used as a positive control.

 © The Author(s)

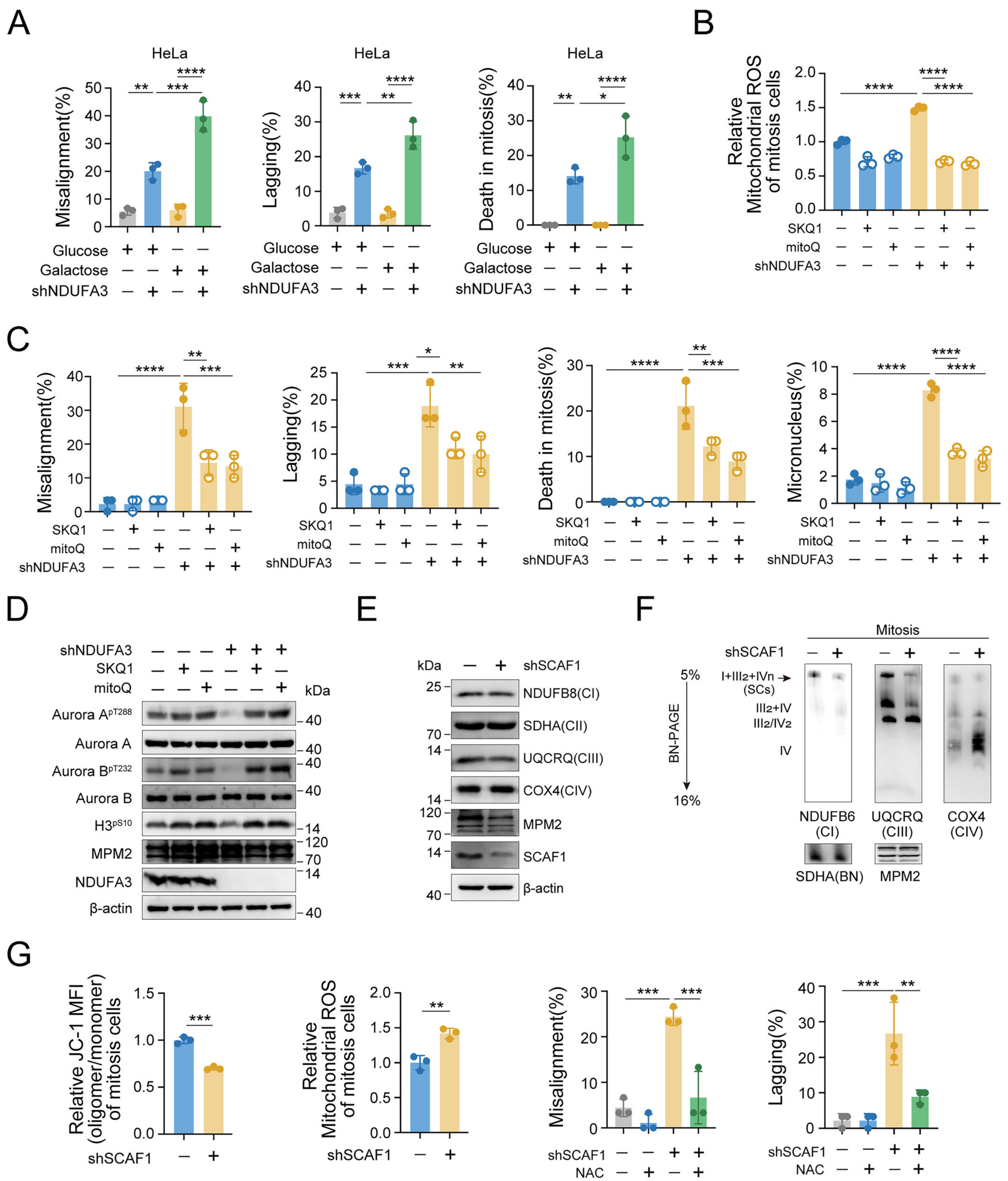

A
HeLa
Misalignment(%)
Lagging(%)
Death in mitosis(%)
Glucose
Galactose
shNDUFA3
B
Relative Mitochondrial ROS of mitosis cells
SKQ1
mitoQ
shNDUFA3
C
Misalignment(%)
Lagging(%)
Death in mitosis(%)
Micronucleus(%)
SKQ1
mitoQ
shNDUFA3
D
shNDUFA3
SKQ1
mitoQ
kDa
Aurora A pT288
Aurora A
Aurora B pT232
Aurora B
H3 pS10
MPM2
NDUFA3
β-actin
E
kDa
shSCAF1
NDUFB8(CI)
SDHA(CII)
UQCRQ(CIII)
COX4(CIV)
MPM2
SCAF1
β-actin
F
Mitosis
shSCAF1
5%
16%
BN-PAGE
I+III2+IVn (SCs)
III2+IV
III2/IV2
IV
NDUFB6 (CI)
UQCRQ (CIII)
COX4 (CIV)
SDHA(BN)
MPM2
G
Relative JC-1 MFI (oligomer/monomer) of mitosis cells
Relative Mitochondrial ROS of mitosis cells
Misalignment(%)
Lagging(%)
shSCAF1
NAC

◀ **Figure EV5. NDUFA3 depletion-induced mitotic defects are exacerbated by galactose, rescued by mitochondrial-targeted antioxidants, and recapitulated by SCAF1 knockdown.**

(**A**) Quantification of misalignment (left), lagging chromosomes (middle), and mitotic cell death (right) in Control or NDUFA3 KD HeLa cells, cultured under glucose or galactose conditions, were analyzed based on the live-cell images ($n = 3$ biologically independent experiments). Data with error bars are means ± SD. **$P = 0.0033$ (misalignment), ***$P = 0.0004$ (misalignment), ****$P = 8.15 \times 10^{-6}$ (misalignment), ***$P = 0.0008$ (lagging), **$P = 0.0056$ (lagging), ****$P = 1.31 \times 10^{-5}$ (lagging), **$P = 0.0029$ (mitotic cell death), *$P = 0.0115$ (mitotic cell death), ****$P = 5.05 \times 10^{-5}$ (mitotic cell death), from left to right. *P* values are determined using one-way ANOVA. (**B**) Flow cytometric analysis of MitoSOX in Control or NDUFA3 KD mitotic HeLa cells, treated with or without SKQ1 or mitoQ ($n = 3$ biologically independent experiments). Data with error bars are means ± SD. ****$P = 9.38 \times 10^{-8}$, ****$P = 4.59 \times 10^{-10}$, ****$P = 2.57 \times 10^{-10}$, from left to right. *P* values are determined using one-way ANOVA. (**C**) Quantification of misalignment (left), lagging chromosomes (middle-left), mitotic cell death (middle-right), and micronucleus (right) in Control or NDUFA3 KD HeLa cells, treated with or without SKQ1 or mitoQ, were analyzed based on the live-cell images ($n = 3$ biologically independent experiments). Data with error bars are means ± SD. ****$P = 6.51 \times 10^{-6}$ (misalignment), **$P = 0.0014$ (misalignment), ***$P = 0.0008$ (misalignment), ***$P = 0.0001$ (lagging), *$P = 0.0224$ (lagging), **$P = 0.0089$ (lagging), ****$P = 1.55 \times 10^{-6}$ (mitotic cell death), **$P = 0.0060$ (mitotic cell death), ***$P = 0.0004$ (mitotic cell death), ****$P = 1.95 \times 10^{-8}$ (micronucleus), ****$P = 1.20 \times 10^{-6}$ (micronucleus), ****$P = 3.90 \times 10^{-7}$ (micronucleus), from left to right. *P* values are determined using one-way ANOVA. (**D**) Western blot analysis of cell lysates from Control or NDUFA3 KD mitotic HeLa cells, treated with or without SKQ1 or mitoQ, with the indicated antibodies. (**E**) Western blot analysis of cell lysates from Control or SCAF1 KD mitotic HeLa cells with indicated proteins. (**F**) BN-PAGE immunoblots for the ETC complexes and supercomplexes of Control and SCAF1 KD mitotic HeLa cells, shown by levels of NDUFB6 (CI), UQCRQ (CIII) and COX4 (CIV). SDHA was used as a loading control. MPM2 was used as a mitosis marker. (**G**) Flow cytometric analysis of JC-1 (left) amd MitoSOX (middle-left) in Control or SCAF1 KD mitotic HeLa cells ($n = 3$ biologically independent experiments). Data with error bars are means ± SD. ***$P = 0.0002$ (JC-1), **$P = 0.0045$ (MitoSOX). *P* values are determined using unpaired two-tailed Student's *t* test. Quantification of misalignment (middle-right) and lagging chromosomes (right) in Control or SCAF1 KD HeLa cells were analyzed based on the live-cell imaging ($n = 3$ biologically independent experiments). Data with error bars are means ± SD. ***$P = 0.0004$ (misalignment), ***$P = 0.0008$ (misalignment), ***$P = 0.0010$ (lagging), **$P = 0.0074$ (lagging), from left to right. *P* values are determined using one-way ANOVA.

 © The Author(s)

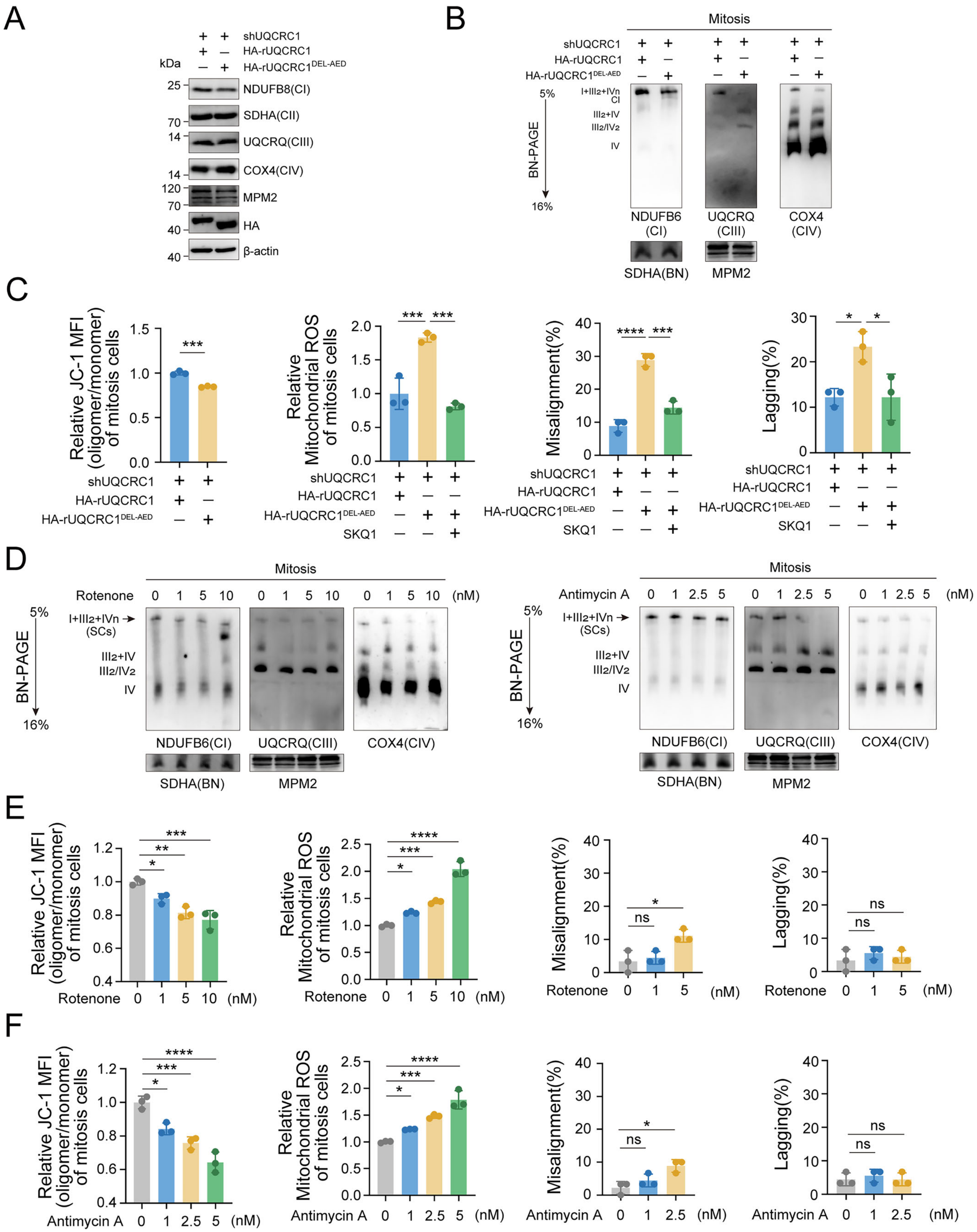
A
shUQCRC1
HA-rUQCRC1
HA-rUQCRC1DEL-AED
kDa
NDUFB8(CI)
SDHA(CII)
UQCRQ(CIII)
COX4(CIV)
MPM2
HA
β-actin
B
Mitosis
BN-PAGE
5%
16%
I+III2+IVn
CI
III2+IV
III2/IV2
IV
NDUFB6 (CI)
UQCRQ (CIII)
COX4 (CIV)
SDHA(BN)
MPM2
C
Relative JC-1 MFI (oligomer/monomer) of mitosis cells
Relative Mitochondrial ROS of mitosis cells
Misalignment(%)
Lagging(%)
SKQ1
D
Rotenone
Antimycin A
(nM)
I+III2+IVn (SCs)
NDUFB6(CI)
UQCRQ(CIII)
COX4(CIV)
E
F
ns

Figure EV6. Disruption of CI-CIII$_2$ interaction via UQCRC1$^{DEL\text{-}AED}$, but not catalytic inhibition of complex I or III, causes mitotic chromosome segregation defects.

(**A**) Western blot analysis of the indicated proteins in lysates of UQCRC1 knockdown HeLa cells complemented with shRNA-resistant HA-UQCRC1^{WT} or HA-UQCRC1$^{DEL\text{-}AED}$. (**B**) BN-PAGE immunoblots for the ETC complexes and supercomplexes of UQCRC1 knockdown HeLa cells complemented with shRNA-resistant HA-UQCRC1^{WT} or HA-UQCRC1$^{DEL\text{-}AED}$, shown by levels of NDUFB6 (CI), UQCRQ (CIII) and COX4 (CIV). SDHA was used as a loading control. MPM2 was used as a mitosis marker. (**C**) Flow cytometric analysis of JC-1 (left) amd MitoSOX (middle-left) in UQCRC1 knockdown HeLa cells complemented with shRNA-resistant HA-UQCRC1^{WT} or HA-UQCRC1$^{DEL\text{-}AED}$ ($n = 3$ biologically independent experiments). Data with error bars are means ± SD. ***$P = 0.0003$ (JC-1). P values are determined using unpaired two-tailed Student's t test (JC-1). ***$P = 0.0009$ (MitoSOX), ***$P = 0.0003$ (MitoSOX), from left to right. P values are determined using one-way ANOVA (MitoSOX). Quantification of misalignment (middle-right) and lagging chromosomes (right) in UQCRC1 knockdown HeLa cells complemented with shRNA-resistant HA-UQCRC1^{WT} or HA-UQCRC1$^{DEL\text{-}AED}$ were analyzed based on the live-cell imaging ($n = 3$ biologically independent experiments). Data with error bars are means ± SD. ****$P = 3.55 \times 10^{-5}$ (misalignment), ***$P = 0.0002$ (misalignment), *$P = 0.0237$ (lagging), *$P = 0.0237$ (lagging), from left to right. P values are determined using one-way ANOVA. (**D**) BN-PAGE immunoblots for the ETC complexes and supercomplexes in mitotic HeLa cells treated with rotenone (left) or antimycin A (right), shown by levels of NDUFB6 (CI), UQCRQ (CIII) and COX4 (CIV). SDHA was used as a loading control. MPM2 was used as a mitosis marker. (**E**) Flow cytometric analysis of JC-1 (left) amd MitoSOX (middle-left) in mitotic HeLa cells treated with rotenone ($n = 3$ biologically independent experiments). Data with error bars are means ± SD. *$P = 0.0452$ (JC-1), **$P = 0.0014$ (JC-1), ***$P = 0.0003$ (JC-1), *$P = 0.0166$ (MitoSOX), ***$P = 0.0004$ (MitoSOX), ****$P = 5.17 \times 10^{-7}$ (MitoSOX), from left to right. P values are determined using one-way ANOVA. Quantification of misalignment (middle-right) and lagging chromosomes (right) in HeLa cells treated with rotenone were analyzed based on the live-cell images ($n = 3$ biologically independent experiments). Data with error bars are means ± SD. (ns) $P = 0.8514$ (misalignment), *$P = 0.0202$ (misalignment), (ns) $P = 0.5508$ (lagging), (ns) $P = 0.8515$ (lagging), from left to right. P values are determined using one-way ANOVA. (**F**) Flow cytometric analysis of JC-1 (left) amd MitoSOX (middle-left) in mitotic HeLa cells treated with antimycin A ($n = 3$ biologically independent experiments). Data with error bars are means ± SD. *$P = 0.0101$ (JC-1), ***$P = 0.0007$ (JC-1), ****$P = 4.19 \times 10^{-5}$ (JC-1), *$P = 0.0457$ (MitoSOX), ***$P = 0.0007$ (MitoSOX), ****$P = 2.03 \times 10^{-5}$ (MitoSOX), from left to right. P values are determined using one-way ANOVA. Quantification of misalignment (middle-right) and lagging chromosomes (right) in HeLa cells treated with antimycin A were analyzed based on the live-cell imaging ($n = 3$ biologically independent experiments). Data with error bars are means ± SD. (ns) $P = 0.3922$ (misalignment), *$P = 0.0128$ (misalignment), (ns) $P = 0.7684$ (lagging), (ns) $P > 0.9999$ (lagging), from left to right. P values are determined using one-way ANOVA.

 © The Author(s)
