# Supplementary material for: Mitotic assembly of respiratory supercomplexes ensures accurate cell division and fuels tumor growth
Source: EMBO Rep. 2026 Sep 23;27(20):6061–90. doi: 10.1038/s44319-026-00937-8 (PMC13694716; doi:10.1038/s44319-026-00937-8)
Supplement: Supplementary file 3 [file 44319_2026_937_MOESM2_ESM.zip › Figure 1/1C/Fig. 1C.pdf]

Fig 1C

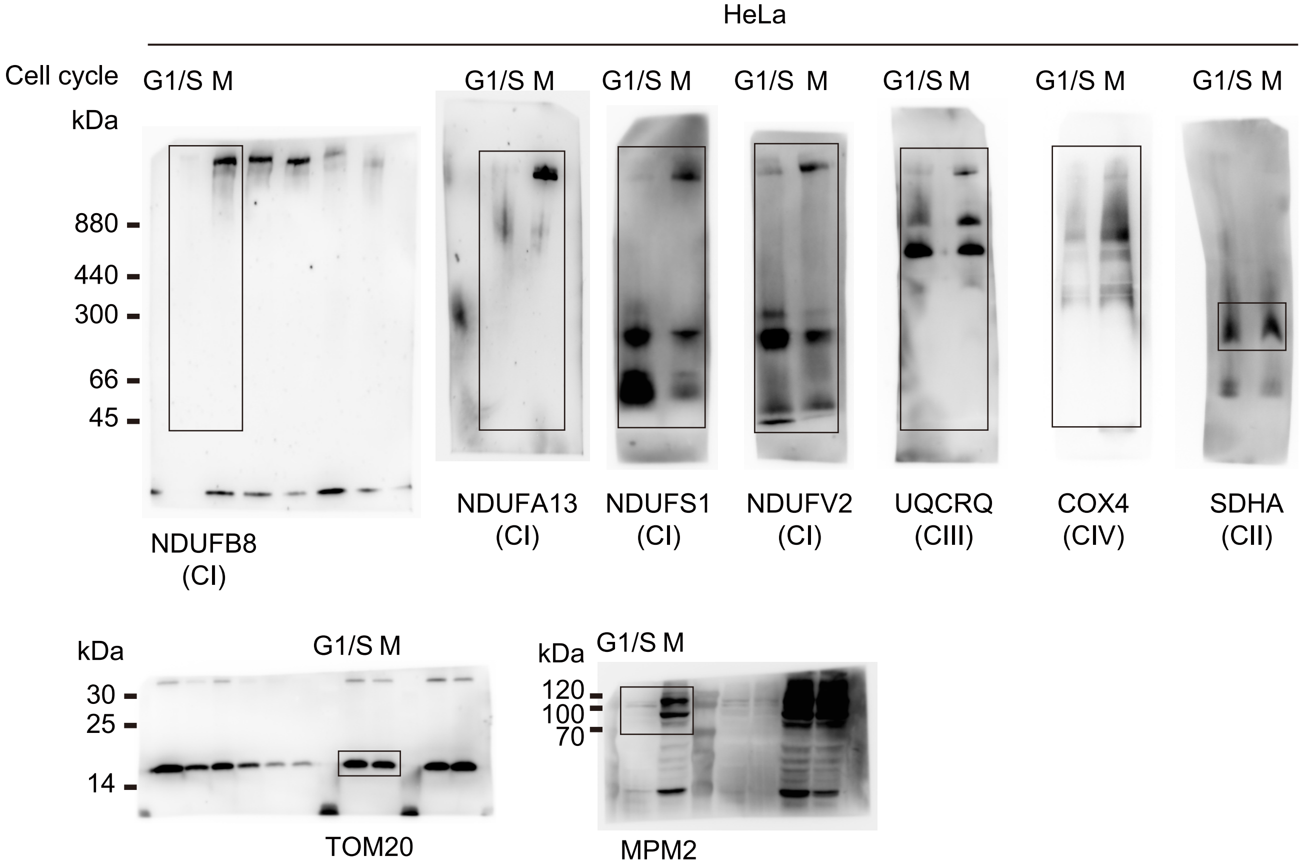
HeLa
Cell cycle
G1/S M
kDa
880
440
300
66
45
NDUFB8
(CI)
G1/S M
NDUFA13
(CI)
G1/S M
NDUFS1
(CI)
G1/S M
NDUFV2
(CI)
G1/S M
UQCRQ
(CIII)
G1/S M
COX4
(CIV)
G1/S M
SDHA
(CII)
kDa
G1/S M
30
25
14
TOM20
kDa
G1/S M
120
100
70
MPM2
