# Supplementary material for: Mitotic assembly of respiratory supercomplexes ensures accurate cell division and fuels tumor growth
Source: EMBO Rep. 2026 Sep 23;27(20):6061–90. doi: 10.1038/s44319-026-00937-8 (PMC13694716; doi:10.1038/s44319-026-00937-8)
Supplement: Supplementary file 3 [file 44319_2026_937_MOESM2_ESM.zip › Figure 1/1F/Fig. 1F.pdf]

Fig 1F

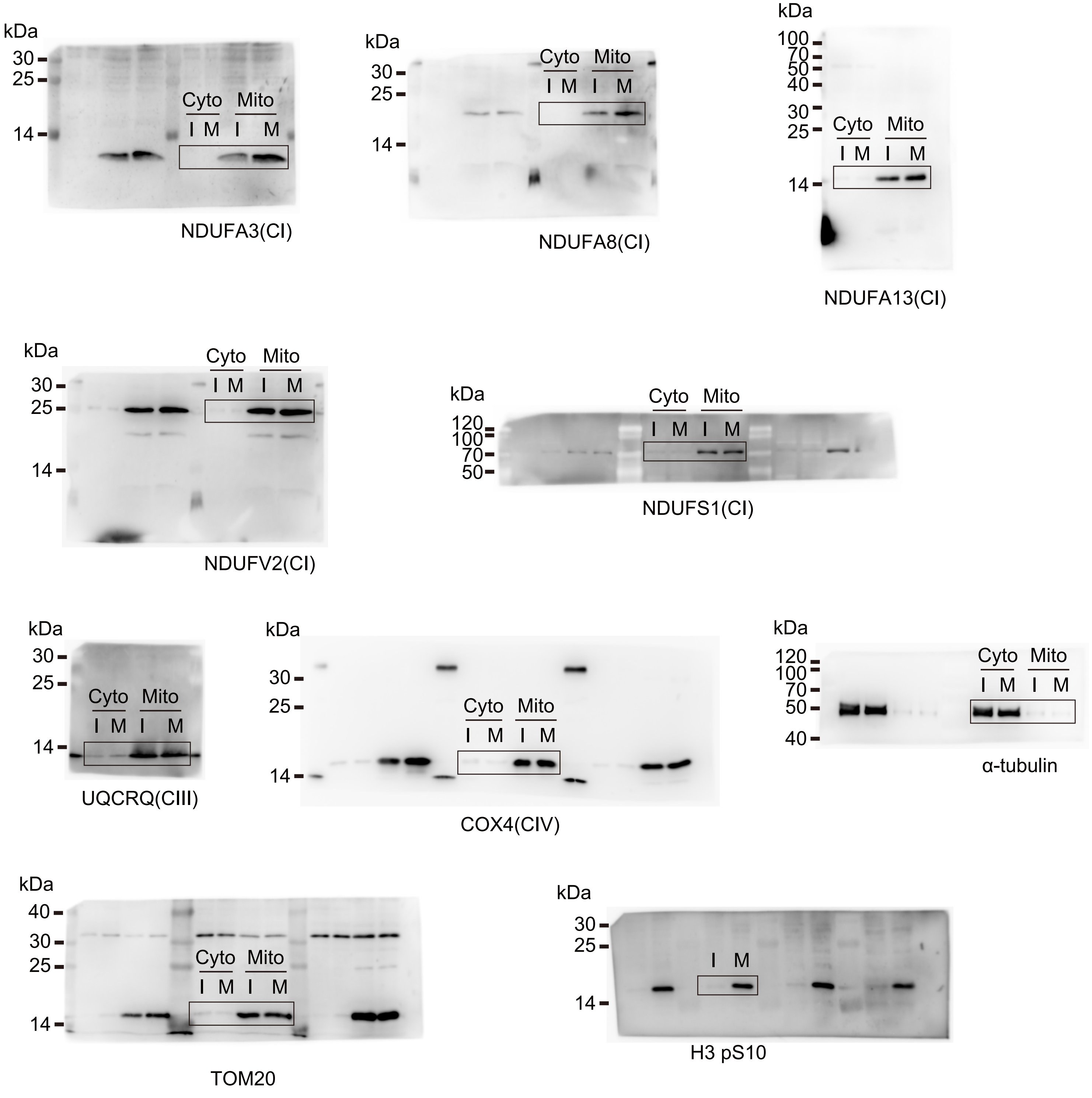
kDa
30
25
14
Cyto
Mito
I
M
I
M
NDUFA3(CI)
kDa
30
25
14
Cyto
Mito
I
M
I
M
NDUFA8(CI)
kDa
100
70
50
40
30
25
14
Cyto
Mito
I
M
I
M
NDUFA13(CI)
kDa
30
25
14
Cyto
Mito
I
M
I
M
NDUFV2(CI)
kDa
120
100
70
50
Cyto
Mito
I
M
I
M
NDUFS1(CI)
kDa
30
25
14
Cyto
Mito
I
M
I
M
UQCRQ(CIII)
kDa
30
25
14
Cyto
Mito
I
M
I
M
COX4(CIV)
kDa
120
100
70
50
40
Cyto
Mito
I
M
I
M
α-tubulin
kDa
40
30
25
14
Cyto
Mito
I
M
I
M
TOM20
kDa
30
25
14
I
M
H3 pS10
