# Supplementary material for: Mitotic assembly of respiratory supercomplexes ensures accurate cell division and fuels tumor growth
Source: EMBO Rep. 2026 Sep 23;27(20):6061–90. doi: 10.1038/s44319-026-00937-8 (PMC13694716; doi:10.1038/s44319-026-00937-8)
Supplement: Supplementary file 4 [file 44319_2026_937_MOESM3_ESM.zip › Figure 2/2A/Fig. 2A.pdf]

Fig 2A

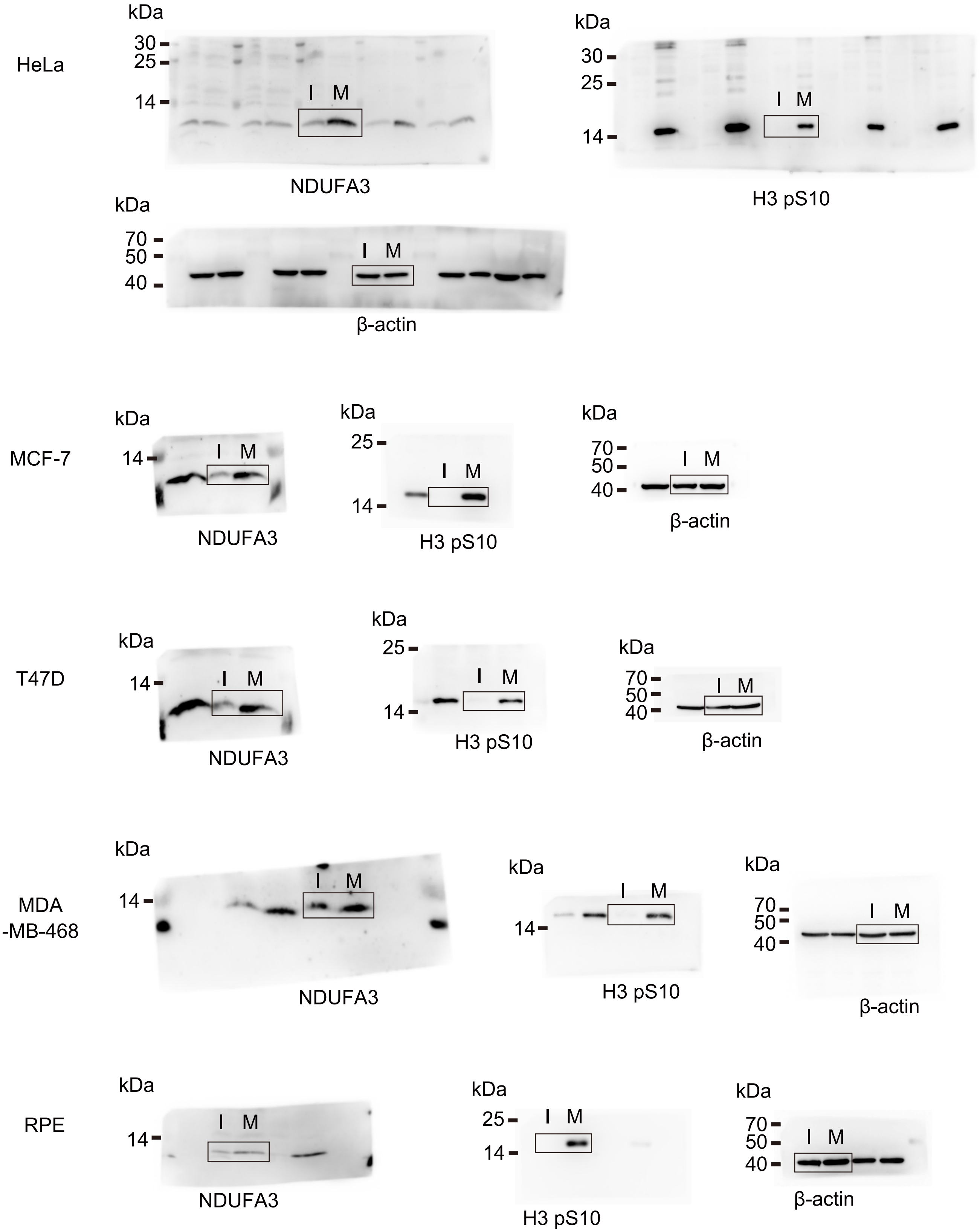
HeLa
kDa
30
25
14
I M
NDUFA3
kDa
30
25
14
I M
H3 pS10
kDa
70
50
40
I M
β-actin
MCF-7
kDa
14
I M
NDUFA3
kDa
25
14
I M
H3 pS10
kDa
70
50
40
I M
β-actin
T47D
kDa
14
I M
NDUFA3
kDa
25
14
I M
H3 pS10
kDa
70
50
40
I M
β-actin
MDA
-MB-468
kDa
14
I M
NDUFA3
kDa
14
I M
H3 pS10
kDa
70
50
40
I M
β-actin
RPE
kDa
14
I M
NDUFA3
kDa
25
14
I M
H3 pS10
kDa
70
50
40
I M
β-actin
