# Supplementary material for: Mitotic assembly of respiratory supercomplexes ensures accurate cell division and fuels tumor growth
Source: EMBO Rep. 2026 Sep 23;27(20):6061–90. doi: 10.1038/s44319-026-00937-8 (PMC13694716; doi:10.1038/s44319-026-00937-8)
Supplement: Supplementary file 4 [file 44319_2026_937_MOESM3_ESM.zip › Figure 2/2C/Fig. 2C.pdf]

Fig 2C

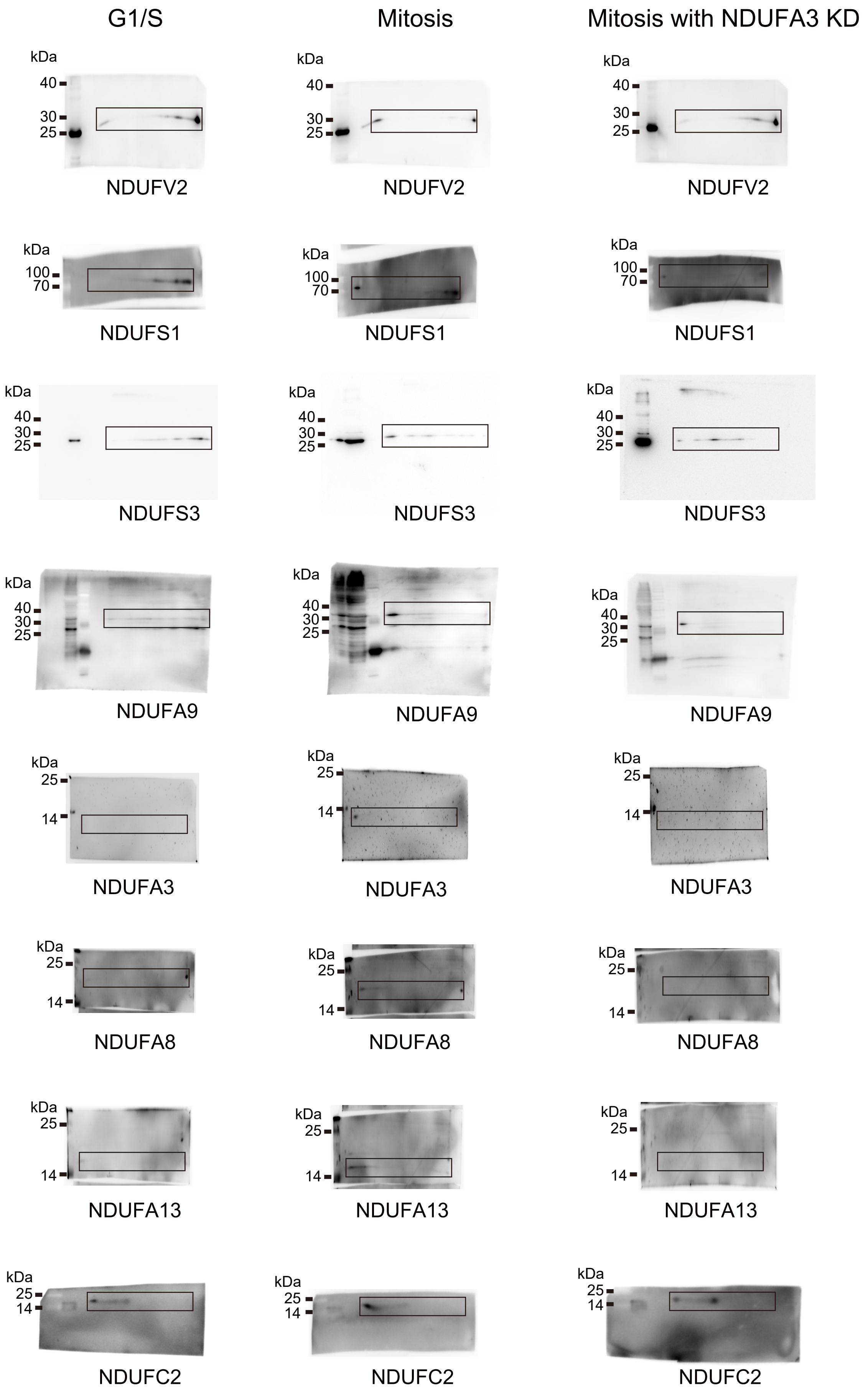
G1/S
Mitosis
Mitosis with NDUFA3 KD
kDa
40
30
25
NDUFV2
100
70
NDUFS1
NDUFS3
NDUFA9
14
NDUFA3
NDUFA8
NDUFA13
NDUFC2

Fig 2C

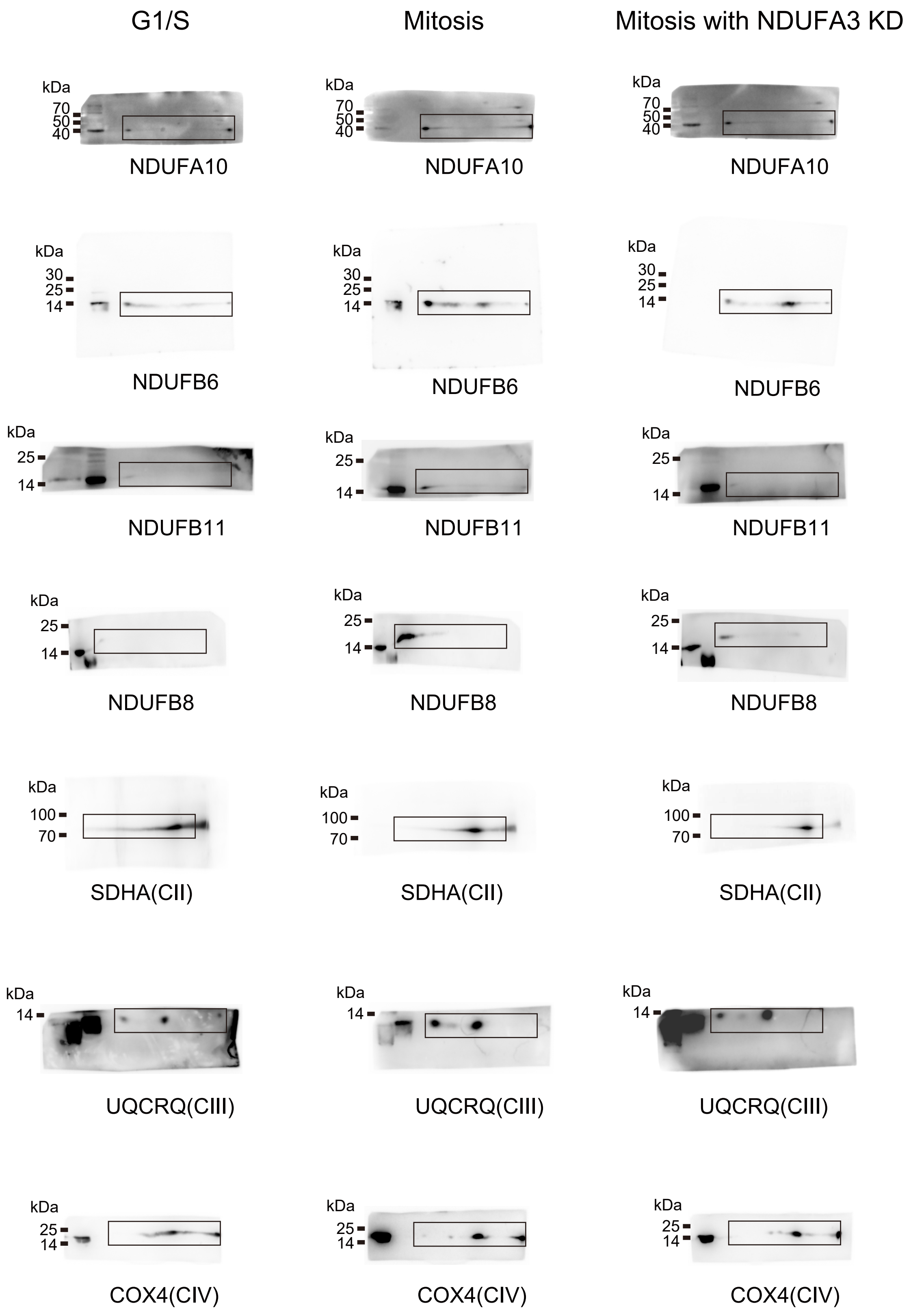

G1/S
Mitosis
Mitosis with NDUFA3 KD
kDa
70
50
40
NDUFA10
NDUFA10
NDUFA10
30
25
14
NDUFB6
NDUFB6
NDUFB6
NDUFB11
NDUFB11
NDUFB11
NDUFB8
NDUFB8
NDUFB8
100
SDHA(CII)
SDHA(CII)
SDHA(CII)
UQCRQ(CIII)
UQCRQ(CIII)
UQCRQ(CIII)
COX4(CIV)
COX4(CIV)
COX4(CIV)
