# Supplementary material for: Mitotic assembly of respiratory supercomplexes ensures accurate cell division and fuels tumor growth
Source: EMBO Rep. 2026 Sep 23;27(20):6061–90. doi: 10.1038/s44319-026-00937-8 (PMC13694716; doi:10.1038/s44319-026-00937-8)
Supplement: Supplementary file 4 [file 44319_2026_937_MOESM3_ESM.zip › Figure 2/2E/Fig. 2E.pdf]

Fig 2E

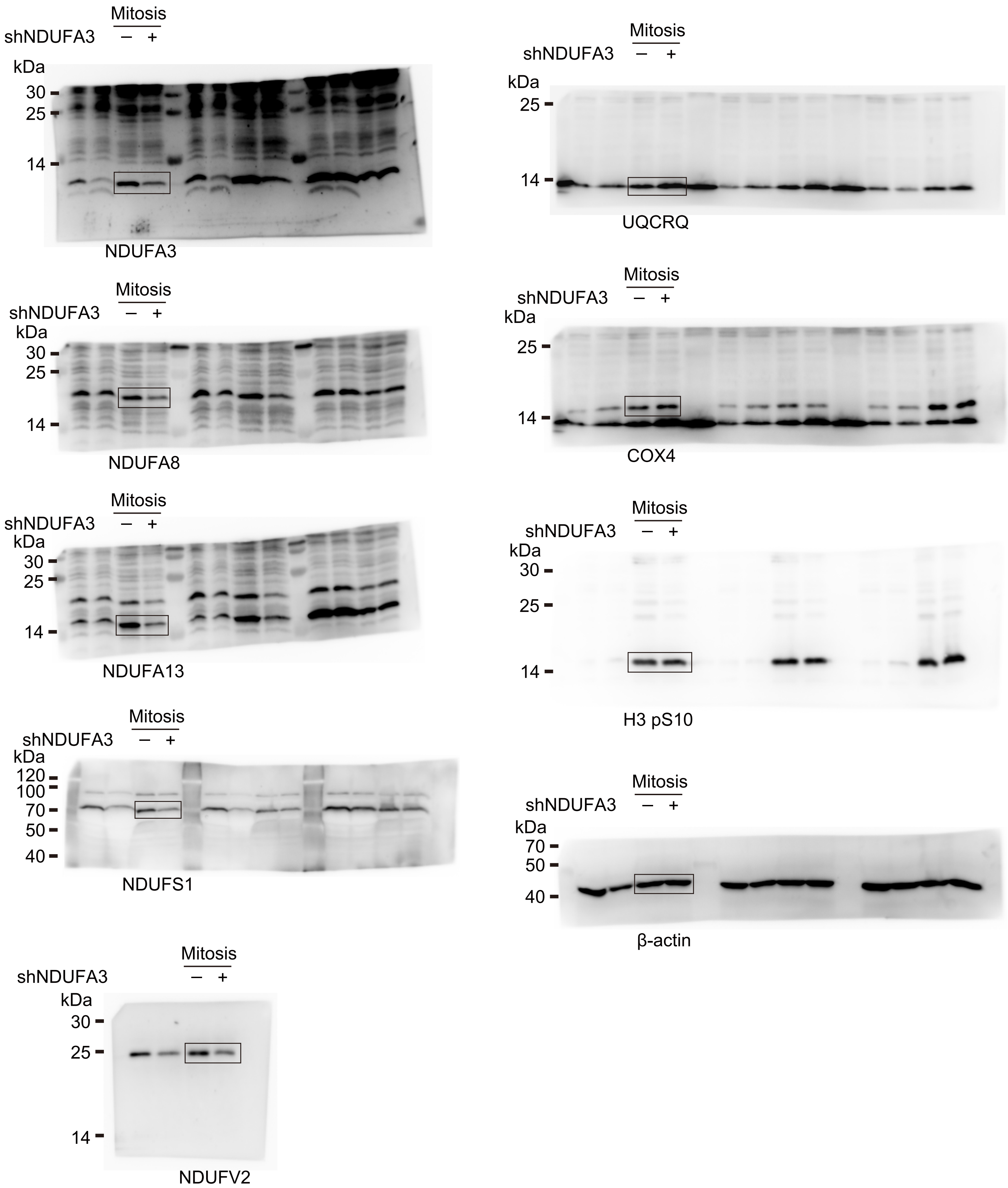

Mitosis
shNDUFA3 – +
kDa
30
25
14
NDUFA3
Mitosis
shNDUFA3 – +
kDa
30
25
14
NDUFA8
Mitosis
shNDUFA3 – +
kDa
30
25
14
NDUFA13
Mitosis
shNDUFA3 – +
kDa
120
100
70
50
40
NDUFS1
Mitosis
shNDUFA3 – +
kDa
30
25
14
NDUFV2
Mitosis
shNDUFA3 – +
kDa
25
14
UQCRQ
Mitosis
shNDUFA3 – +
kDa
25
14
COX4
Mitosis
shNDUFA3 – +
kDa
30
25
14
H3 pS10
Mitosis
shNDUFA3 – +
kDa
70
50
40
β-actin
