# Supplementary material for: Mitotic assembly of respiratory supercomplexes ensures accurate cell division and fuels tumor growth
Source: EMBO Rep. 2026 Sep 23;27(20):6061–90. doi: 10.1038/s44319-026-00937-8 (PMC13694716; doi:10.1038/s44319-026-00937-8)
Supplement: Supplementary file 5 [file 44319_2026_937_MOESM4_ESM.zip › Figure 3/3A/Fig. 3A.pdf]

Fig. 3A

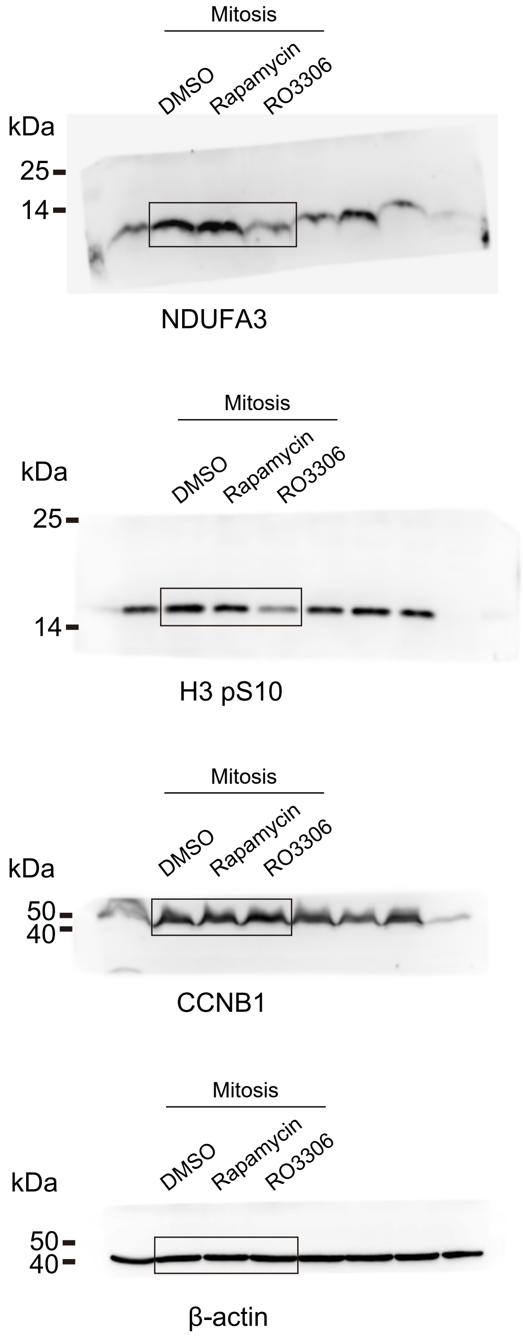

Mitosis
DMSO
Rapamycin
RO3306
kDa
25
14
NDUFA3
Mitosis
DMSO
Rapamycin
RO3306
kDa
25
14
H3 pS10
Mitosis
DMSO
Rapamycin
RO3306
kDa
50
40
CCNB1
Mitosis
DMSO
Rapamycin
RO3306
kDa
50
40
β-actin
