# Supplementary material for: Mitotic assembly of respiratory supercomplexes ensures accurate cell division and fuels tumor growth
Source: EMBO Rep. 2026 Sep 23;27(20):6061–90. doi: 10.1038/s44319-026-00937-8 (PMC13694716; doi:10.1038/s44319-026-00937-8)
Supplement: Supplementary file 5 [file 44319_2026_937_MOESM4_ESM.zip › Figure 3/3E/Fig. 3E.pdf]

Fig. 3E

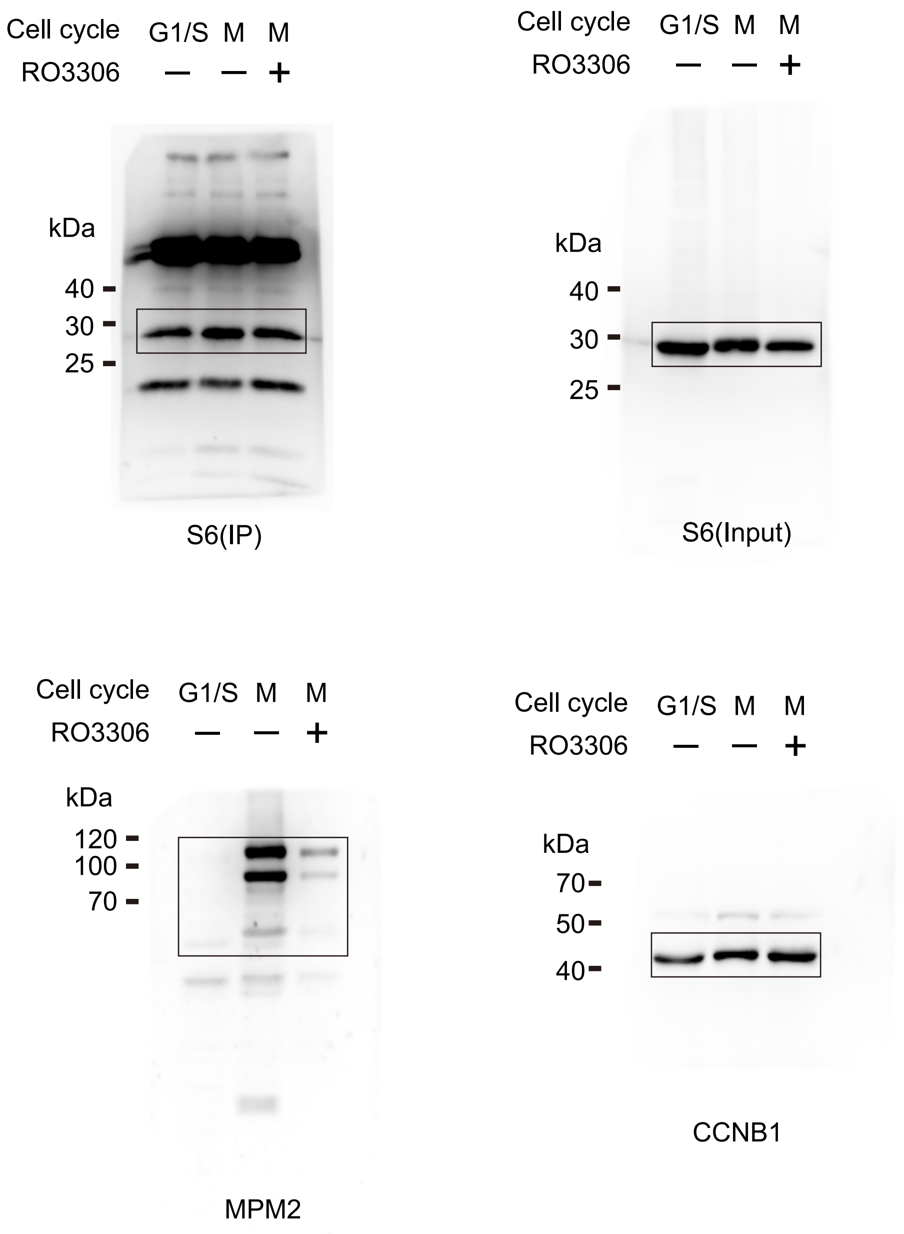

Cell cycle G1/S M M
RO3306 – – +
kDa
40
30
25
S6(IP)
Cell cycle G1/S M M
RO3306 – – +
kDa
40
30
25
S6(Input)
Cell cycle G1/S M M
RO3306 – – +
kDa
120
100
70
MPM2
Cell cycle G1/S M M
RO3306 – – +
kDa
70
50
40
CCNB1
