# Supplementary material for: Mitotic assembly of respiratory supercomplexes ensures accurate cell division and fuels tumor growth
Source: EMBO Rep. 2026 Sep 23;27(20):6061–90. doi: 10.1038/s44319-026-00937-8 (PMC13694716; doi:10.1038/s44319-026-00937-8)
Supplement: Supplementary file 5 [file 44319_2026_937_MOESM4_ESM.zip › Figure 3/3F/Fig. 3F.pdf]

Fig. 3F

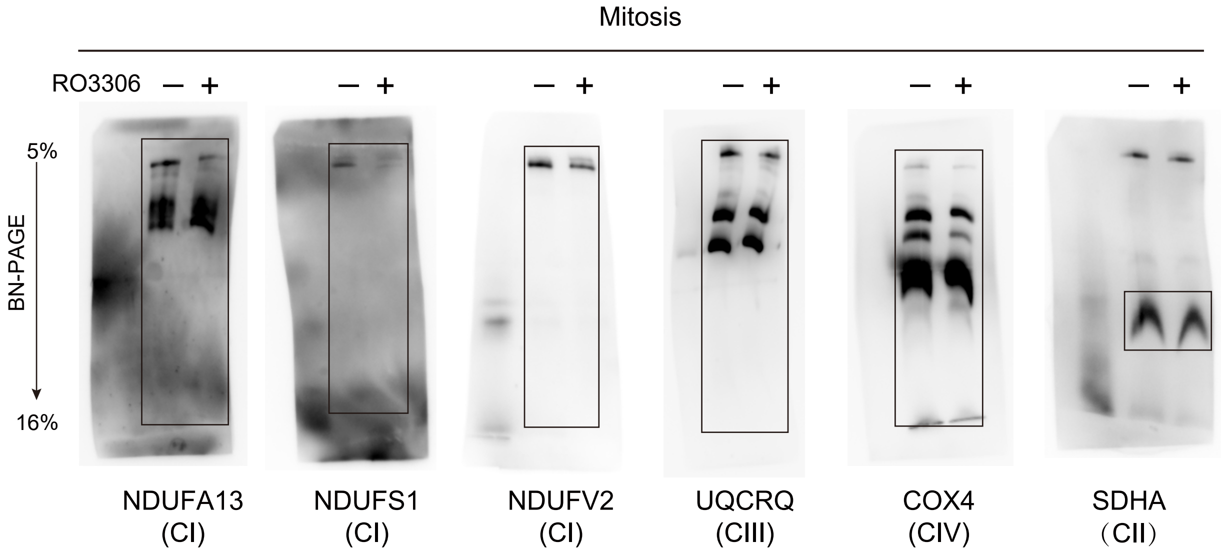

Mitosis
RO3306 — +
— +
— +
— +
— +
— +
5%
BN-PAGE
16%
NDUFA13
(CI)
NDUFS1
(CI)
NDUFV2
(CI)
UQCRQ
(CIII)
COX4
(CIV)
SDHA
(CII)

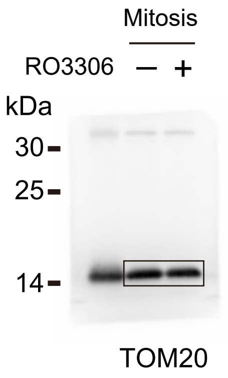

Mitosis
RO3306 — +
kDa
30
25
14
TOM20

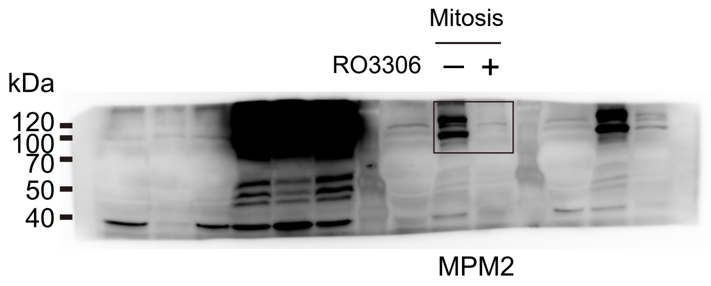

Mitosis
RO3306 — +
kDa
120
100
70
50
40
MPM2
