# Supplementary material for: Mitotic assembly of respiratory supercomplexes ensures accurate cell division and fuels tumor growth
Source: EMBO Rep. 2026 Sep 23;27(20):6061–90. doi: 10.1038/s44319-026-00937-8 (PMC13694716; doi:10.1038/s44319-026-00937-8)
Supplement: Supplementary file 6 [file 44319_2026_937_MOESM5_ESM.zip › Figure 4/4A/Fig. 4A.pdf]

Fig 4A

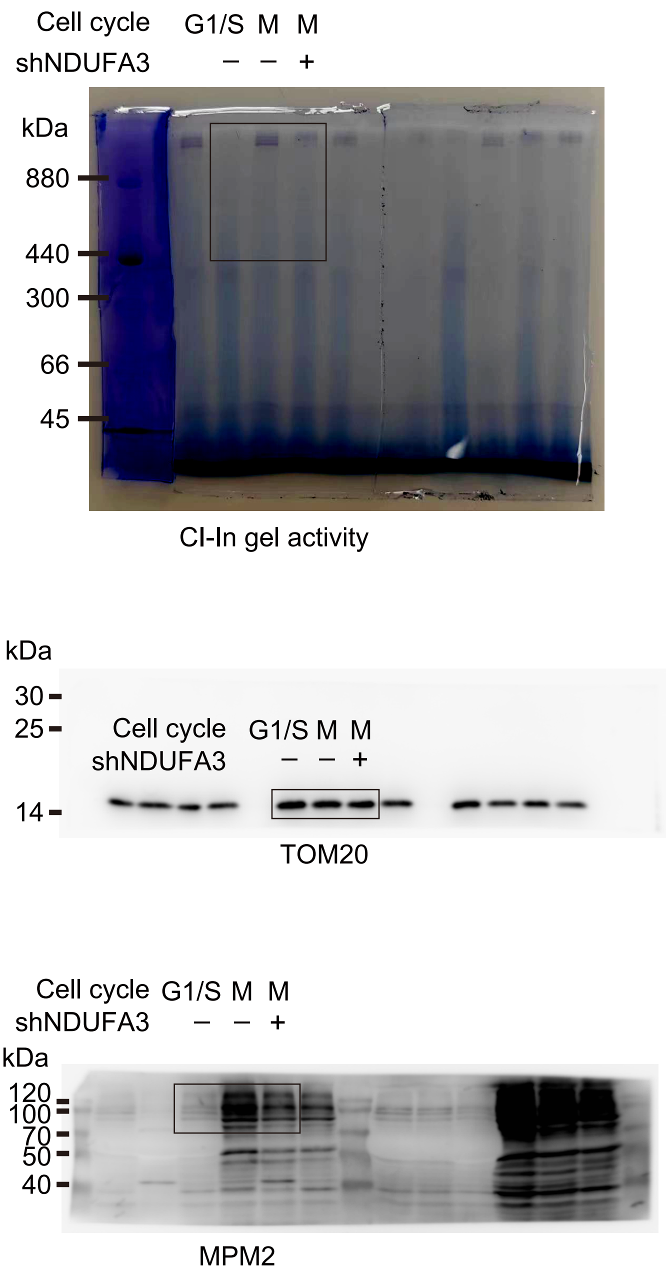
Cell cycle G1/S M M
shNDUFA3 – – +
kDa
880
440
300
66
45
CI-In gel activity
kDa
30
25
Cell cycle G1/S M M
shNDUFA3 – – +
14
TOM20
Cell cycle G1/S M M
shNDUFA3 – – +
kDa
120
100
70
50
40
MPM2
