# Supplementary material for: Mitotic assembly of respiratory supercomplexes ensures accurate cell division and fuels tumor growth
Source: EMBO Rep. 2026 Sep 23;27(20):6061–90. doi: 10.1038/s44319-026-00937-8 (PMC13694716; doi:10.1038/s44319-026-00937-8)
Supplement: Supplementary file 7 [file 44319_2026_937_MOESM6_ESM.zip › Figure 5/5D/Fig. 5D.pdf]

Fig 5D

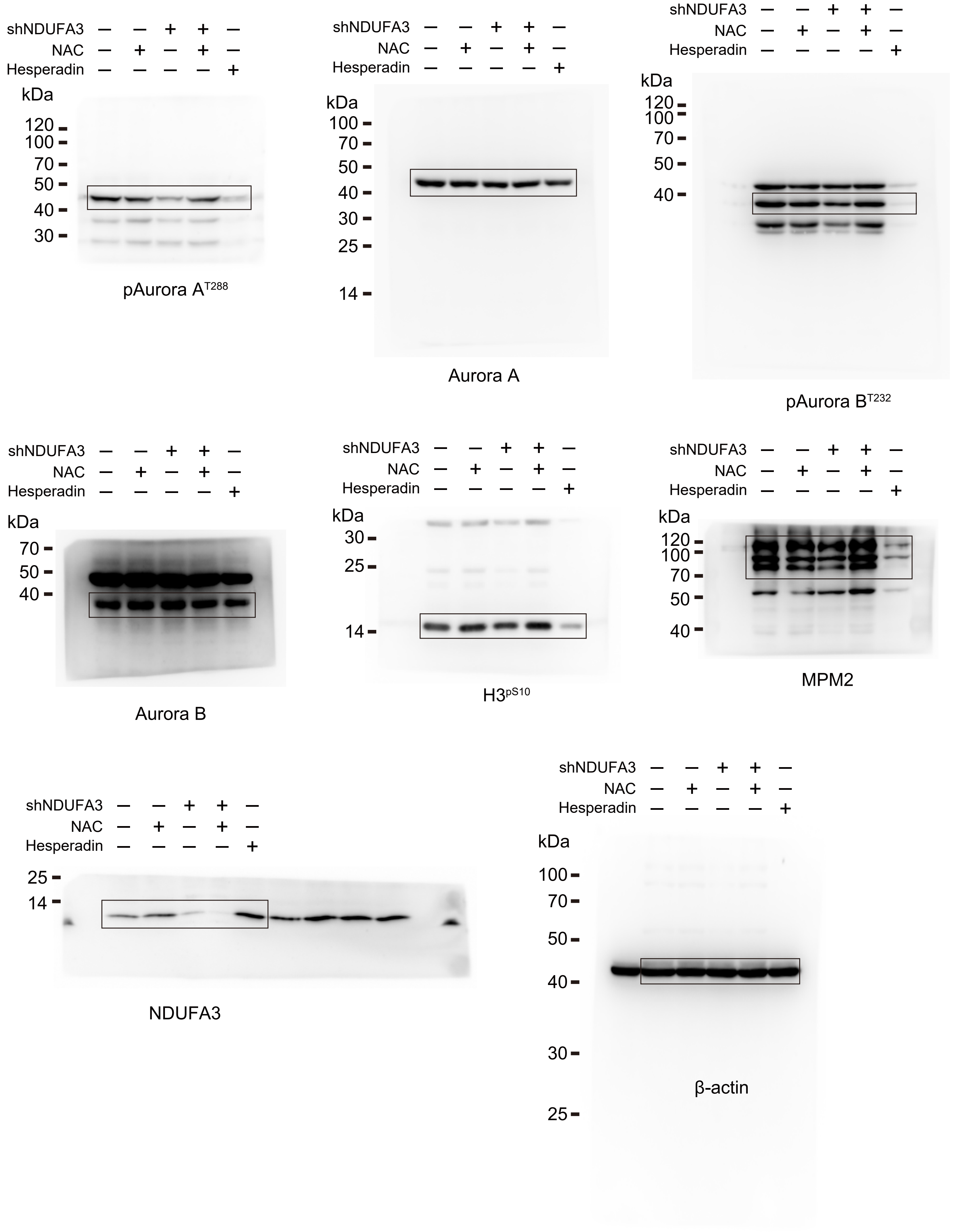

shNDUFA3 — — + + —
NAC — + — + —
Hesperadin — — — — +
kDa
120
100
70
50
40
30
pAurora A^T288
shNDUFA3 — — + + —
NAC — + — + —
Hesperadin — — — — +
kDa
100
70
50
40
30
25
14
Aurora A
shNDUFA3 — — + + —
NAC — + — + —
Hesperadin — — — — +
kDa
120
100
70
50
40
pAurora B^T232
shNDUFA3 — — + + —
NAC — + — + —
Hesperadin — — — — +
kDa
70
50
40
Aurora B
shNDUFA3 — — + + —
NAC — + — + —
Hesperadin — — — — +
kDa
30
25
14
H3^pS10
shNDUFA3 — — + + —
NAC — + — + —
Hesperadin — — — — +
kDa
120
100
70
50
40
MPM2
shNDUFA3 — — + + —
NAC — + — + —
Hesperadin — — — — +
25
14
NDUFA3
shNDUFA3 — — + + —
NAC — + — + —
Hesperadin — — — — +
kDa
100
70
50
40
30
25
β-actin
