# Supplementary material for: Mitotic assembly of respiratory supercomplexes ensures accurate cell division and fuels tumor growth
Source: EMBO Rep. 2026 Sep 23;27(20):6061–90. doi: 10.1038/s44319-026-00937-8 (PMC13694716; doi:10.1038/s44319-026-00937-8)
Supplement: Supplementary file 8 [file 44319_2026_937_MOESM7_ESM.zip › Figure 6/6A/Fig. 6A.pdf]

Fig. 6ABC

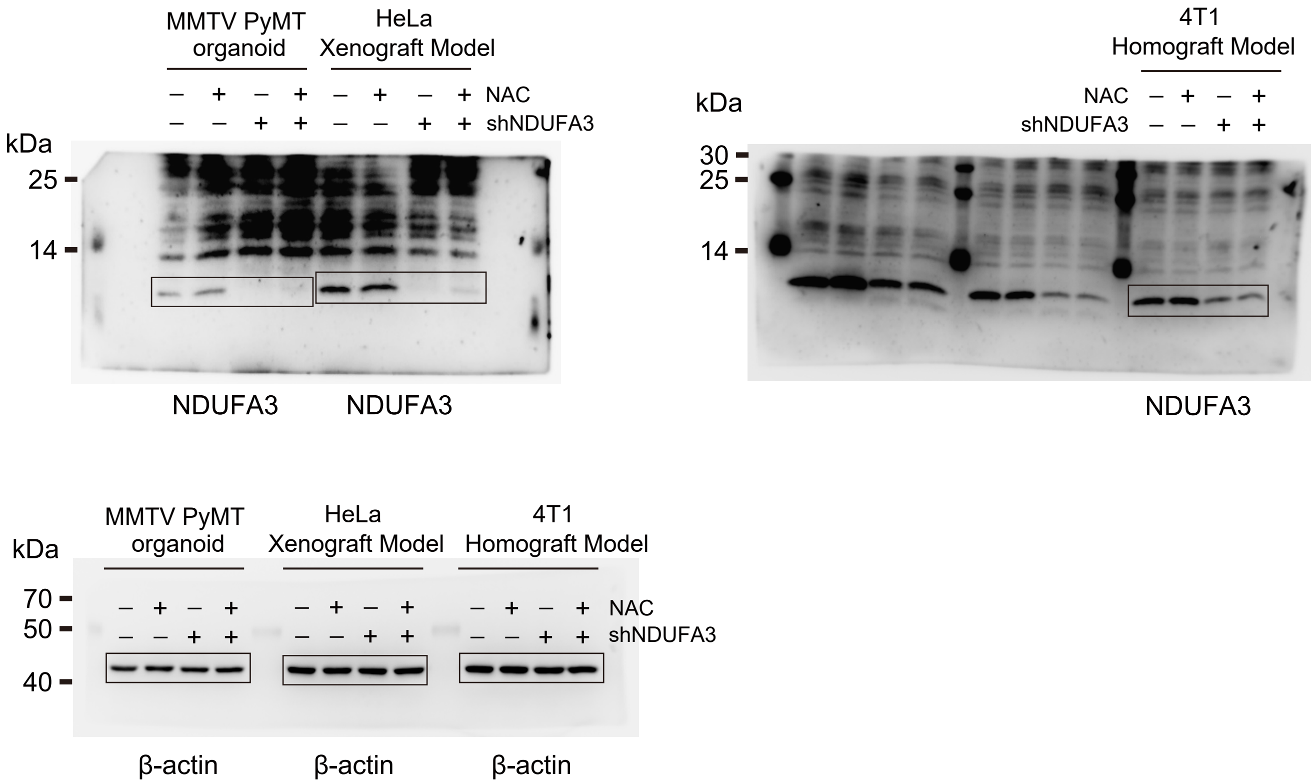
MMTV PyMT organoid
HeLa Xenograft Model
NAC
shNDUFA3
kDa
25
14
NDUFA3
NDUFA3
4T1 Homograft Model
NAC
shNDUFA3
kDa
30
25
14
NDUFA3
MMTV PyMT organoid
HeLa Xenograft Model
4T1 Homograft Model
kDa
70
50
40
NAC
shNDUFA3
β-actin
β-actin
β-actin
